# Expanding the toolbox of Baeyer–Villiger and flavin monooxygenase biocatalysts for the enantiodivergent green synthesis of sulfoxides†

**DOI:** 10.1039/d4gc02657h

**Published:** 2024-07-05

**Authors:** Jingyue Wu, Silvia Anselmi, Alexandra T. P. Carvalho, Jill Caswell, Derek J. Quinn, Thomas S. Moody, Daniele Castagnolo

**Affiliations:** a Department of Chemistry, University College London 20 Gordon Street WC1H 0AJ London UK d.castagnolo@ucl.ac.uk; b Department of Biocatalysis & Isotope Chemistry Almac 20 Seagoe Industrial Estate Craigavon BT63 5QD UK tom.moody@almacgroup.com; c Arran Chemical Company Limited, Unit 1 Monksland Industrial Estate Athlone Co. Roscommon Ireland

## Abstract

Two new monooxygenase biocatalysts, the Baeyer–Villiger monooxygenase BVMO145 and the flavin monooxygenase FMO401 from Almac library, have been found to catalyse the enantiodivergent oxidation of sulfides bearing N-heterocyclic substituents into sulfoxides under mild and green conditions. The biocatalyst BVMO145 provides (*S*)-sulfoxides while the flavin monooxygenase FMO401 affords (*R*)-sulfoxides with good conversions and high ee.

## Abstract 2

Two new monooxygenase biocatalysts, BVMO145 and FMO401 from Almac library, catalyse the enantiodivergent oxidation of sulfides bearing N-heterocyclic substituents into sulfoxides under mild and green conditions.

## Graphical abstract

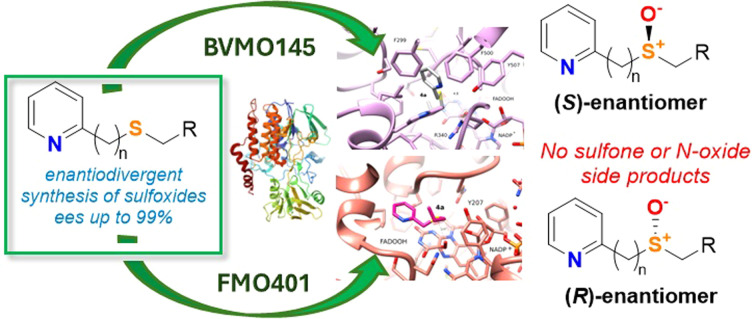

Chiral sulfoxides are a ubiquitous class of compounds that find broad applications in pharmaceutical chemistry,^1^ and they constitute the key structural motif of many pharmaceutics, including the drugs armodafinil,^2^ flosequinan and esomeprazole (Fig. 1).^3^ The absolute configuration of the sulfoxide moiety has a key impact on their chemical and biological properties. Optically pure sulfoxides also find use as catalysts, building blocks and chiral ligands in organic chemistry.^3–8^ The main approach to synthesise enantiopure sulfoxides consists of the asymmetric oxidation of a prochiral sulfide precursor. Classic oxidation methods employ metal oxidants such Ti, V, Mn or Cu in the presence of appropriate chiral ligands (*i.e.* salen ligands and chiral Schiff bases).^1,9,10^ More recently, the need to access such important compounds *via* milder and greener strategies has driven the interest of both academia and industry to exploit enzymes as biocatalysts in sulfoxidation reactions.^11^

**Fig. 1:**
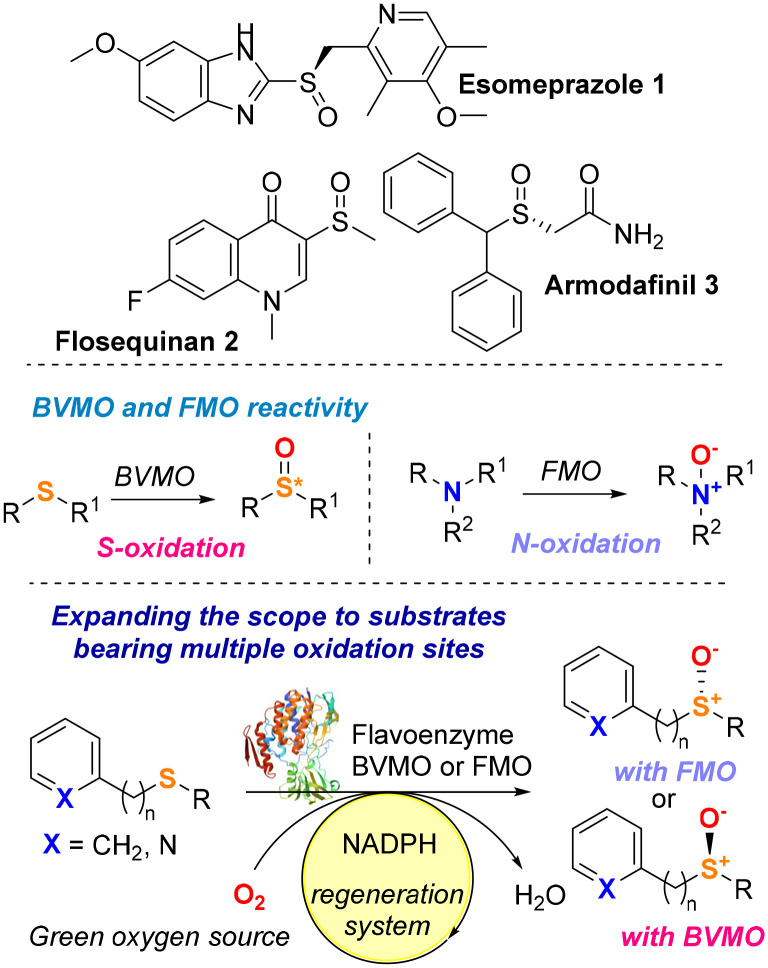
Biocatalytic approaches for the synthesis of enantiomerically pure sulfoxides.

Baeyer–Villiger monooxygenases (BVMOs) and flavin-containing monooxygenases (FMOs) are oxidative enzymes that belong to a broader class of enzymes called flavoprotein monooxygenases (FPMO). To date, there are eight subclasses of FPMOs (Groups A to H), which are differentiated by both structural and functional features.^12–15^ Enzymes belonging to Groups A and B rely on the tightly bound flavin adenosine dinucleotide (FAD) prosthetic group and NAD(P)H as electron donors for their oxidative activity. The FPMOs in Groups A and B are single-component enzymes, capable of regenerating the active site without the need for external recycling systems, making them attractive potential biocatalysts. BVMOs and FMOs are classified as Group B FPMOs,^12–15^ and even in cases of a low degree of sequence similarity, they share common structural features such as a Rossmann-like three-layer ββα sandwich domain for FAD binding, a NAD(P)H binding site, and a further ββα sandwich binding domain for the pyridine nucleotide.

BVMO and FMO enzymes have attracted a lot of attention from the scientific community because they provide safer, chemo- and stereoselective, and overall, more sustainable alternatives compared to the traditional methods for Baeyer–Villiger transformations and heteroatom oxidations, especially *S*- and *N*-oxides.^12,14^ BVMOs and FMOs have been used since the 1960s to catalyse a variety of oxidative transformations and several extensive reviews that summarise the use of these enzymes can be found in the literature.^16–20^ Depending on the nature of the substrate and the protonation state of peroxyflavin FAD-OO(H), BVMOs and FMOs can catalyse the electrophilic addition of oxygen onto sulfur or nitrogen substrates, leading to sulfoxides or *N*-oxide derivatives. BVMO-catalysed sulfoxidations make up most of the work reported in the literature, as these enzymes have traditionally been under scrutiny for their biocatalytic properties far more than FMOs. On the other hand, *N*-oxidations are more common with FMOs, as their physiological role is to metabolise nitrogen-containing toxins in organisms.^12–21^ Although the ability of BVMOs to perform enantioselective sulfoxidations has been proven throughout the decades, the full biocatalytic potential of FMOs for the synthesis of enantiopure sulfoxides remains largely unexplored. Additionally, most reports on the chiral production of sulfoxides focus on substrates that do not bear nitrogen functional groups that could potentially lead to the formation of *N*-oxide side products. Therefore, the lack of a detailed study on the chemoselectivity of BVMOs and FMOs when reacted with multifunctional substrates means that the applicability of these FPMOs remains still limited to structurally simple prochiral sulfides.

With the aim to expand the toolbox of biocatalysts and the substrate scope of green sulfoxidation reactions, herein we describe the identification of two new monooxygenase biocatalysts (BVMO and FMO) able to catalyse under mild conditions the enantiodivergent and selective oxidation of various sulfide substrates, including compounds bearing both a sulfur atom and an N-heteroaryl functional group. *In silico* studies have been also carried out to rationalise the enantioselectivity of these biotransformations (Fig. 1).

The Almac selectAZyme™ library, consisting of 50 BVMO and 60 FMO biocatalysts, either wild type or engineered freeze-dried cell free extracts (CFE), was initially screened on the pyridine-containing sulfide 4a, bearing a sulfide moiety and a potentially oxidisable heteroaryl nitrogen. The sulfide 4a (1.2 mM) was reacted with 10 g L^−1^ of the appropriate BVMO or FMO enzyme (cell free extract, CFE), 2.0 g L^−1^ glucose dehydrogenase (GDH), 5.5 eq. glucose and 2.0 mM NADP^+^ in 50 mM Tris-HCl buffer at pH = 8.0. The screening results are reported in Fig. 2 and Table S1.† The conversion of the sulfide into the desired sulfoxide, the enantioselectivity and the formation of sulfone over-oxidised side products were determined by chiral HPLC. All BVMO and FMO enzymes oxidised the sulfide 4a into the corresponding sulfoxide 5a. Biocatalyst BVMO145 led to enantiomer (*S*)-5a with excellent >99% ee and 75% conversion and it showed remarkable chemoselectivity for sulfide oxidation since no traces of the sulfone 6 and the *N*-oxide side products were observed.^22,23^ In contrast, BVMO129 and BVMO141 showed negligible stereoselectivity in the oxidation of 4a leading to the sulfone 6 as the major biotransformation product. Remarkably, biocatalyst FMO401 showed opposite enantioselectivity to BVMO145 providing the sulfoxide (*R*)-5a with excellent conversion (>99%) and good 65% ee. Interestingly, all FMO biocatalysts showed excellent activity on 4a leading to sulfoxide 5a with high conversion, but in most cases with low ee. No *N*-oxidation of the pyridine ring was observed for all the enzymes screened. Therefore, enzymes BVMO145 and FMO401 were selected for further substrate scope screening.

**Fig. 2:**
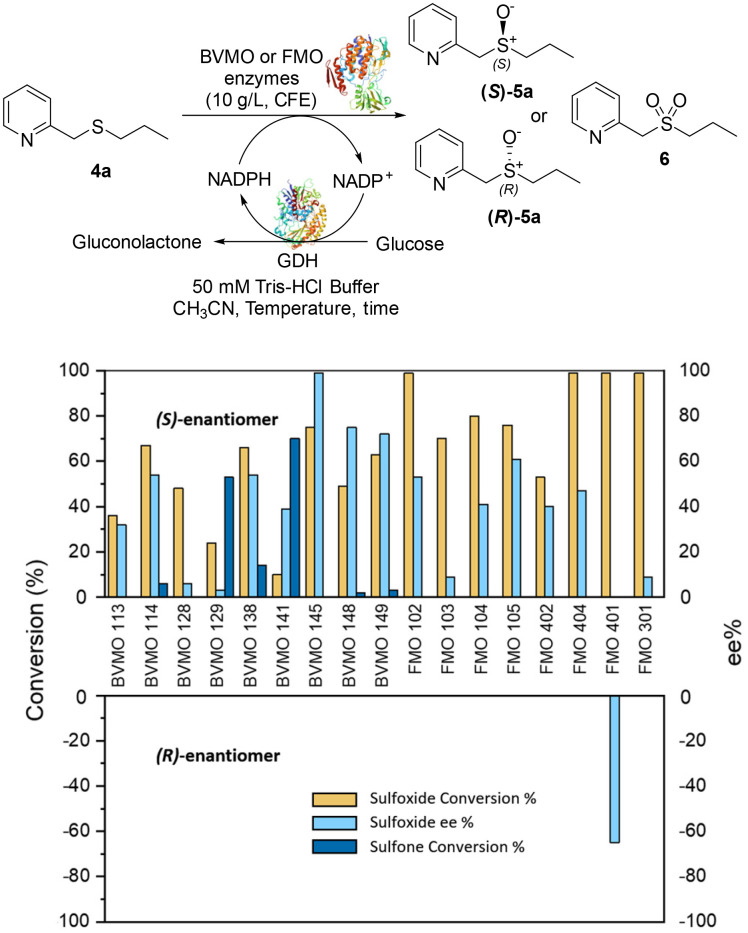
Screening results of BVMOs and FMOs for asymmetric oxidation of 4a to chiral sulfoxide 5a and the corresponding sulfone 6. Yellow bar: sulfoxide conversion % (determined by reversed phase HPLC using a Kromasil C18 column, monitored at 254 nm); sky-blue bar: ee % of 4a (determined by chiral HPLC using a Chiralpak IC column, monitored at 254 nm); navy bar: sulfone side product conversion % (determined by reversed phase HPLC using a Kromasil C18 column, monitored at 254 nm). For information about the enzyme sequence, contact Prof. Thomas S. Moody at Almac by email (see ESI, page S2†).

First, the optimization of the biotransformation conditions was conducted on substrate 4a with biocatalyst BVMO145. The results are reported in Table 1.

**Table 1:** Optimisation of BVMO145 catalysed sulfoxidation reaction conditions

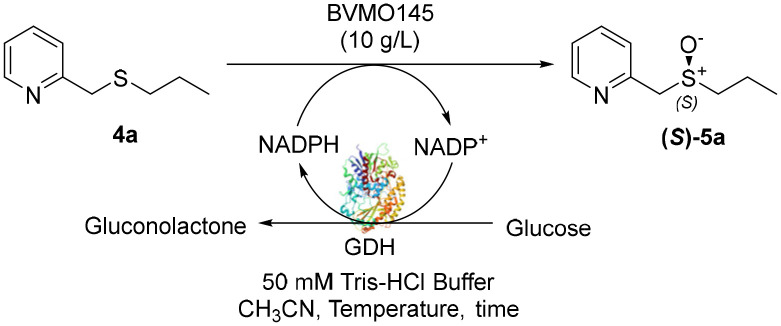								
Entry	Substrate 4a (mM)	NADP^+^ (mM)	GDH (g L^−1^)	pH	*T* (°C)	Time (h)	Sulfoxide (*S*)-5a conv.^a^ (%)	ee^b^ (%)
1	1.2	0.18	2	8.0	30	18	90	>99
2	1.2	0.12	2	8.0	30	18	90	>99
3	1.2	0.06	2	8.0	30	18	90	>99
4	1.2	0.06	5	8.0	30	18	40	>99
5	1.2	0.06	1	8.0	30	18	93	>99
6	5.0	0.06	1	8.0	30	18	42	>99
7	10	0.06	1	8.0	30	18	12	>99
8	20	0.06	1	8.0	30	18	12	>99
9	40	0.06	1	8.0	30	18	10	>99
10	5.0	0.25	1	8.0	30	18	93	>99
11	10	0.50	1	8.0	30	18	70	>99
12	5.0	0.25	1	7.0	30	18	93	>99
13	5.0	0.25	1	9.0	30	18	>99	>99
**14**	**5.0**	**0.25**	**1**	**9.0**	**37**	**18**	**>99**	**>99**
**15**	**5.0**	**0.25**	**1**	**9.0**	**37**	**4**	**>99**	**>99**
16	5.0	0.25	1	9.0	37	1	71	>99
17^c^	5.0	0.25	1	8.0	30	18	<1	n.d.^d^
18^e^	5.0	0.25	1	8.0	30	18	<1	n.d.^d^
19	—	0.25	1	8.0	30	18	—	—

a Determined by reversed phase HPLC using a Kromasil C18 column, monitored at 254 nm. Based on the consumption of the sulfide.

b Determined by chiral HPLC using a Chiralpak IC column, monitored at 254 nm.

c Empty Pet28a vector *E. coli* CFE. Additional blank experiments on different sulfide substrates with empty Pet28a vector *E. coli* CFE are reported in the ESI (Table S2).†

d n.d. = not determined.

e Enzyme-free reaction.

The cofactor NADPH was generated *in situ* by adopting a glucose dehydrogenase (GDH)/glucose system. The concentration of NADP^+^ was initially investigated and it was observed that lowering it to 0.06 mM (5 mol%) was sufficient for the reaction to take place without altering the product ee (>99%, Table 1, entry 3). The reduction of the GDH loading was then explored. When the GDH loading was reduced from 5.0 g L^−1^ (Table 1, entry 4) to 1.0 g L^−1^ (Table 1, entry 5), the conversion of 4a to sulfoxide (*S*)-5a improved significantly from 40% to 93%. The optimal concentration of 4a was also investigated to improve the efficiency of the method. Despite the excellent ee values, the increase of the concentration of 4a to 5–40 mM led to (*S*)-5a with low conversions (Table 1, entries 6–9), while increasing the concentration of NADP^+^ to 0.25–0.5 mM allowed the increase of the concentration of 4a to 5–10 mM maintaining respectively excellent ee values (99%) and high conversions (Table 1, entries 10 and 11). Finally, the effects of pH and temperature on the biotransformation were investigated.

An increase of the pH from 8.0 to 9.0 led to a remarkable improvement of the conversion (>99%, Table 1, entries 12 and 13), while no differences were observed when the reaction was run at higher temperatures (Table 1, entry 14). However, carrying out the sulfoxidation at 37 °C allowed the reduction of the biotransformation time to 4 h (Table 1, entry 15) and the setting of the optimal reaction conditions. Finally, three control experiments were performed with empty pET28a vector *E. coli* CFE, enzyme-free and substrate 4a free reactions to confirm the oxidizing activity of the enzymes (Table 1, entries 17–19). In all cases, no product (*S*)-5a was detected confirming the catalytic role of the enzyme in the biotransformation. A frequent issue reported in BVMO catalytic systems is that the low reactivity of the substrates can cause the spontaneous decomposition of peroxyflavin, with the release of hydrogen peroxide that can be responsible for side reactions, like *N*-oxidations. Remarkably, under the optimised conditions, no formation of side products (pyridine *N*-oxide) or decreased ee of (*S*)-5a was observed.^24^

With the optimal reaction conditions in hand, the scope of the BVMO145 and FMO401 enzymatic sulfoxidation was investigated (Table 2). Sulfides 4a–c were converted in 18–24 h by BVMO145 into the corresponding (*S*)-sulfoxides (*S*)-5a–c with excellent ee values (>99%) and good to high conversions (Table 2, entries 1, 6 and 8). The biocatalyst FMO401 showed complementary enantioselectivity to BVMO145, affording enantiomers (*R*)-5a and b with high conversions and good to high ee values (Table 2, entries 2 and 7). Remarkably, FMO401 converted 4c into (*R*)-5c with high ee (80%) and yield (93%), (Table 2, entry 9). The oxidation of substrates 4a and 4c with both biocatalysts proved to be selective towards the formation of the sulfoxide products 5a and 5c, while no formation of the sulfone side products 6a and 6c, arising from overoxidation of 5, was observed. On the other hand, the enzymatic oxidation of the smaller sulfide 4b led to the formation of 6b in a variable amount when either BVMO145 or FMO401 was used (Table 2, entries 6 and 7).

**Table 2:** Substrate scope of flavoenzyme catalysed sulfoxidation

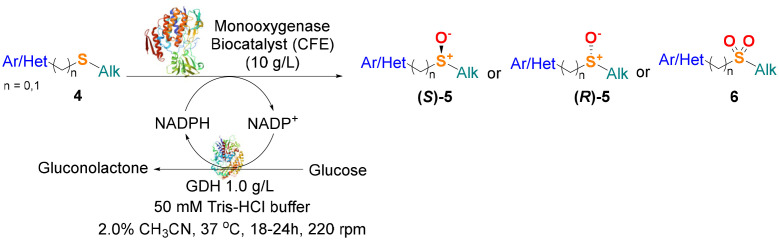							
Entry	Sulfide 4 substrate		Biocatalyst	Sulfoxide 5 enantiomer	Sulfoxide 5 ee^a^ (%)	Sulfoxide 5 yield^b^ (%)	Sulfone 6 conv.^c^ (%)
1	4a	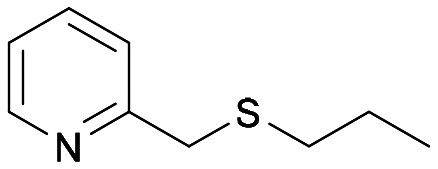	BVMO145	(*S*)-5a	>99	70 (99)^c^	0
2			FMO401	(*R*)-5a	64	72	0
3			BVMO145 W506A	(*R,S*)-5a	Racemic	3	0
4			TmCHMO	(*S*)-5a	>99	(40)^c^	60
5			PAMO	(*S*)-5a	84	53	32

6	4b	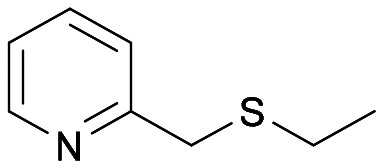	BVMO145	(*S*)-5b	>99	(65)^c^	35
7			FMO401	(*R*)-5b	60	30	26

8	4c	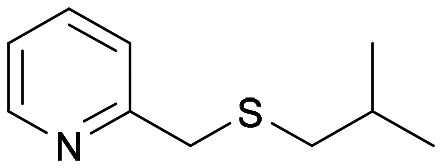	BVMO145	(*S*)-5c	>99	31	0
9			FMO401	(*R*)-5c	80	93	0

10	4d	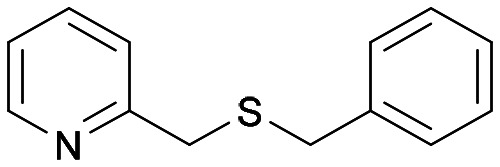	BVMO145	(*S*)-5d	n.d.^d^	<1	0
11			FMO401	(*R*)-5d	n.d.^d^	<1	0

12	4e	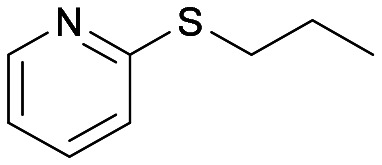	BVMO145	(*S*)-5e	n.d.^d^	6	0
13			FMO401	(*R*)-5e	46	67	0

14	4f	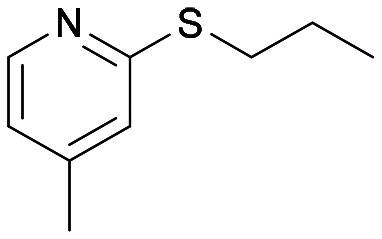	BVMO145	(*S*)-5f	n.d.^d^	<1	0
15			FMO401	(*R*)-5f	<1	57	0

16	4g	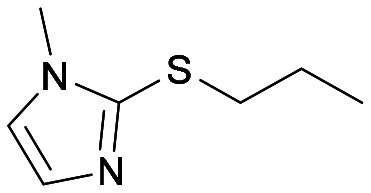	BVMO145	(*S*)-5g	76	57	0
17			FMO401	(*R*)-5g	<1	92	0

18	4h^e^	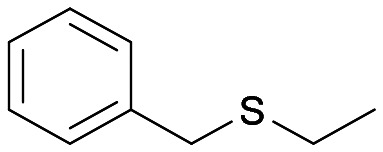	BVMO145	(*S*)-5h	>99	61	0
19			FMO401	(*R*)-5h	51	45	0
20			BVMO145 W506A	(*S*)-5h	n.d.^d^	<1	0
21			TmCHMO	n.d.^d^	n.d.^d^	<1	>99
22			PAMO	(*S*)-5h	>99	48	4

23	4i	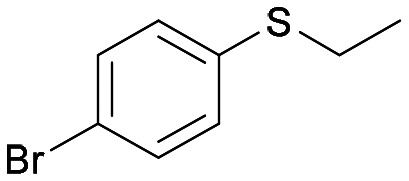	BVMO145	(*S*)-5i	>99	24	0
24			FMO401	(*S*)-5i	14	50	0

25	4j^e^	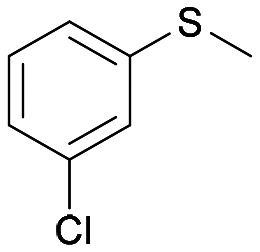	BVMO145	(*S*)-5j	>99	24	0

26	4k	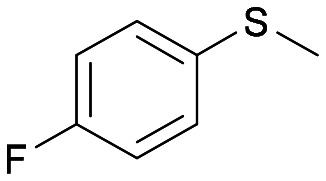	BVMO145	(*S*)-5k	>99	24	0
27			FMO401	(*S*)-5k	>99	22	0

28	4l	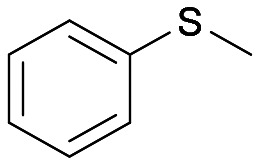	BVMO145	(*S*)-5l	>99	76	0

29	4m^e^	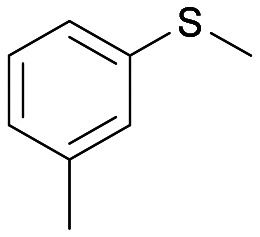	BVMO145	(*R*)-5m	>99	24	0
30			BVMO145 W506A	(*R*)-5m	n.d.^d^	<1	0
31			TmCHMO	n.d.^d^	n.d.^d^	<1	>99
32			PAMO	(*S*)-5m	22	52	4

33	4n	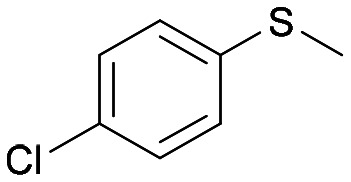	BVMO145	(*S*)-5n	n.d.^d^	<1	0

34	4o	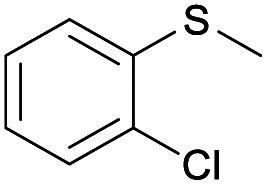	BVMO145	(*S*)-5o	n.d.^d^	<1	0

a Determined by chiral HPLC using a Chiralpak IG, IC or OD-H column, monitored at 254 nm. The absolute configuration was determined by comparing to the literature.

b HPLC yield. Calculated using a reversed phase Agilent Eclipse Plus C18 column, monitored at 254 nm and methyl phenyl sulfoxide as an internal standard unless stated otherwise.

c HPLC % conversion calculated from normal phase HPLC using a Chiralpak IG, IC or OD-H column.

d n.d. = not determined.

e Blank experiment with empty pET28a vector *E. coli* CFE is reported in the ESI (Table S2).†

The efficacy of BVMO145 was then compared with that of the biocatalysts TmCHMO and PAMO,^25,26^ previously reported in the literature for sulfoxidation reactions (Table 2, entries 4 and 5). Under the same reaction conditions, both TmCHMO and PAMO afforded the sulfoxide (*S*)-5a with high ee values and good conversions, even though slightly lower than those of BVMO145. However, a significant amount of the side product sulfone 6a was obtained from these biotransformations (60% and 32% conversion respectively). The green metrics for the preparative biocatalytic sulfoxidation of the sulfides 4a and 4c are reported in Table 3.

**Table 3:** Green metrics for the biocatalytic sulfoxidation of sulfides 4a and 4c

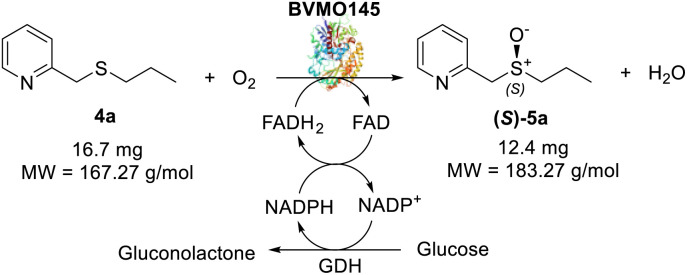			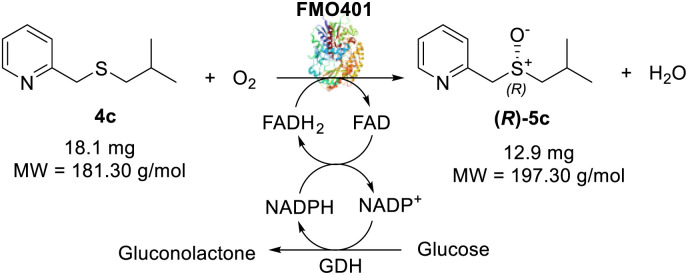		
	MW (g mol^−1^)	Mass (mg)		MW (g mol^−1^)	Mass (mg)
Sulfide 4a	167.27	16.7	Sulfide 4c	181.3	18.1
Sulfoxide 5a	183.27	12.4	Sulfoxide 5c	197.3	12.9
O_2_	32.00	—	O_2_	32.00	—
CH_3_CN	41.05	314	CH_3_CN	41.05	314
NADP^+^	744.4	3.7	NADP^+^	744.4	3.7
Glucose	180	180	Glucose	180	180
Tris	121	118.5	Tris	121	118.5
Yield % = 68%^a^			Yield % = 65%^a^		
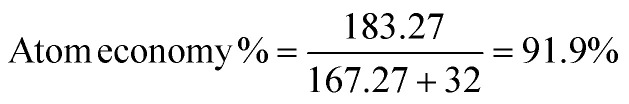			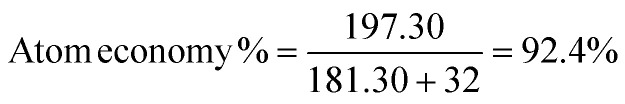		
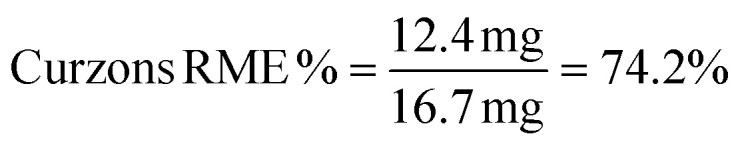			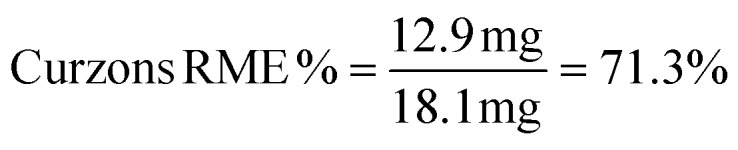		
Kernel RME = 0.68 × 0.919 = 0.624			Kernel RME = 0.65 × 0.924 = 0.6		
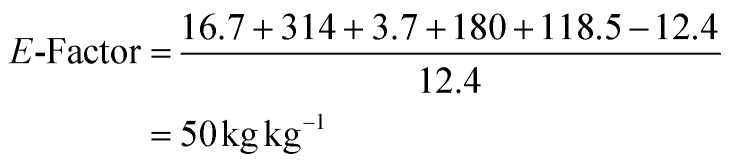			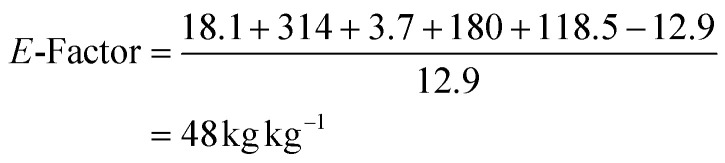		

a Isolated yield after purification on silica gel (see the ESI† for preparative procedures).

The sulfide 4d bearing a bulky benzyl group on the sulfur atom was not accepted by either BVMO145 or FMO401 (Table 2, entries 10 and 11), while the sulfides 4e and 4f bearing a pyridine directly on the sulfur atom proved to be poor substrates for both catalysts, with the exception of 4e that was converted by FMO401 into (*R*)-5e with 46% ee (Table 2, entries 12–15). Such data clearly suggest that a methylene spacer between the pyridine ring and the sulfur atom is required by the enzyme to carry out the sulfoxidation reaction. On the other hand, the sulfide 4g bearing an imidazole moiety was converted into (*S*)-5g in good yields and good ee by BVMO145, while in the presence of FMO401, (*R*)-5g was obtained with an excellent HPLC yield (92%), even if as a racemate (Table 2, entries 16 and 17). Again, no traces of sulfone 6 side products were observed in all the transformations catalysed by both BVMO145 and FMO401.

Molecular docking and molecular dynamics simulations were then carried out with selected substrates to interpret the substrate specificity and enantioselectivity displayed by the enzymes. A first computational analysis of the biocatalysts revealed that the substrate binding pockets of BVMO145 and FMO401 are considerably different. According to the accepted monooxygenase mechanism of action (Fig. 3a),^27,12^ one of the electron lone pairs of the sulfur in substrates 4 attacks the peroxide group of C4a-hydroperoxyflavin and it is converted into the corresponding sulfoxide products 5.

**Fig. 3:**
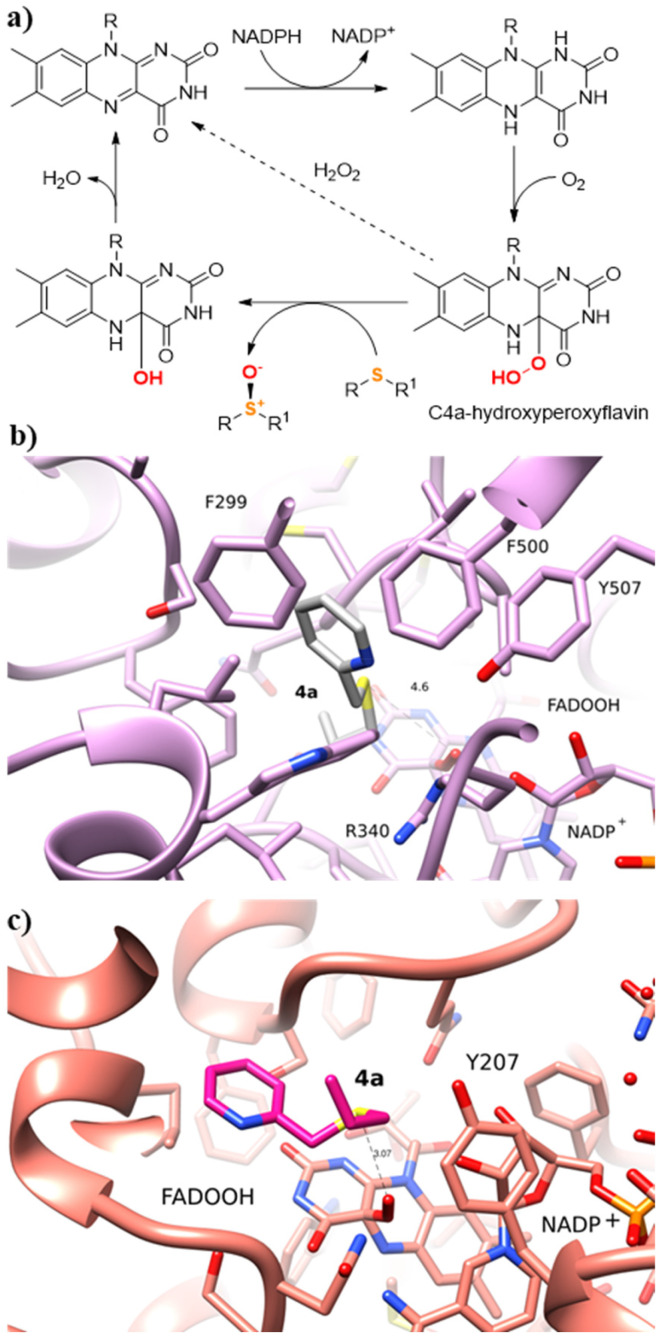
(a) Mechanism of sulfoxidation by monooxygenase biocatalysts. (b) Sulfoxidation of 4a by BVMO145. The active site of BVMO-145 only allows the binding of the substrate 4a to the left side of C4a-hydroperoxyflavin. (c) Sulfoxidation of 4a by FMO401. The hydroperoxyflavin is orientated to the sulfur lone electron pair pointing below giving the (*R*)-product. All hydrogens were omitted for clarity.

The BVMO145 active site is narrow with a well-documented H-bond between the catalytic arginine R340 and C4a-hydroperoxyflavin. Due to the steric hindrance of hydrophobic residues and NADP^+^, BVMO145 allows substrate 4a to only be oriented from the left side of C4a-hydroxyperoxyflavin, with the pro-(*S*) lone electron pair of the sulfur reacting with the oxygen of the hydroperoxide, leading to the formation of the enantiomer (*S*)-5a with excellent ee (>99%) (Fig. 3b). The W506 of BVMO145 seems to play a key role in the sulfoxidation of 4a, since its replacement with an alanine (W506A mutation) led to the loss of catalytic activity and stereoselectivity (Table 2, entry 3). The W506 is located in the substrate tunnel and forms a hydrogen bond with the O2′ oxygen of the ribose moiety of the NADPH cofactor. Other residues within the substrate tunnel that interact with the pyridine moiety of the substrate 4a are F500 and F299. In contrast to BVMO145, the FMO401 binding pocket is wider, and the propyl moiety of the substrate 4a moves closer to the hydroperoxyl group, reacting *via* the pro-(*R*) lone electron pair of the sulfur and forming the (*R*)-5a sulfoxide product (Fig. 3c).

In FMO401, the substrate tunnel residue Y207 forms a H bond with the NADP^+^/NADPH cofactor. Mutations of this tyrosine residue to valine or alanine (respectively the biocatalysts FMO402 and FMO404) have a great effect on the activity and selectivity of the enzyme, even if to a lesser extent than the mutation W507A in BVMO145. In fact, both mutants FMO402 and FMO404 provide the sulfoxide (*S*)-5a with good to high conversion compared to FMO401, but with lower enantioselectivity.

The computational analysis of the bulkier sulfide 4d bearing a benzyl substituent on the sulfur atom revealed that 4d is not able to fit the BVMO145 substrate binding tunnel near the isoalloxazine ring and thus to be oxidised into sulfoxide 5d (Fig. S1†). On the other hand, the sulfide 4e bearing a pyridine ring directly bonded to the sulfur atom is able to approach closely and bind the hydroperoxyl group in FMO401 affording (*R*)-5e with good 67% HPLC yield (Fig. 4b). However, in BVMO145, the pyridine ring of 4e forms a charge-π interaction with the catalytic arginine residue leaving the sulfur atom too far from the C4a-hydroperoxyl group to allow a nucleophilic attack and, in turn, the formation of (*S*)-5e (Fig. 4a).

**Fig. 4:**
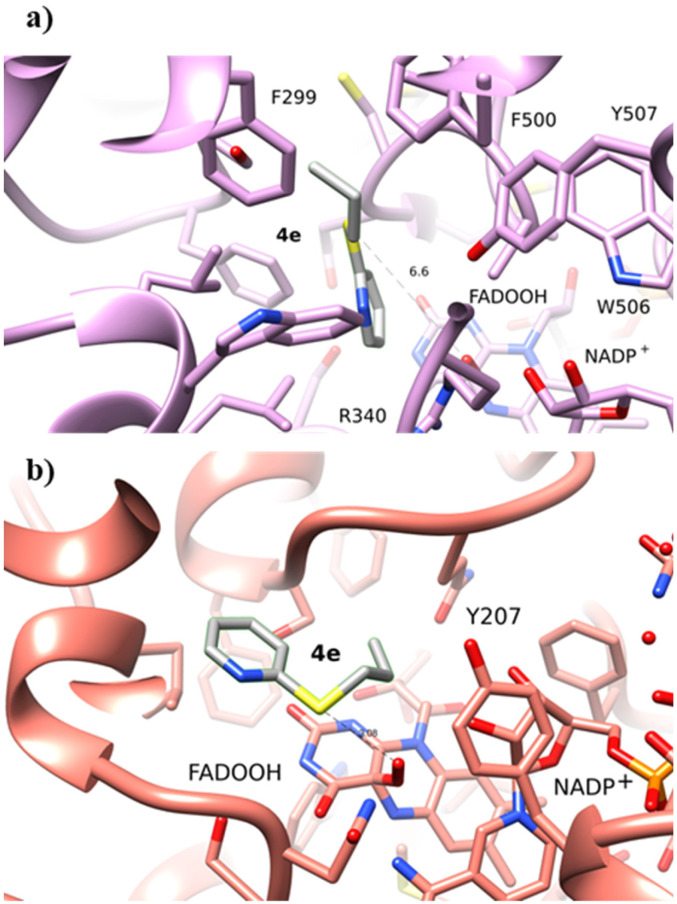
(a) Sulfoxidation of 4e by BVMO145. The orientation of 4e is not correct to allow the sulfur nucleophilic attack on the hydroperoxyl group. (b) Sulfoxidation of 4e by FMO401. The sulfide substrate 4e is in the correct position to allow sulfoxide 5e formation.

Intrigued by the opposite stereoselectivity of the BVMO145 and FMO401 biocatalysts, the enantioselective sulfoxidation of a series of aryl-alkyl sulfides 4h–o was also investigated (Table 2). The biocatalyst BVMO145 retains activity and excellent enantioselectivity on most sulfide substrates affording the (*S*)-5h–l enantiomers with excellent enantiopurity (>99% ee) and good conversions (Table 2, entries 23, 25, 26 and 28). Interestingly, the oxidation of 4h with PAMO showed opposite enantioselectivity to BVMO145, leading to the enantiomer (*R*)-5h with excellent enantioselectivity (>99%) and good conversion (Table 2, entry 22), while the oxidation of 4h with TmCHMO afforded the side product sulfone 6h as the only reaction product (Table 2, entry 21). The BVMO mutant W506A proved to be inactive also on 4h, thus highlighting the key role of the W506 residue on the activity. Surprisingly, the sulfide 4m bearing a *m*-methyl-substituent on the aromatic ring was oxidized by BVMO145 into the sulfoxide enantiomer (*R*)-5m (Table 2, entry 29). *In silico* studies showed that the methyl-phenyl ring of sulfide 4m forms a charge-π interaction with the catalytic arginine residue R340 directing the approach of the substrate to the hydroperoxyl group to form the (*R*)-5m sulfoxide, albeit with a low yield as in the case of substrate 4e (Fig. 5a and 4a).

**Fig. 5:**
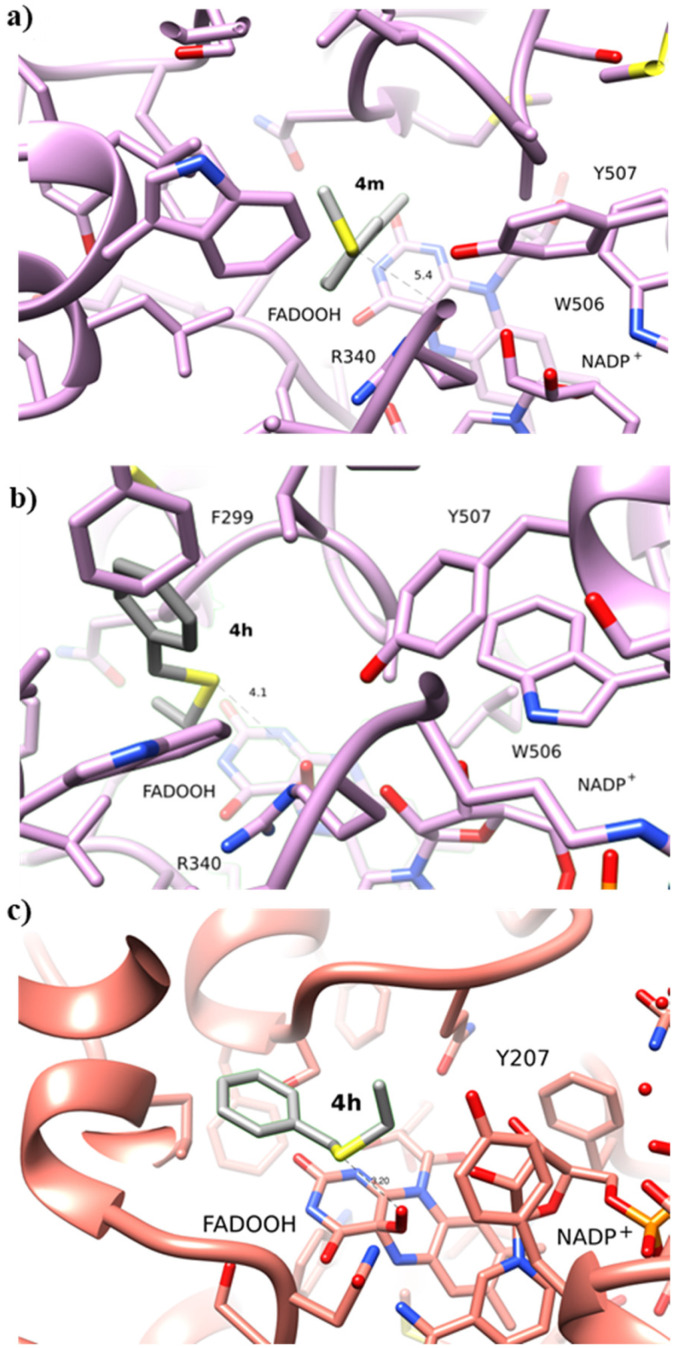
(a) Binding of 4m to BVMO145. The substrate 4m forms a charge-π interaction with the catalytic R340; (b) binding of 4h to BVMO145; (c) binding of 4h to FMO401. The substrate 4h binds with a similar position to substrate 4a.

Interestingly, PAMO showed again opposite enantioselectivity to BVMO145, affording (*S*)-5m, even if with a moderate yield and ee. The biocatalyst FMO401 showed complementary enantioselectivity on substrates 4h and 4i which were converted into the corresponding (*R*)-5h and 5i sulfoxides with good yields but low enantioselectivity. While FMO401 showed excellent enantiopreference in the oxidation of pyridine-containing sulfides, lower selectivity was observed with smaller thio-phenyl substrates.

Interestingly, sulfide 4k was oxidised by FMO401 into the corresponding sulfoxide (*S*)-5k showing an opposite selectivity compared to other substrates (Table 2, entry 27). *In silico* studies clearly show the different enantiopreference of the biocatalysts BVMO145 and FMO401 on substrate 4h through opposite interaction with C4a-hydroxyperoxyflavin (Fig. 5b and c).

## Conclusions

Two new biocatalysts BVMO145 and FMO401 have been discovered in this work and they have been successfully used in the sulfoxidation of a variety of sulfide substrates. The two biocatalysts showed high enantio- and regio-selectivity in the oxidation of sulfide substrates bearing a pyridine ring, leading to the desired products in high ee (up to 99%) without the formation of sulfone or *N*-oxide side products. Remarkably, the two enzymes showed opposite enantiopreference, with the enzyme BVMO145 affording the sulfoxide (*S*)-enantiomers and FMO401 catalysing the formation of the sulfoxide (*R*)-enantiomers. *In silico* studies allowed the rationalisation of the opposite enantioselectivity and substrate preference of the two biocatalysts. In conclusion, the identification of BVMO145 and FMO401 allows the expansion of the biocatalysis toolbox of oxidative monooxygenase biocatalysts to access enantiomerically pure sulfoxides under mild, selective and green conditions.

## Author contributions

DC and TSM conceived and directed the project. JW and SA carried out the biocatalytic and synthetic experimental work. ATPC and DQ carried out and managed the computational studies. JC managed and carried out the biology work and prepared the biocatalysts.

## Data availability

The data supporting this article have been included as part of the ESI.†

## Conflicts of interest

There are no conflicts to declare.

## Supplementary Material

GC-026-D4GC02657H-s001
