# Supplementary material for: Expanding the toolbox of Baeyer–Villiger and flavin monooxygenase biocatalysts for the enantiodivergent green synthesis of sulfoxides
Source: Green Chem. 2024 Jul 5;26(15):8685–93. doi: 10.1039/d4gc02657h (PMC11284623; doi:10.1039/d4gc02657h)
Supplement: GC-026-D4GC02657H-s001 [file GC-026-D4GC02657H-s001.pdf]

Electronic Supplementary Material (ESI) for Green Chemistry.
This journal is © The Royal Society of Chemistry 2024

Expanding the toolbox of Baeyer Villiger and flavin monooxygenase biocatalysts for the enantiodivergent green synthesis of sulfoxides.

Jingyue Wu,[a] Silvia Anselmi,[a] Alexandra T.P. Carvalho,[b] Jill Caswell,[b] Derek J. Quinn,[b] Thomas S. Moody[b,c,*] and Daniele Castagnolo[a,*]

[a]Department of Chemistry, University College London, 20 Gordon Street, WC1H 0AJ, London, United Kingdom. [b]Department of Biocatalysis & Isotope Chemistry, Almac, 20 Seagoe Industrial Estate, Craigavon, BT63 5QD, United Kingdom. [c]Arran Chemical Company Limited, Unit 1 Monksland Industrial Estate, Athlone, Co. Roscommon, Ireland.

Supporting information

Table of Content

1. General	S1
2. Main protein sequences used in this work	S1
3. Expression of BVMOs and FMOs	S1
4. Molecular Docking	S2
5. Molecular Dynamics Simulations	S2
6. Screening of BVMOs and FMOs	S4
7. General procedure of enzymatic sulfoxidation using BVMO 145 or FMO D9	S5
8. Procedure for preparative enzymatic sulfoxidation of **4a** with BVMO 145	S5
9. Procedure for preparative enzymatic sulfoxidation of **4c** with FMO401	S5
10. General procedure for the synthesis of sulfides **4a-f** and **4h**	S5
11. General procedure for the synthesis of racemic sulfoxides **5a-k**, **5n** and **5o**	S8
12. General procedure for the synthesis of sulfones **6**	S12
13. Blank Experiments	S13
14. Copies of NMR spectra	S14
15. Copies of HRMS spectra for new compounds	S42
16. HPLC analysis and traces	S46
17. References	S61

1. General

Reagents and solvents were used as supplied from the vendor without further purification. Thin layer chromatography plates (Merk, silica gel 60 F254, aluminium backed) were viewed under UV light. $MgSO_4$ (Sigma Aldrich, anhydrous ≥ 98.0 %) was used as the drying agent. Column chromatography was performed on silica gel for flash chromatography (Sigma Aldrich, 40-63 µm particle size, 60 Å pore size). Microwave irradiations were conducted using a CEM Discover Synthesis Unit. Products were characterised by ^{1}H NMR, ^{13}C NMR and ^{19}F NMR spectra where applicable obtained from one of the following: a) Bruker (Germany) Ascend400 Spectrometer (dH 400 MHz, dC 101 MHz, dF 376 MHz) at 300 K; b) Bruker (Germany) Avance III 400 (dH 400 MHz, dC 101 MHz) at 300 K; c) Bruker (Germany) Avance Neo 500 (dH 500 MHz, dC 126 MHz) at 300 K. Chemical shifts are reported in ppm relative to the reference peaks of the solvents: $CDCl_3$ (1H NMR 7.26 and 13C NMR 77.16) unless stated otherwise. Coupling constants (J) are reported in Hz, multiplicities are specified as singlet (s), doublet (d), triplet (t), doublet of doublet (dd), doublet of quartet (dq), doublet of doublet of doublet (ddd), triplet of doublet (td), multiplet (m). Chiral HPLC analysis was carried out using one of the following: a) Agilent series 1100 LC system coupled with UV detector at 254 nm; b) Agilent series 1250 UPLC system coupled with UV detector at 254 nm. The columns used were Chiralpak IC® (0.5µm, 4.6mm X 250mm), Chiralpak IG® (5µm, 4.6mm X 250mm), Chiralpak ID® (0.5µm, 4.6mm X 250mm), and Chiracel OD-H (0.5µm, 4.6mm X 250mm) supplied by Daicel. Hexane, isopropanol (IPA) and ethanol (EtOH) were used as an isocratic mobile phase system for all columns. Normal phase HPLC analysis was carried out using Agilent Eclipse Plus C18 column and Kromasil C18 column. H2O and ACN were used as mobile phase components. Mass spectra were acquired in positive mode scanning over the mass range of 50 – 1500. The following ion source parameters were used: drying gas flow, 12 mL/min; nebulize pressure, 35 psi; and drying gas temperature, 350 ºC.

2. Main protein sequences used in this work

For information about enzyme sequence, please contact Prof. Thomas S. Moody at Almac, tom.moody@almacgroup.com.

3. Expression of BVMOs and FMOs

Plasmids containing the BVMO and FMO genes cloned into pET28(a) were transformed into *E.coli* expression strain BL21 (DE3). 2 µL of the BVMO/FMO plasmid was added to 8 µL of *E.coli* BL21 (DE3) cells and incubated on ice for 30 mins. Following incubation the cells were heat shocked at 42 °C for 30 secs and then back on ice for 2 mins. 200 µL of SOC media was added and incubated for 1 hour at 37 °C. After the incubation, the cells were plated on to agar plates containing LB agar supplemented with X µg/mL of kanamycin and incubated overnight at 37 °C or until colonies are formed.

For protein expression, a primary culture was prepared by selecting a single colony from the agar plate to inoculate 10 mL of LB broth with kanamycin. The primary culture was incubated overnight at 37 °C with shaking at 200 rpm and then the primary culture was used to inoculate 1 L of LB broth in a 2 L shake flask. The shake flask was again incubated at 37 °C until an OD_{600nm} of 0.6-0.8 is obtained and then temperature was reduced to 25 °C. Protein expression was induced by the addition of 0.1 mM IPTG to the shake flask followed by overnight incubation at induction temperature of 25 °C. The following day the culture was harvested by centrifugation at 4000rpm for 15 mins. The supernatant was discarded and the cell pellet was disrupted by sonication (10 sec on, 10 sec off, 6 cycles). The lysate was clarified by centrifugation and supernatant retained for lyophilisation.

4. Molecular Docking

Models of the enzymes understudy were made resorting to the template assisted colabfold program.[1] The C4a-peroxyflavin was modelled. The peroxo electrophilic oxygen was considered the center of the grid-box for molecular docking. All substrates were geometry optimized and charges calculated according to the RESP method.

Molecular docking was performed using Autodock4.2[2] with the Lamarckian genetic algorithm (LGA) using a grid. A total of 1000 LGA runs were carried out per system. The population was 300, the GA elitism=1, the maximum number of generations was 27000 and the maximum number of energy evaluations was 2500000.

5. Molecular Dynamics Simulations

Molecular dynamics (MD) simulations of the enzyme:sulfide complexes were run to further assess the sulfides binding to the enzymes. The simulations were performed with the amber parm99SB[3] force field. Energy minimization was followed by equilibration to slowly heat the system from 0 to 310 K. Explicit solvent and Periodic boundary conditions (PBC) were used. For each enzyme 25 ns of production simulations were carried. The time step was set to 2 fs and constraints were applied to all bonds involving hydrogen atoms. The particle mesh Ewald (PME) method[4] was used to calculate electrostatic interactions.

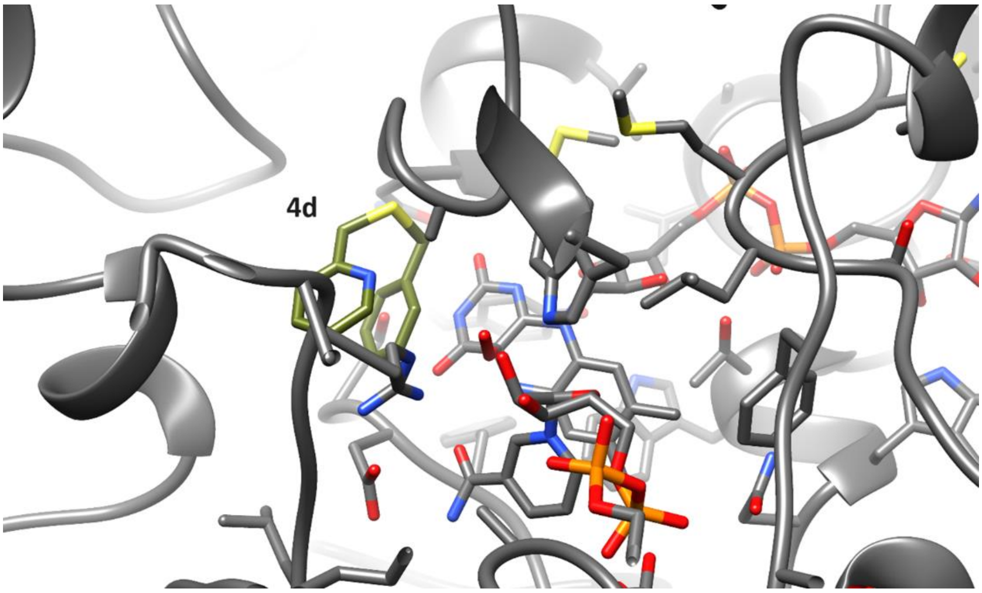

Figure S1. Binding of sulfide **4d** to BVMO145

6. Screening of BVMOs and FMOs

20 µL of 10 mg/mL stock solution in CH_3CN of the substrate **1a** (0.2 g/L final concentration), was added to 980 µL 50 mM Tris-HCl buffer pH 8.0 containing $NADP^+$ (1.5 mg), GDH (2 mg), glucose (3 mg) and 8.4 mg BVMO or FMO to initiate the reaction. The reaction was shaken at 30 °C for 18-24 h. Upon completion, the reaction was extracted by EtOAc (250 µL, 3 times). EtOAc fraction was dried over $MgSO_4$ and evaporated under vacuum, then resuspended to EtOH and analysed by normal phase HPLC using Chiralpak IC column to determine the enantiomeric excess and conversion.

Table S1. Screening of BVMO and FMO enzymes from Almac library

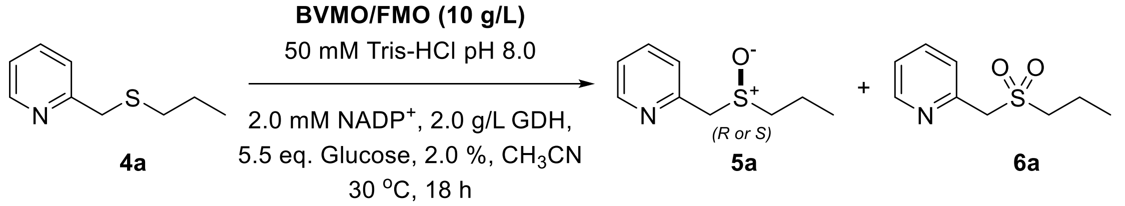

Entry	Enzyme	Code	Sulfoxide 5a conv. (%)[a]	Sulfone 6a conv. (%)[a]	Sulfoxide 5a ee (%)[b]	Enantiomer
1	BVMO	113	36	-	32	(*S*)
2	BVMO	114	67	6	54	(*S*)
3	BVMO	128	48	-	6	(*S*)
4	BVMO	129	24	53	3	(*S*)
5	BVMO	138	66	14	54	(*S*)
6	BVMO	141	10	70	39	(*S*)
**7**	**BVMO**	**145**	**75**	**-**	**>99**	**(*S*)**
8	BVMO	148	49	2	75	(*S*)
9	BVMO	149	63	3	72	(*S*)
10	FMO	102	99	n.d.[c]	53	(*S*)
11	FMO	103	70	n.d.[c]	9	(*S*)
12	FMO	104	80	n.d.[c]	41	(*S*)
13	FMO	105	76	n.d.[c]	61	(*S*)
14	FMO	402	53	n.d.[c]	40	(*S*)
15	FMO	404	99	n.d.[c]	47	(*S*)
**16**	**FMO**	**401**	**99**	**n.d.[c]**	**65**	**(*R*)**
17	FMO	301	99	n.d.[c]	9	(*S*)

[a]Determined by reversed phase HPLC using a Kromasil C18 column, monitored at 254 nm. [b]Determined by chiral HPLC using Chiralpak IC column, monitored at 254 nm. [c]n.d. = not determined.

7. General procedure of enzymatic sulfoxidation using BVMO 145 or FMO D9

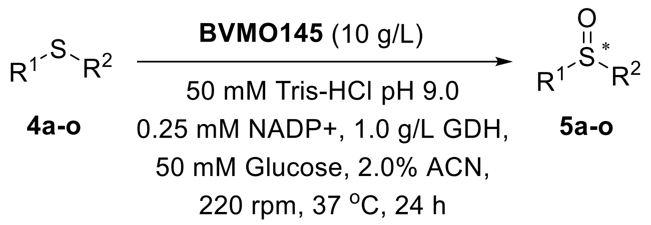

14 µL of a 250 mM stock solution in CH_3CN of the relevant sulfide substrate **4a-o** (5.0 mM final concentration), was added to 686 µL 50 mM Tris-HCl buffer pH 9.0 containing $NADP^+$ (0.25 mM, 0.05 eq.), GDH (1.0 g/L), glucose (50 mM, 10 eq.) and appropriate enzyme (10 g/L) to initiate the reaction. The reaction was shaken at 37 °C for 24 h. Upon completion, a 70 µL aliquot was extracted with EtOAc (3x50 µL), centrifuged (12500 rpm, 10 min) and the collected organic layers were analysed by normal phase HPLC using chiral columns to determine the enantiomeric excess (see HPLC analysis and traces for conditions). The remaining reaction mixture was quenched with 700 µL of CH_3CN and 12.6 µL of 250 mM internal standard in CH_3CN was added into the reaction mixture. The conversion was calculated by reversed phase HPLC using Agilent Eclipse Plus C18 column.

8. Procedure for preparative enzymatic sulfoxidation of 4a with BVMO 145

400 µL of a 250 mM stock solution in CH_3CN of the relevant sulfide substrate **4a** (5.0 mM final concentration), was added to 19.6 mL 50 mM Tris-HCl buffer pH 9.0 containing $NADP^+$ (0.25 mM, 0.05 eq.), GDH (1.0 g/L), glucose (50 mM, 10 eq.) and BVMO145 (10 g/L) to initiate the reaction. The reaction was shaken at 37 °C for 24 h. The reaction mixture was extracted with EtOAc (8x5 mL) after completion. Organic layer was collected and dried over $MgSO_4$ and evaporated under vacuum. Crude product were purified by flash column chromatography using an appropriate eluent mixture of DCM and MeOH to afford the resulting 12.4 mg pure enantioenriched sulfoxide (68% isolated yield).

9. Procedure for preparative enzymatic sulfoxidation of 4c with FMO401

400 µL of a 250 mM stock solution in CH_3CN of the relevant sulfide substrate **4c** (5.0 mM final concentration), was added to 19.6 mL 50 mM Tris-HCl buffer pH 9.0 containing $NADP^+$ (0.25 mM, 0.05 eq.), GDH (1.0 g/L), glucose (50 mM, 10 eq.) and FMO401 (10 g/L) to initiate the reaction. The reaction was shaken at 37 °C for 24 h. The reaction mixture was extracted with EtOAc (8x5 mL) after completion. Organic layer was collected and dried over $MgSO_4$ and evaporated under vacuum. Crude product were purified by flash column chromatography using an appropriate eluent mixture of DCM and MeOH to afford the resulting 12.9 mg pure enantioenriched sulfoxide (65% isolated yield).

10. General procedure for the synthesis of sulfides 4a-f and 4h

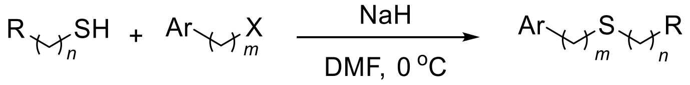

The appropriate thiol (3.0 mmol) and NaH (6.0 mmol) were stirred in anhydrous DMF under N_2 for 20 min in an ice bath. Then, an appropriate alkyl halide (3.6 mmol) was added to the mixture and the reaction was stirred until completion was observed on TLC. The reaction was quenched with water, extracted with EtOAc and the combined organic layers were washed with 10 folds of H_2O to ensure the full removal of DMF from the crude. The collected organic layer was dried over $MgSO_4$ and evaporated

under vacuum. Crude sulfides were purified by flash column chromatography using an appropriate eluent mixture of EtOAc and hexane to afford the resulting pure sulfide.

2-((Propylthio)methyl)pyridine (4a)

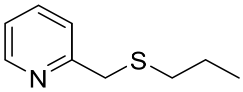

Synthesised from 2-(bromomethyl)pyridine hydrobromide and propanethiol. Pale yellow oil (62% yield). v_{max}/cm^{-1}: 2960, 1590, 1433. ^{1}H NMR (400 MHz, $CDCl_3$) δ 8.44 (d, *J* = 4.1 Hz, 1H), 7.63 – 7.52 (m, 1H), 7.30 (d, *J* = 7.9 Hz, 1H), 7.07 (ddd, *J* = 7.6, 4.9, 1.2 Hz, 1H), 3.75 (s, 2H), 2.43 – 2.35 (m, 2H), 1.58 – 1.42 (m, 2H), 0.87 (t, *J* = 7.3 Hz, 3H) ppm. ^{13}C NMR (101 MHz, $CDCl_3$) δ 159.00, 149.05, 136.61, 122.96, 121.74, 38.05, 33.68, 22.52, 13.38 ppm. HRMS (ESI) m/z calcd. for $C_9H_{14}ON^{32}S^+$ $[M+H]^+$ 168.08415; found 168.0840.

2-((Ethylthio)methyl)pyridine (4b)[5]

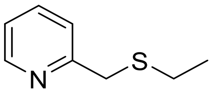

Synthesised from 2-(bromomethyl)pyridine hydrobromide and ethanethiol. Orange oil (642 mg, 64% yield). v_{max}/cm^{-1}: 2925, 1590, 1433. ^{1}H NMR (400 MHz, $CDCl_3$) δ 8.52 (ddd, *J* = 5.0, 1.9, 0.9 Hz, 1H), 7.71 – 7.62 (m, 1H), 7.42 – 7.35 (m, 1H), 7.16 (ddd, *J* = 7.5, 4.9, 1.2 Hz, 1H), 3.86 (s, 2H), 2.51 (q, *J* = 7.4 Hz, 2H), 1.23 (t, *J* = 7.4 Hz, 3H) ppm. ^{13}C NMR (101 MHz, $CDCl_3$) δ 159.06, 149.02, 137.07, 123.26, 122.02, 37.78, 25.79, 14.56 ppm.

2-((Isobutylthio)methyl)pyridine (4c)

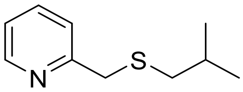

Synthesised from 2-(bromomethyl)pyridine hydrobromide and 1-bromo-2-methylpropane. Yellow oil (512.9 mg, 94% yield). ^{1}H NMR (500 MHz, $CDCl_3$) δ 8.52 (d, *J* = 4.9 Hz, 1H), 7.68 – 7.61 (m, 1H), 7.38 (d, *J* = 7.9 Hz, 1H), 7.15 (dd, *J* = 7.5, 4.9 Hz, 1H), 3.82 (s, 2H), 2.38 (d, *J* = 6.9 Hz, 2H), 1.83 – 1.70 (m, 1H), 0.94 (d, *J* = 6.7 Hz, 6H) ppm. ^{13}C NMR (126 MHz, $CDCl_3$) δ 159.24, 149.33, 136.76, 123.16, 121.93, 41.00, 38.86, 28.42, 22.14 ppm. HRMS (ESI) m/z calcd. for $C_{10}H_{16}N^{32}S^+$ $[M+H]^+$ 182.09252; found 182.0997.

2-((Benzylthio)methyl)pyridine (4d)[6]

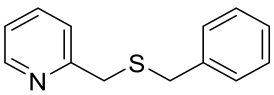

Yellow oil (297.1 mg, 46% yield). ^{1}H NMR (400 MHz, Chloroform-*d*) δ 8.43 (ddd, *J* = 4.9, 1.9, 0.9 Hz, 1H), 7.48 (td, *J* = 7.7, 1.9 Hz, 1H), 7.23 – 7.14 (m, 4H), 7.13 – 7.07 (m, 1H), 7.01 (ddd, *J* = 7.5, 4.9, 1.2 Hz, 1H), 3.63 (s, 2H), 3.57 (s, 2H) ppm. ^{13}C NMR (101 MHz, $CDCl_3$) δ 158.50, 149.13, 137.95, 136.50, 128.93, 128.34, 126.87, 122.99, 121.73, 37.38, 35.81 ppm.

2-(Propylthio)pyridine (4e)[7]

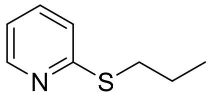

Synthesised from 2-bromopyridine and propanethiol. Pale yellow oil (285 mg, 62% yield). ^{1}H NMR (500 MHz, $CDCl_3$) δ 8.44 – 8.39 (m, 1H), 7.49 – 7.42 (m, 1H), 7.16 (d, *J* = 8.1 Hz, 1H), 6.98 – 6.92 (m, 1H), 3.14 (t, *J* = 7.3 Hz, 2H), 1.79 – 1.70 (m, 2H), 1.04 (t, *J* = 7.4 Hz, 3H) ppm. ^{13}C NMR (126 MHz, $CDCl_3$) δ 159.71, 149.56, 135.92, 122.30, 119.30, 32.21, 22.89, 13.69 ppm.

4-Methyl-2-(propylthio)pyridine (4f)[8]

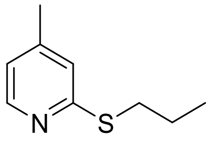

Synthesised from 2-bromo-4-methlypyridine and propanethiol. Pale yellow oil (151 mg, 30% yield). ^{1}H NMR (500 MHz, $CDCl_3$) δ 8.27 (d, *J* = 5.0 Hz, 1H), 7.00 (s, 1H), 6.78 (d, *J* = 5.0 Hz, 1H), 3.13 (t, *J* = 7.3 Hz, 2H), 2.27 (s, 3H), 1.79 – 1.67 (m, 2H), 1.04 (t, *J* = 7.4 Hz, 3H) ppm. ^{13}C NMR (126 MHz, $CDCl_3$) δ 159.41, 149.20, 147.09, 122.81, 120.78, 32.21, 22.94, 20.99, 13.69 ppm.

Benzyl(ethyl)sulfane (4h)[9]

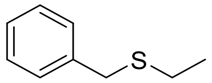

Synthesised from benzylmercaptan and bromoethane. Yellow oil (476 mg, 95% yield). ^{1}H NMR (400 MHz, $CDCl_3$) δ 7.35 – 7.26 (m, 4H), 7.27 – 7.20 (m, 1H), 3.73 (s, 2H), 2.44 (q, *J* = 7.4 Hz, 2H), 1.23 (t, *J* = 7.4 Hz, 3H) ppm. ^{13}C NMR (126 MHz, $CDCl_3$) δ 138.75, 128.95, 128.59, 127.00, 36.02, 25.35, 14.50 ppm.

Synthesis of 1-methyl-2-(propylthio)-1H-imidazole (4g)

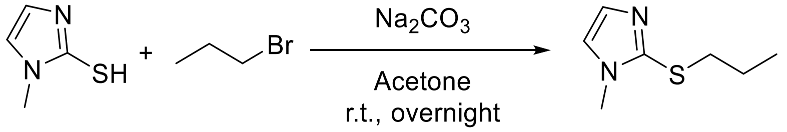

Methimazole (3.0 mmol) and $NaCO_3$ (6 mmol) were stirred in acetone for 20 min at room temperature. Then, 1-bromopropane (3.6 mmol) was added to the mixture and the reaction was stirred overnight. The reaction mixture was extracted with EtOAc and the combined organic layer was washed with H_2O and brine. The collected organic layer was dried over $MgSO_4$ and evaporated under vacuum. The crude product was then purified by flash column chromatography using hexane:EtOAc 6:4 eluent system to afford 1-methyl-2-(propylthio)-1H-imidazole as transparent oil (76.7 mg, 37% yield). ^{1}H NMR (500 MHz, $CDCl_3$) δ 7.05 (d, *J* = 1.4 Hz, 1H), 6.91 (d, *J* = 1.4 Hz, 1H), 3.61 (s, 3H), 3.03 (t, *J* = 7.3 Hz, 2H), 1.75 – 1.63 (m, 2H), 1.01 (t, *J* = 7.3 Hz, 3H) ppm. ^{13}C NMR (126 MHz, $CDCl_3$) δ 142.20, 129.37, 122.10, 36.43, 33.33, 23.27, 13.38 ppm. HRMS (ESI) m/z calcd. for $C_7H_{13}N_2{}^{32}S^+$ $[M+H]^+$ 157.07212; found 157.0791.

Synthesis of (4-bromophenyl)(ethyl)sulfane (4i)[10]

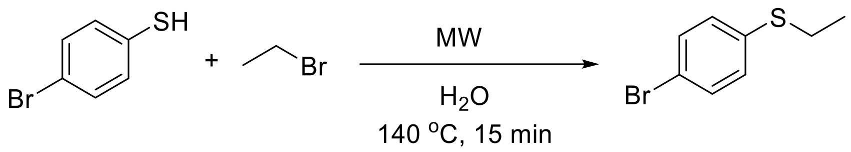

Bromoethane (3.6 mmol) and 4-bromothiophenol (3.0 mmol) were added to a microwave (MW) vial and dissolved in water (10 mL). K_2CO_3 (4.5 mmol) and NaI (0.3 mmol) were then added to the vial and the resulting reaction mixture was stirred at 140 °C in intervals of 5 minutes until completion was observed on TLC under microwave irradiation. The reaction was then extracted with EtOAc and the collected organic layers were washed with water and brine. The collected organic layer was dried over $MgSO_4$ and evaporated under vacuum. The crude product was then purified by flash column chromatography using hexane : EtOAc 85:5 eluent system to afford pale yellow oil (470 mg, 97% yield). ^{1}H NMR (400 MHz, $CDCl_3$) δ 7.68 – 7.60 (m, 2H), 7.50 – 7.42 (m, 2H), 2.88 (dq, *J* = 13.3, 7.4 Hz, 1H), 2.72 (dq, *J* = 13.3, 7.4 Hz, 1H), 1.18 (t, *J* = 7.4 Hz, 3H) ppm. ^{13}C NMR (101 MHz, $CDCl_3$) δ 142.62, 132.46, 125.90, 125.45, 50.36, 5.89 ppm.

The sulfides **4j, 4k, 4l, 4m, 4n, 4o** were commercially available.

11. General procedure for the synthesis of racemic sulfoxides 5a-k, 5n and 5o

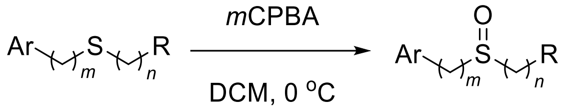

The appropriate sulfide (2.0 mmol) was dissolved in DCM and stirred at 0 °C. *m*CPBA (2.0 mmol) in DCM was added dropwise and the reaction was monitored by TLC for 1 – 24 h until completion. The reaction was dried under reduced pressure and purified by flash column chromatography using an appropriate eluent mixture of EtOAc and hexane to afford the resulting sulfoxide.

2-((Propylsulfinyl)methyl)pyridine (5a)

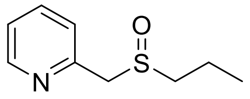

Yellow oil (128.3 mg, 35% yield). ^{1}H NMR (400 MHz, $CDCl_3$) δ 8.60 (d, *J* = 4.6 Hz, 1H), 7.75 – 7.65 (m, 1H), 7.37 (d, *J* = 7.8 Hz, 1H), 7.31 – 7.23 (m, 1H), 4.18 (d, *J* = 12.8 Hz, 1H), 4.08 (d, *J* = 12.8 Hz, 1H), 2.75 – 2.58 (m, 2H), 1.89 – 1.70 (m, 2H), 1.05 (t, *J* = 7.4 Hz, 3H) ppm. ^{13}C NMR (101 MHz, $CDCl_3$) δ 150.99, 149.88, 137.09, 125.53, 123.21, 59.66, 53.71, 16.38, 13.49 ppm. HRMS (ESI) m/z calcd. for $C_9H_{14}ON^{32}S^+$ $[M+H]^+$ 184.07906; found 184.0793.

2-((Ethylsulfinyl)methyl)pyridine (5b)[11]

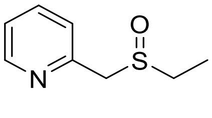

Colourless oil (242 mg, 81% yield). v_{max}/cm^{-1}: 2970, 2930, 1433, 1041. ^{1}H NMR (400 MHz, $CDCl_3$) δ 8.59 (ddd, *J* = 4.9, 1.9, 0.9 Hz, 1H), 7.75 – 7.66 (m, 1H), 7.40 – 7.33 (m, 1H), 7.30 – 7.22 (m, 1H), 4.17 (d, *J* = 12.8 Hz, 1H), 4.07 (d, *J* = 12.8 Hz, 1H), 2.78 (dq, *J* = 13.2, 7.5 Hz, 1H), 2.66 (dq, *J* = 13.2, 7.5 Hz, 1H), 1.34 (t, *J* = 7.5 Hz, 3H) ppm. ^{13}C NMR (101 MHz, $CDCl_3$) δ 150.98, 149.91, 137.07, 125.48, 123.18, 58.94, 45.04, 6.84 ppm.

2-((Isobutylsulfinyl)methyl)pyridine (5c)

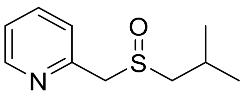

Pale yellow oil (236.8 mg ,60% yield). ^{1}H NMR (500 MHz, $CDCl_3$) δ 8.61 (d, *J* = 4.5 Hz, 1H), 7.74 – 7.67 (m, 1H), 7.37 (d, *J* = 7.8 Hz, 1H), 7.30 – 7.23 (m, 1H), 4.17 (d, *J* = 12.8 Hz, 1H), 4.07 (d, *J* = 12.8 Hz, 1H), 2.65 (dd, *J* = 12.9, 5.0 Hz, 1H), 2.52 (dd, *J* = 12.9, 9.3 Hz, 1H), 2.27 – 2.15 (m, 1H), 1.08 – 1.03 (m, 6H) ppm. ^{13}C NMR (126 MHz, $CDCl_3$) δ 151.06, 150.06, 136.96, 125.51, 123.18, 61.24, 60.39, 24.03, 23.03, 21.77 ppm. HRMS (ESI) m/z calcd. for $C_{10}H_{16}ON^{32}S^+$ $[M+H]^+$ 198.08743; found 198.0947.

2-((Benzylsulfinyl)methyl)pyridine (5d)

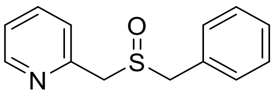

Red-brown solid (402.1 mg, yield 87%); mp: 71 °C. ^{1}H NMR (400 MHz, $CDCl_3$) δ 8.59 (ddd, *J* = 5.0, 1.9, 1.0 Hz, 1H), 7.65 (td, *J* = 7.7, 1.8 Hz, 1H), 7.40 – 7.26 (m, 6H), 7.22 (ddd, *J* = 7.6, 4.9, 1.2 Hz, 1H), 4.10 (dd, *J* = 12.9, 9.3 Hz, 2H), 3.92 (dd, *J* = 12.9, 8.5 Hz, 2H) ppm. ^{13}C NMR (101 MHz, $CDCl_3$) δ 150.94, 149.83, 136.74, 130.43, 129.90, 128.84, 128.28, 125.60, 122.98, 57.82, 57.25 ppm. HRMS (ESI) m/z calcd. for $C_{13}H_{14}ON^{32}S^+$ $[M+H]^+$ 232.07178; found 232.0799.

2-(Propylsulfinyl)pyridine (5e)[12]

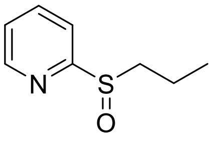

Pale yellow oil (203.1 mg, 61% yield). ^{1}H NMR (500 MHz, $CDCl_3$) δ 8.61 (d, *J* = 4.7 Hz, 1H), 8.02 – 7.96 (m, 1H), 7.96 – 7.87 (m, 1H), 7.39 – 7.31 (m, 1H), 3.12 – 3.00 (m, 1H), 2.91 – 2.82 (m, 1H), 1.99 – 1.85 (m, 1H), 1.71 – 1.55 (m, 1H), 1.04 (t, *J* = 7.4 Hz, 3H) ppm. ^{13}C NMR (126 MHz, $CDCl_3$) δ 165.00, 149.66, 138.00, 124.53, 120.08, 56.45, 15.63, 13.34 ppm.

4-Methyl-2-(propylsulfinyl)pyridine (5f)

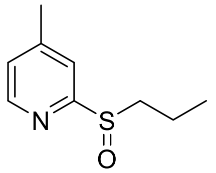

Pale yellow oil (208.6 mg, 57% yield). ^{1}H NMR (500 MHz, $CDCl_3$) δ 8.46 (d, *J* = 4.9 Hz, 1H), 7.82 (s, 1H), 7.16 (d, *J* = 4.9 Hz, 1H), 3.11 – 3.01 (m, 1H), 2.90 – 2.80 (m, 1H), 2.46 (s, 3H), 1.99 – 1.85 (m, 1H), 1.69 – 1.56 (m, 1H), 1.05 (t, *J* = 7.3 Hz, 3H) ppm. ^{13}C NMR (126 MHz, $CDCl_3$) δ 164.60, 149.90, 149.45, 125.49, 120.66, 56.43, 21.46, 15.68, 13.36 ppm. HRMS (ESI) m/z calcd. for $C_9H_{14}ON^{32}S^+$ $[M+H]^+$ 184.07178; found 184.0792.

1-Methyl-2-(propylsulfinyl)-1H-imidazole (5g)

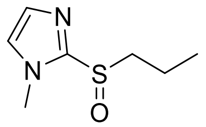

Pale yellow oil (213.4 mg, 62% yield). ^{1}H NMR (500 MHz, $CDCl_3$) δ 7.14 (d, *J* = 1.4 Hz, 1H), 7.01 (d, *J* = 1.4 Hz, 1H), 3.95 (s, 3H), 3.47 – 3.37 (m, 1H), 3.31 – 3.22 (m, 1H), 1.87 – 1.76 (m, 2H), 1.10 (t, *J* = 7.4 Hz, 3H) ppm. ^{13}C NMR (126 MHz, $CDCl_3$) δ 145.10, 129.69, 125.04, 54.49, 33.82, 16.32, 13.36 ppm. HRMS (ESI) m/z calcd. for $C_7H_{13}ON_2{}^{32}S^+$ $[M+H]^+$ 173.06703; found 173.0744.

((Ethylsulfinyl)methyl)benzene (5h)[13,14]

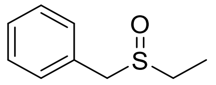

White solid (198 mg, 66% yield). ^{1}H NMR (500 MHz, $CDCl_3$) δ 7.40 – 7.32 (m, 3H), 7.31 – 7.27 (m, 2H), 4.02 (d, *J* = 13.0 Hz, 1H), 3.93 (d, *J* = 13.0 Hz, 1H), 2.72 – 2.49 (m, 2H), 1.33 (t, *J* = 7.5 Hz, 3H) ppm. ^{13}C NMR (126 MHz, $CDCl_3$) δ 130.18, 130.11, 129.14, 128.47, 57.85, 44.26, 6.71 ppm.

1-Bromo-4-(ethylsulfinyl)benzene (5i)[15]

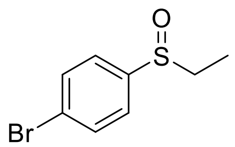

Colourless oil (275 mg, 92% yield). ^{1}H NMR (400 MHz, $CDCl_3$) δ 7.39 (d, *J* = 8.6 Hz, 2H), 7.17 (d, *J* = 8.6 Hz, 2H), 2.91 (q, *J* = 7.3 Hz, 2H), 1.30 (t, *J* = 7.3 Hz, 3H) ppm. ^{13}C NMR (101 MHz, $CDCl_3$) δ 135.99, 131.86, 130.43, 119.48, 27.70, 14.29 ppm.

1-Chloro-3-(methylsulfinyl)benzene (5j)[14]

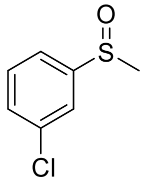

Pale yellow oil (362 mg, 99% yield). ^{1}H NMR (400 MHz, Chloroform-*d*) δ 7.68 – 7.66 (m, 1H), 7.52 – 7.45 (m, 3H), 2.74 (s, 3H) ppm. ^{13}C NMR (101 MHz, $CDCl_3$) δ 148.06, 135.89, 131.34, 130.73, 123.81, 121.75, 44.18 ppm.

1-Fluoro-4-(methylsulfinyl)benzene (5k)[16]

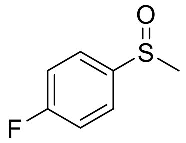

Colourless oil (102 mg, 92% yield). ^{1}H NMR (400 MHz, $CDCl_3$) δ 7.70 – 7.60 (m, 2H), 7.27 – 7.17 (m, 2H), 2.71 (s, 3H) ppm. ^{13}C NMR (101 MHz, $CDCl_3$) δ 165.73, 163.23, 141.31, 126.03, 125.94, 116.95, 116.72, 44.31, 44.30 ppm. ^{19}F NMR (376 MHz, $CDCl_3$) δ -108.62 ppm.

1-Chloro-4-(methylsulfinyl)benzene (5n)[17]

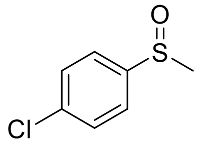

White solid (359 mg, 99% yield). ^{1}H NMR (400 MHz, Chloroform-*d*) δ 7.62 – 7.57 (m, 2H), 7.54 – 7.49 (m, 2H), 2.72 (s, 3H) ppm. ^{13}C NMR (101 MHz, $CDCl_3$) δ 144.1, 137.1, 129.5, 124.8, 43.9 ppm.

1-Chloro-2-(methylsulfinyl)benzene (5o)[18]

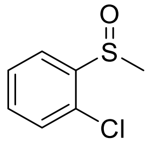

Pale yellow oil (125 mg, 57% yield). ^{1}H NMR (500 MHz, $CDCl_3$) δ 7.97 (dd, J = 7.9, 1.7 Hz, 1H), 7.54 (td, J = 7.6, 1.3 Hz, 1H), 7.45 (td, J = 7.6, 1.7 Hz, 1H), 7.40 (dd, J = 7.9, 1.3 Hz, 1H), 2.83 (s, 3H) ppm. ^{13}C NMR (126 MHz, $CDCl_3$) δ 143.8, 132.1, 130.0, 129.9, 128.3, 125.5, 41.8 ppm.

The sulfoxides **5l, 5m** were all commercially available.

12. General procedure for the synthesis of sulfones 6

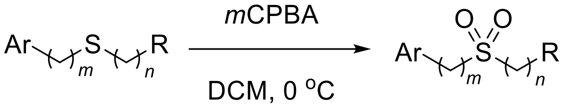

The appropriate sulfide **4** (2.0 mmol) was dissolved in DCM and stirred at 0 °C. *m*CPBA (4.0 mmol) in DCM was added dropwise and the reaction was monitored by TLC for 1 – 24 h until completion. The reaction was dried under reduced pressure and purified by flash column chromatography using an appropriate eluent mixture of EtOAc and hexane to afford the resulting sulfone **6**.

2-((propylsulfonyl)methyl)pyridine (6a)[19]

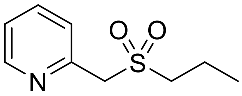

Colourless oil (271 mg, 68% yield). ^{1}H NMR (400 MHz, $CDCl_3$) δ 8.63 – 8.57 (m, 1H), 7.76 (td, *J* = 7.7, 1.8 Hz, 1H), 7.52 (dt, *J* = 7.7, 1.1 Hz, 1H), 7.32 (ddd, *J* = 7.6, 4.9, 1.2 Hz, 1H), 4.40 (s, 2H), 3.00 – 2.92 (m, 2H), 1.96 – 1.82 (m, 2H), 1.04 (t, *J* = 7.4 Hz, 3H) ppm. ^{13}C NMR (101 MHz, $CDCl_3$) δ 149.83, 149.71, 137.50, 126.20, 123.78, 61.47, 53.82, 15.75, 13.24 ppm.

2-((ethylsulfonyl)methyl)pyridine (6b)[19]

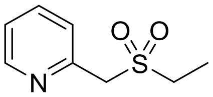

White solid (260 mg, 70% yield). ^{1}H NMR (400 MHz, $CDCl_3$) δ 8.59 (ddd, *J* = 4.9, 1.8, 0.9 Hz, 1H), 7.75 (td, *J* = 7.7, 1.9 Hz, 1H), 7.51 (dt, *J* = 7.8, 1.1 Hz, 1H), 7.31 (ddd, *J* = 7.6, 4.9, 1.2 Hz, 1H), 4.40 (s, 2H), 3.01 (q, *J* = 7.5 Hz, 2H), 1.40 (t, *J* = 7.5 Hz, 3H) ppm. ^{13}C NMR (101 MHz, $CDCl_3$) δ 149.99, 149.75, 137.36, 126.11, 123.73, 60.73, 46.53, 6.65 ppm.

((ethylsulfonyl)methyl)benzene (6h)[19]

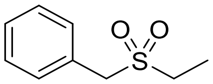

White solid (268 mg, 73% yield). ^{1}H NMR (400 MHz, $CDCl_3$) δ 7.45 – 7.36 (m, 5H), 4.22 (s, 2H), 2.85 (q, *J* = 7.5 Hz, 2H), 1.36 (t, *J* = 7.5 Hz, 3H) ppm. ^{13}C NMR (101 MHz, $CDCl_3$) δ 130.64, 129.25, 129.19, 128.26, 58.93, 45.54, 6.58 ppm.

1-methyl-3-(methylsulfonyl)benzene (6m)[20]

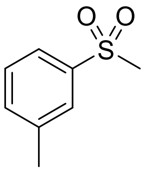

Colourless oil (221 mg, 65% yield). ^{1}H NMR (400 MHz, $CDCl_3$) δ 7.78 – 7.73 (m, 2H), 7.49 – 7.42 (m, 2H), 3.04 (s, 3H), 2.45 (s, 3H) ppm. ^{13}C NMR (101 MHz, $CDCl_3$) δ 140.60, 139.82, 134.59, 129.37, 127.81, 124.58, 44.64, 21.47 ppm.

2-((propylsulfonyl)methyl)pyridine 1-oxide

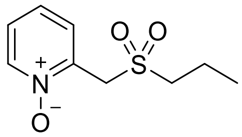

From the **4a** oxidation procedure for synthesising sulfone **6a**, the corresponding over-oxidized product 2-((propylsulfonyl)methyl)pyridine 1-oxide was obtained with 15% yield as white solid. ^{1}H NMR (400 MHz, $CDCl_3$) δ 8.32 – 8.26 (m, 1H), 7.62 – 7.53 (m, 1H), 7.41 – 7.28 (m, 2H), 4.70 (s, 2H), 3.19 – 3.10 (m, 2H), 1.99 – 1.85 (m, 2H), 1.06 (t, J = 7.4 Hz, 3H) ppm. ^{13}C NMR (101 MHz, $CDCl_3$) δ 140.93, 139.62, 129.66, 126.38, 126.31, 57.30, 54.09, 15.84, 13.29 ppm.

13. Blank Experiments

A set of blank experiments with empty pET28a vector *E. coli* CFE were carried out on different substrates. The experiments clearly demonstrates that the biotransformation are catalysed by the BVMO/FMO enzymes.

Table S2. Control experiments with empty pET28a vector *E. coli* CFE

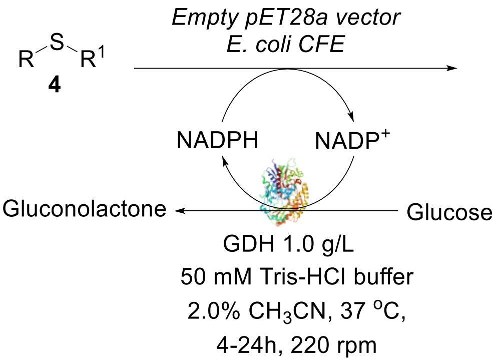

Substrate		Conv. %[a]	ee
**4a**	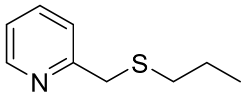	<1	n.d.[b]
**4h**	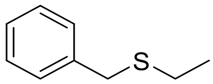	<1	n.d.[b]
**4j**	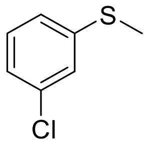	<1	n.d.[b]
**4m**	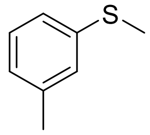	<1	n.d.[b]

[a]Determined by HPLC using an Agilent Eclipse Plus C18 column, monitored at 254 nm. [b]n.d. = not determined.

14. Copies of NMR spectra

2-((Propylthio)methyl)pyridine (4a)

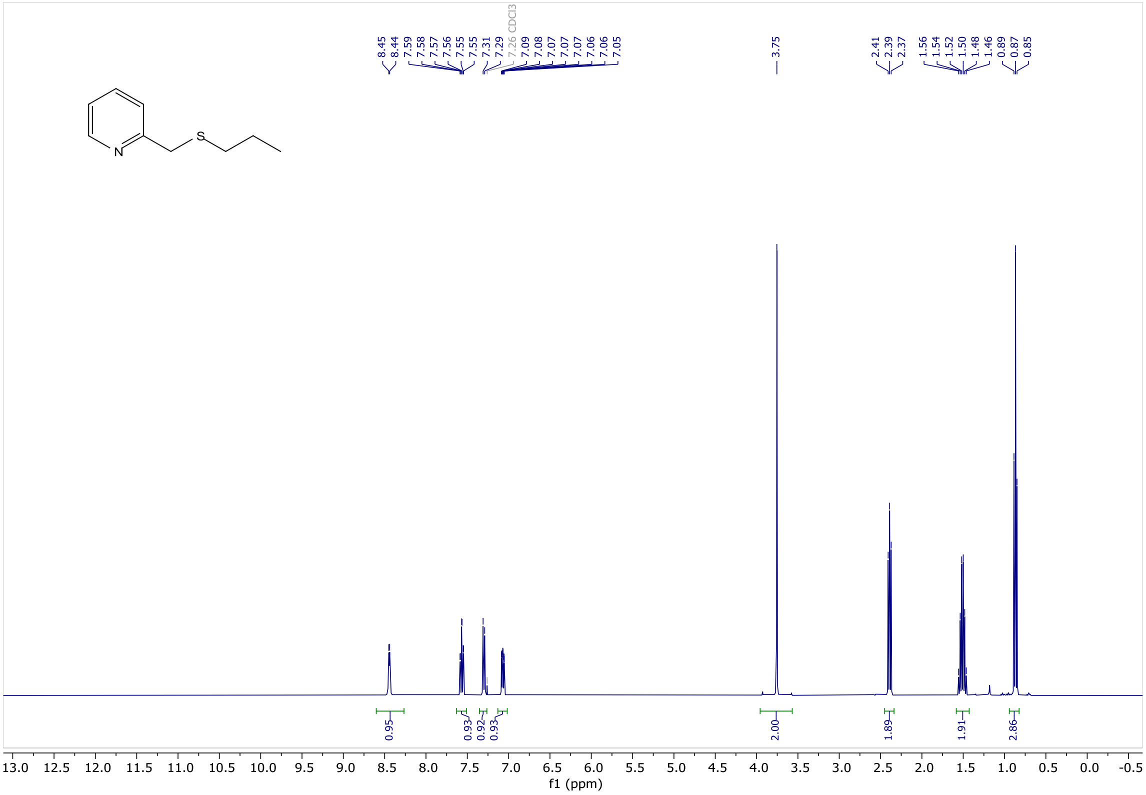

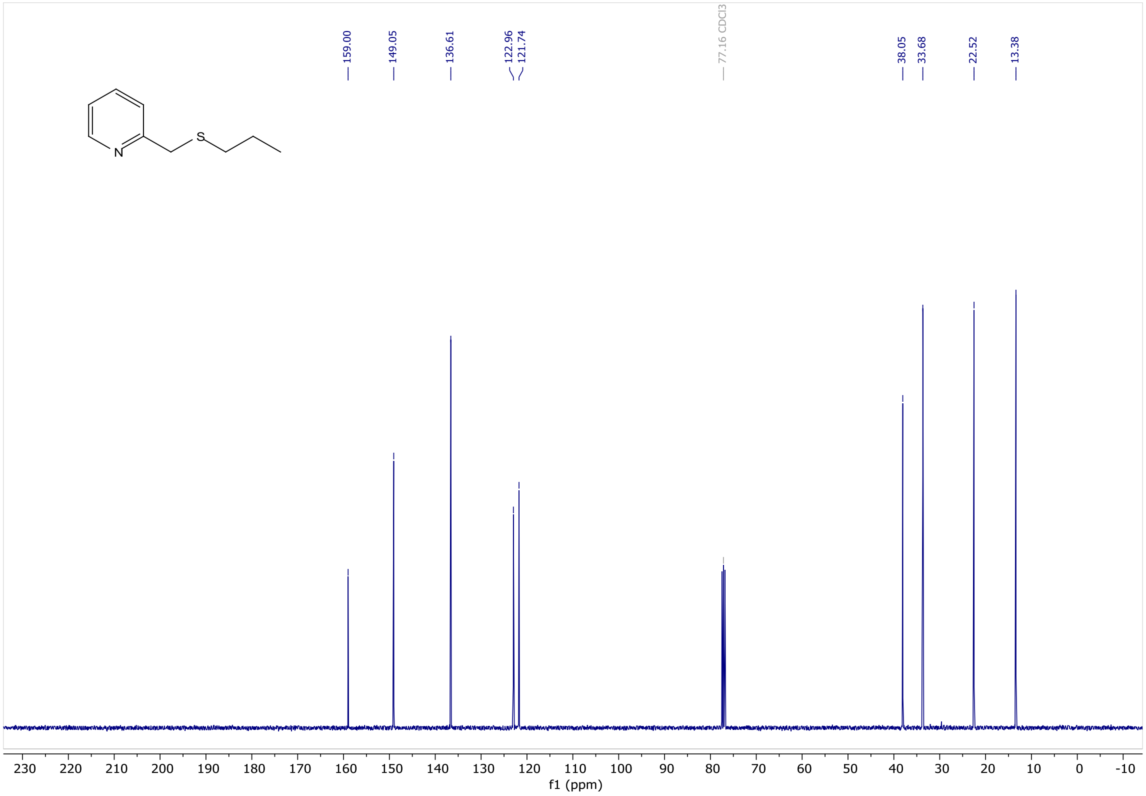

2-((Ethylthio)methyl)pyridine (4b)

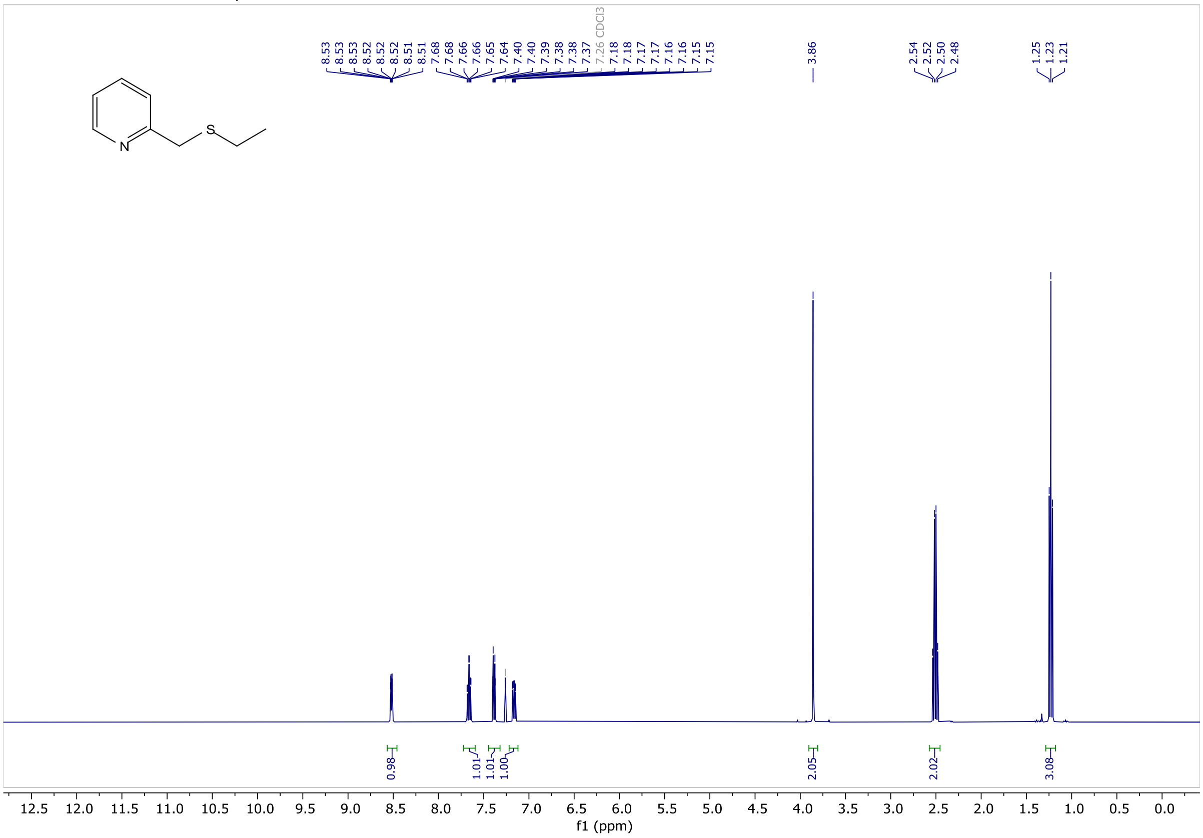

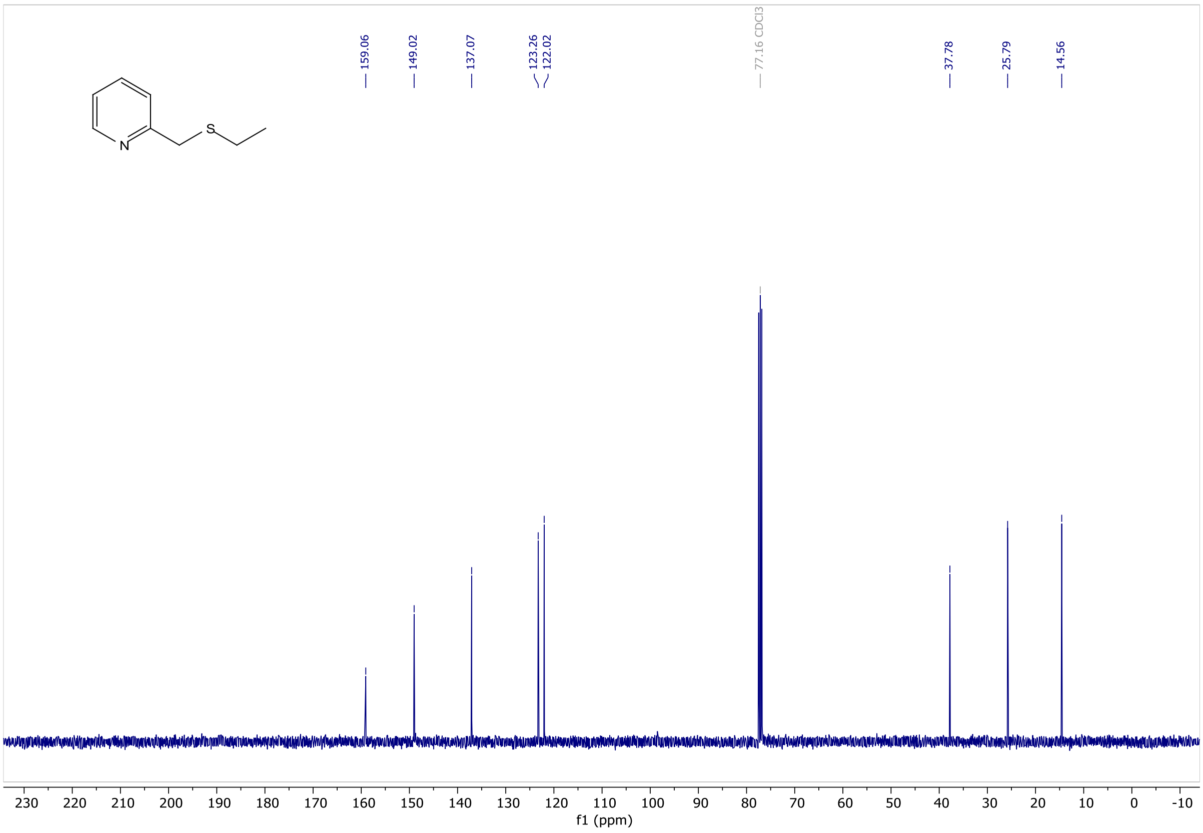

2-((Isobutylthio)methyl)pyridine (4c)

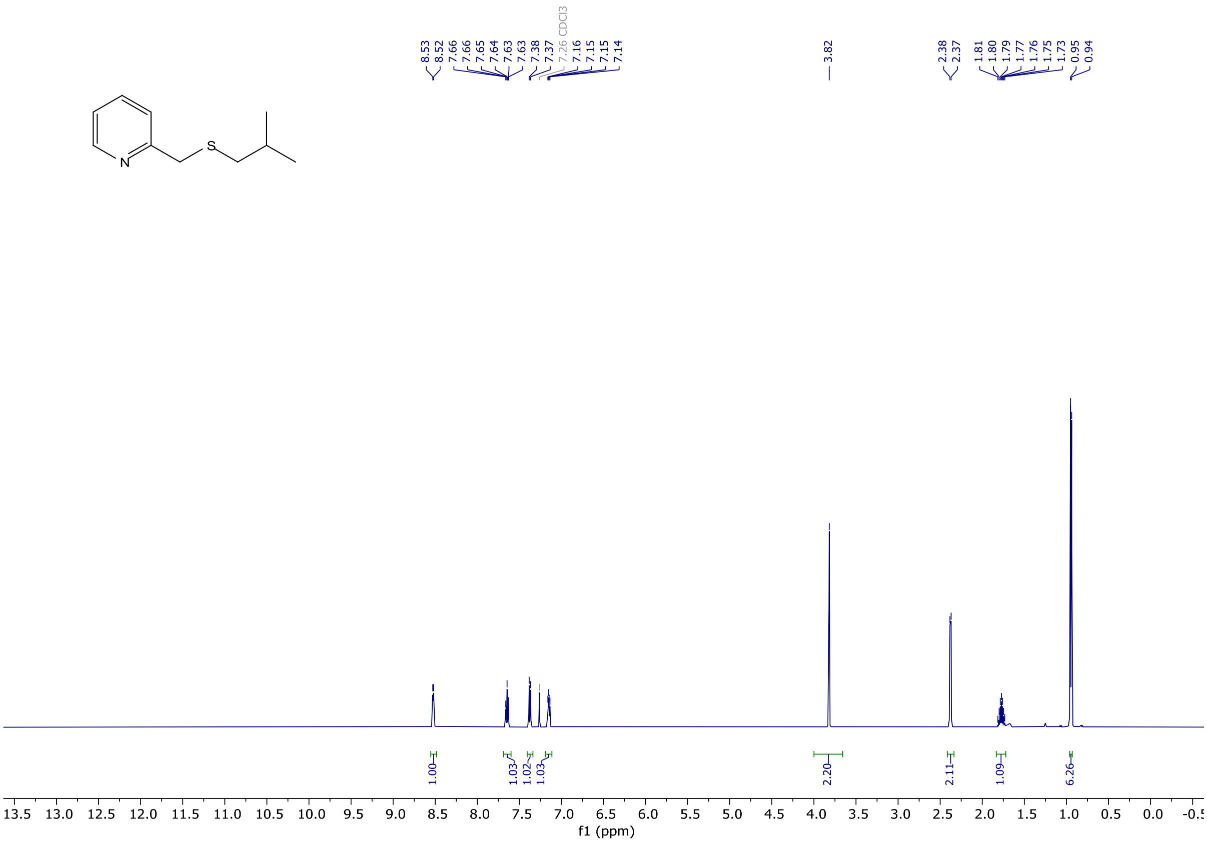

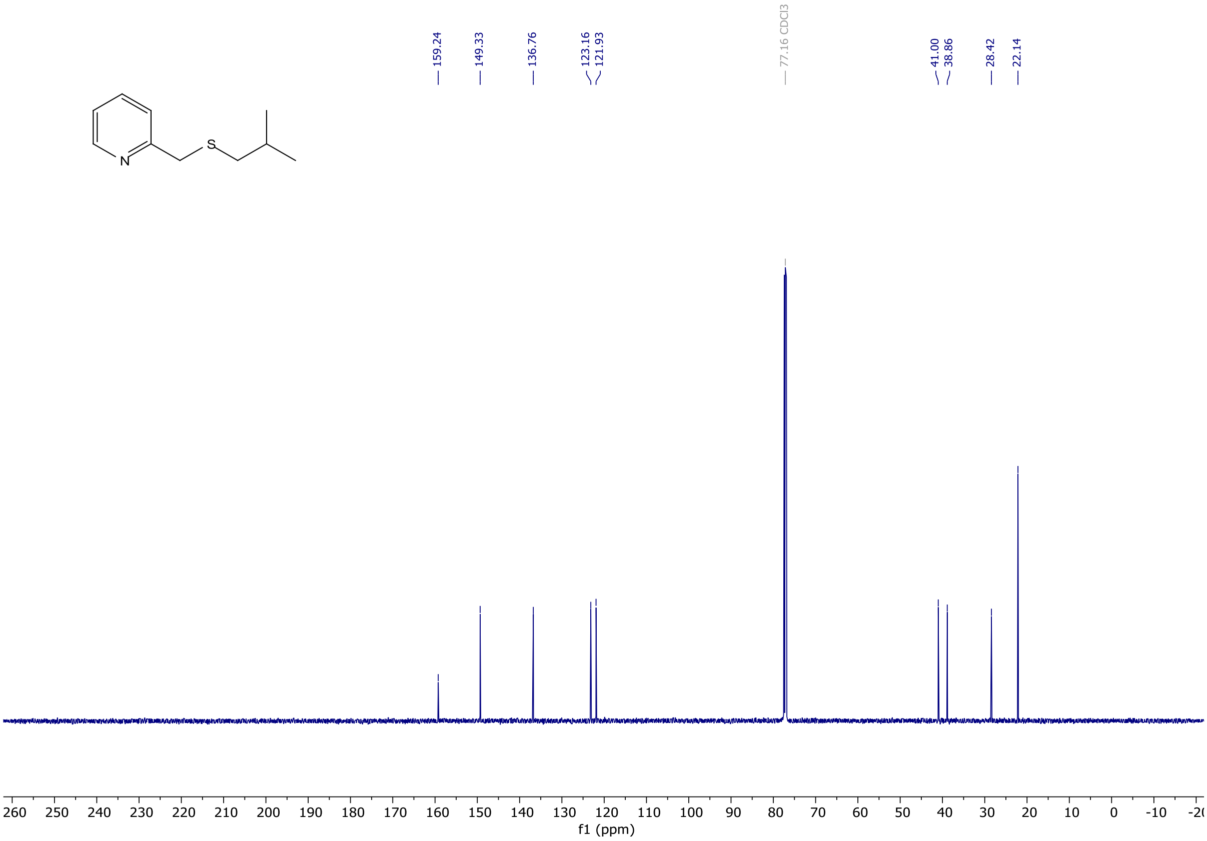

2-((Benzylthio)methyl)pyridine (4d)

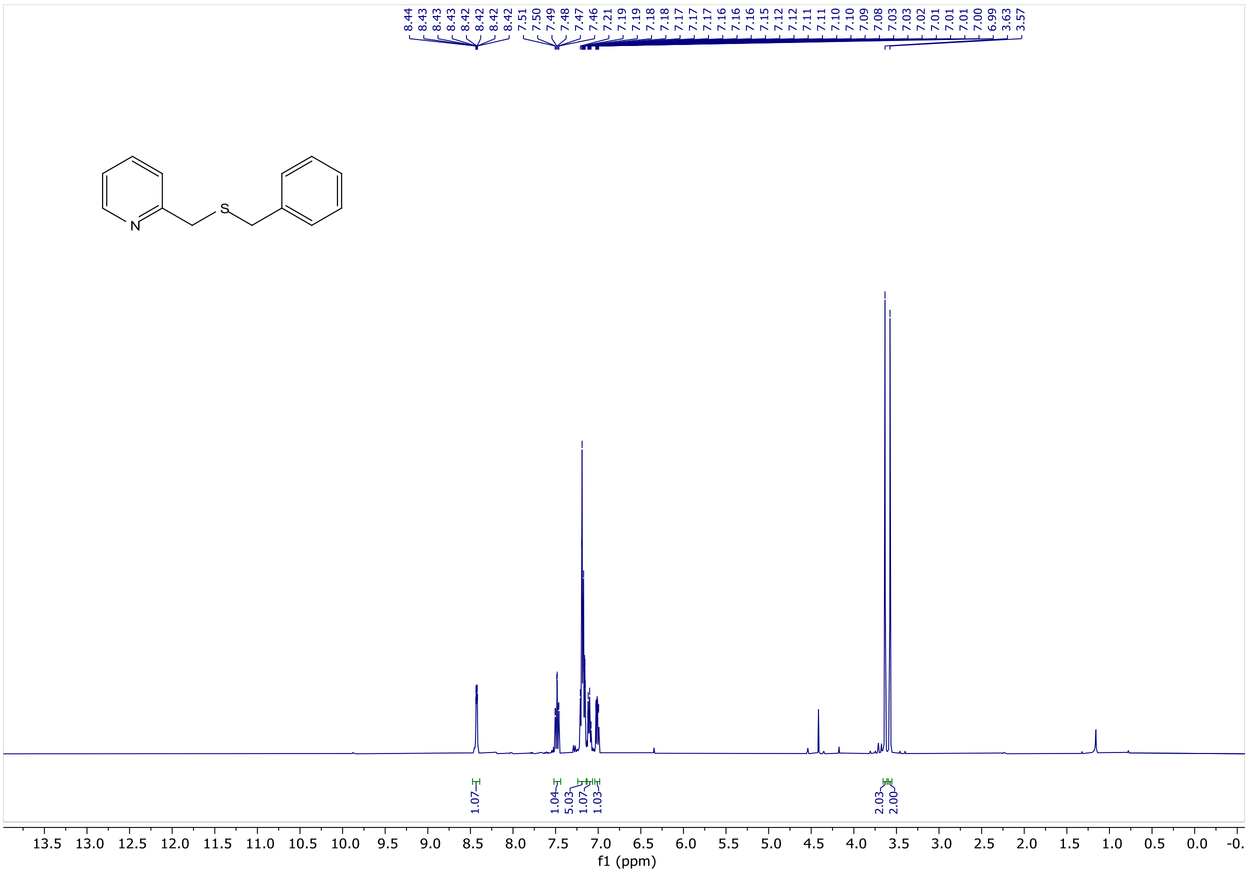

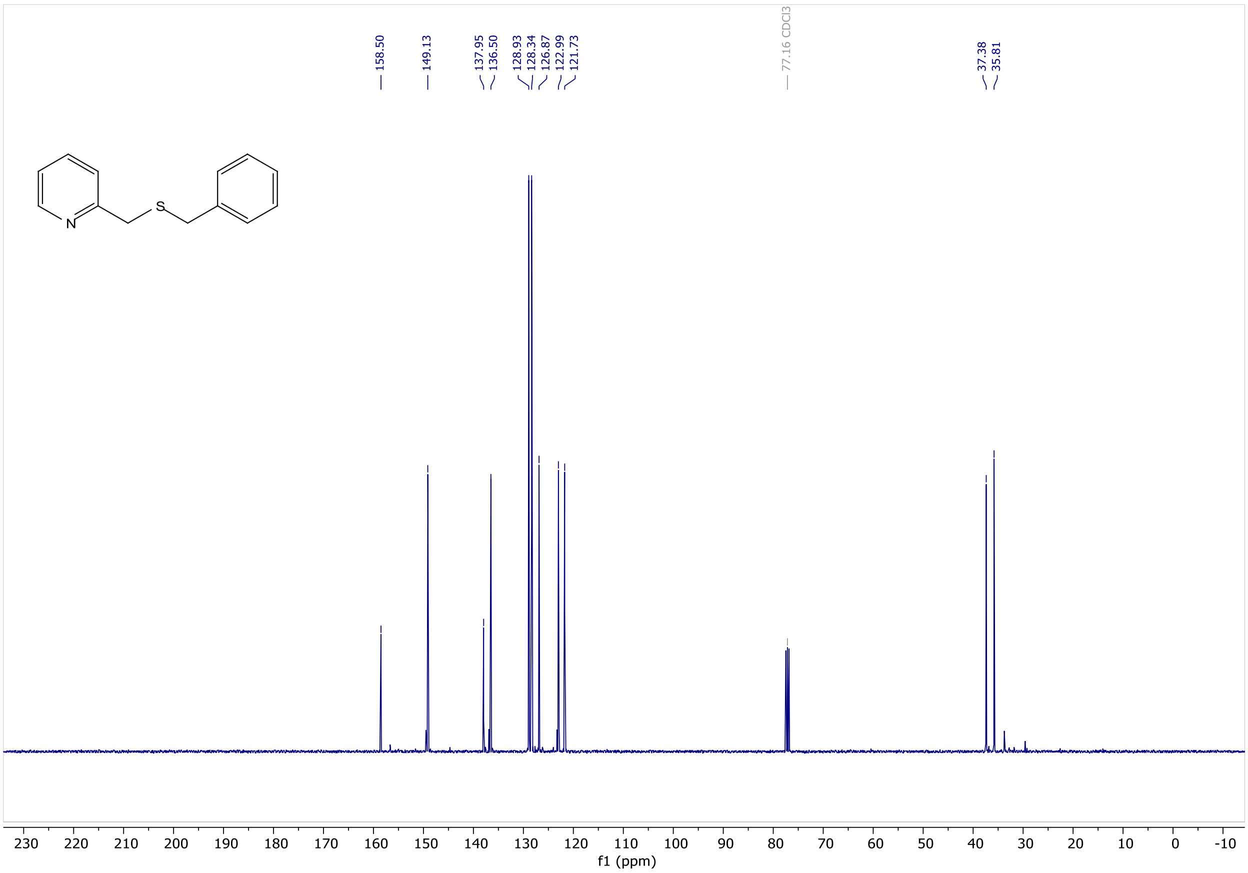

2-(Propylthio)pyridine (4e)

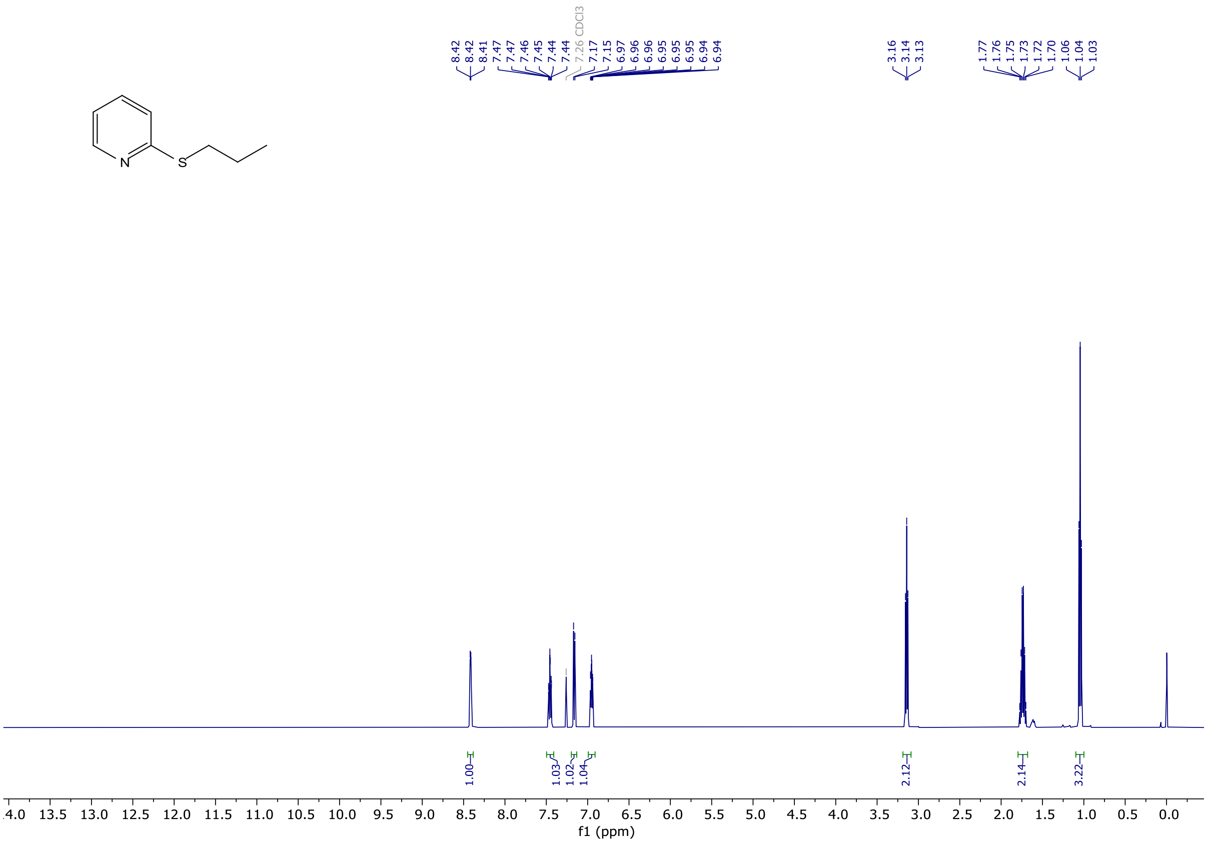

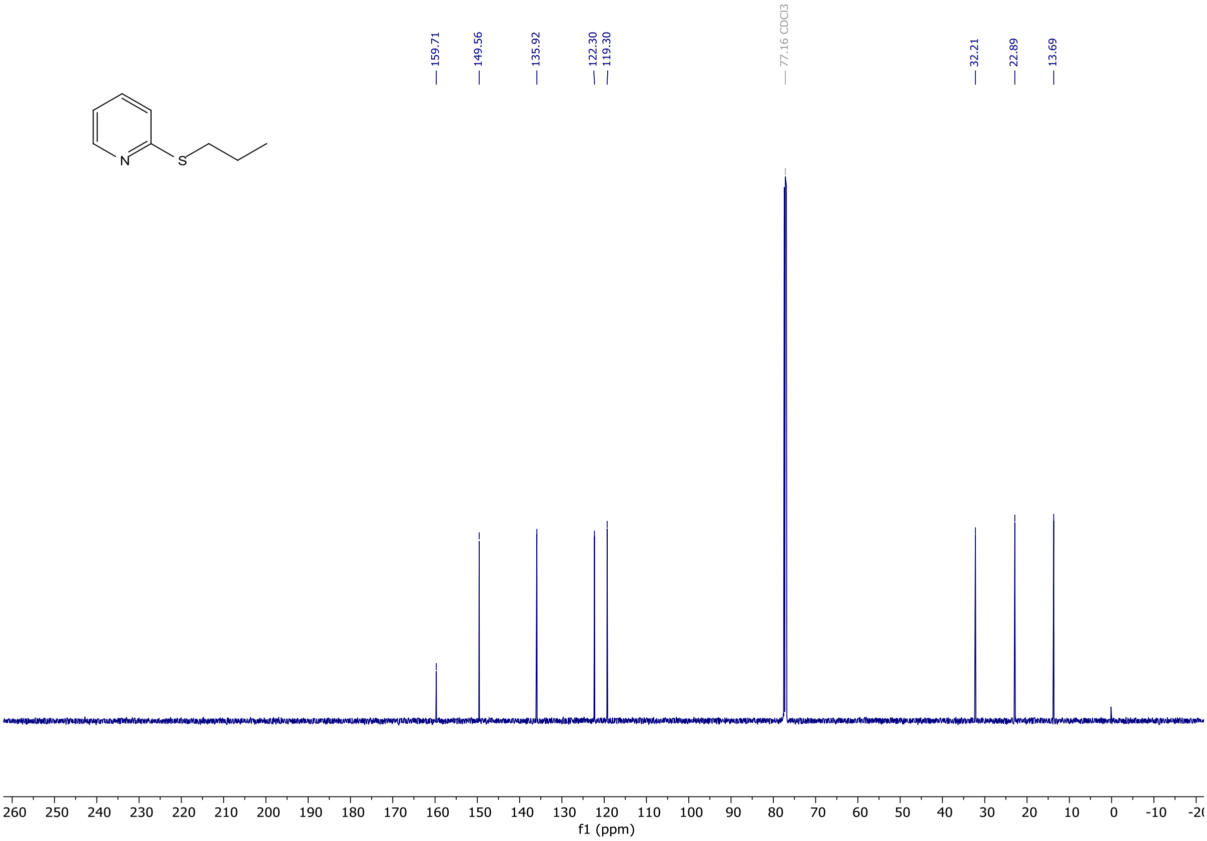

4-Methyl-2-(propylthio)pyridine (4f)

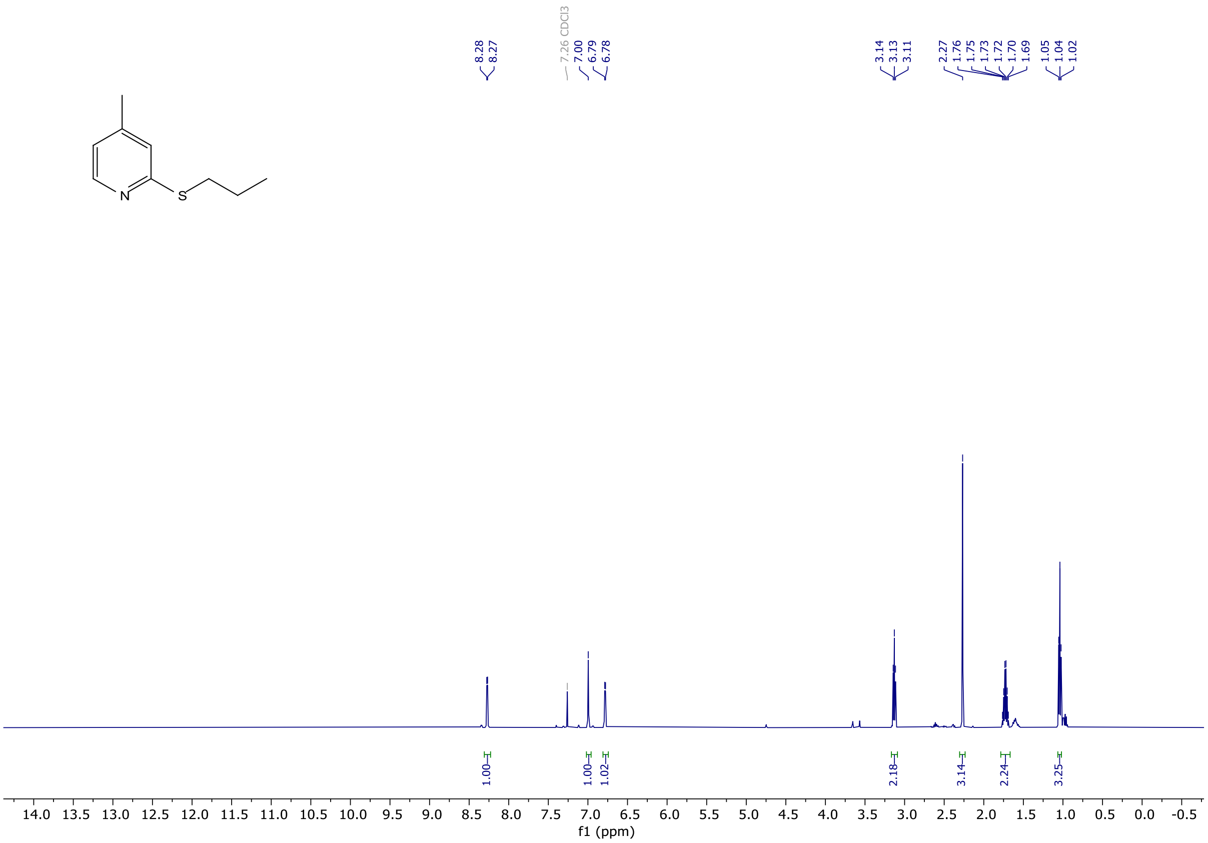

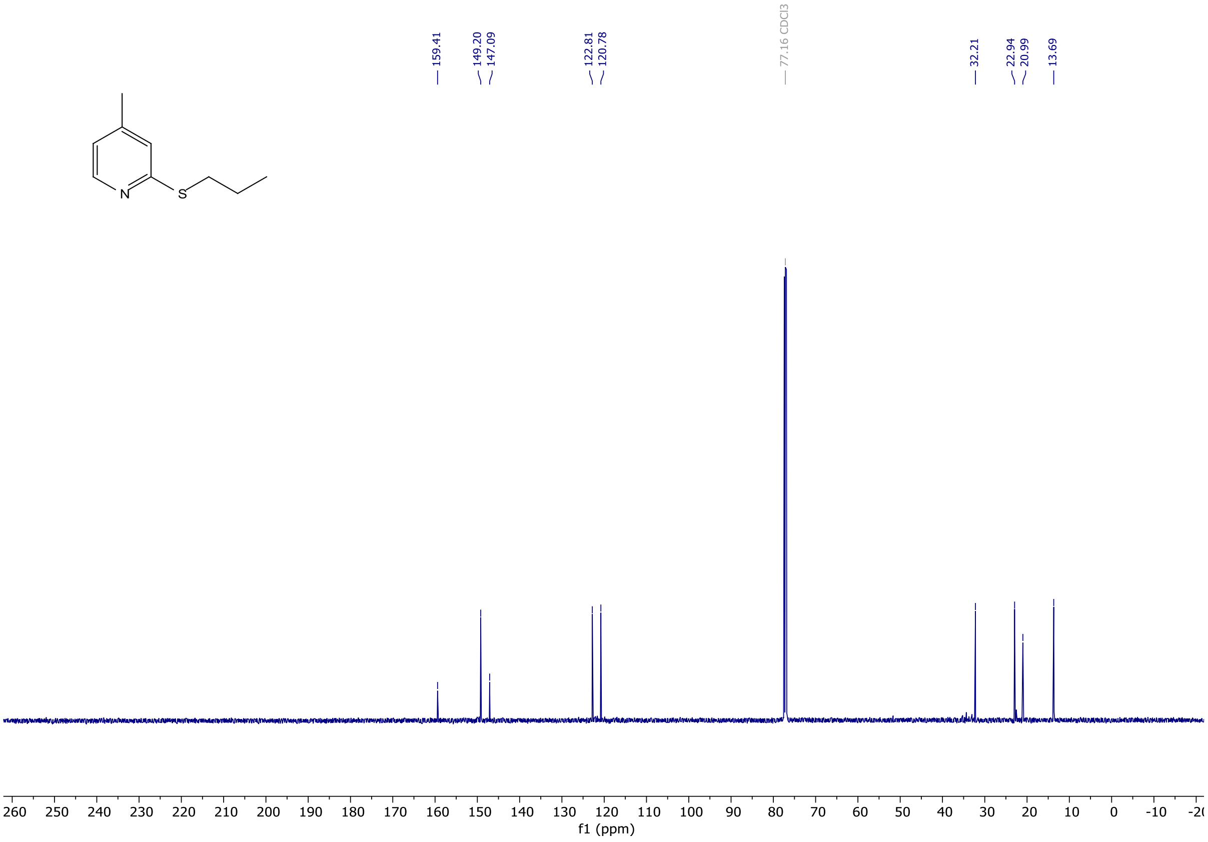

1-Methyl-2-(propylthio)-1H-imidazole (4g)

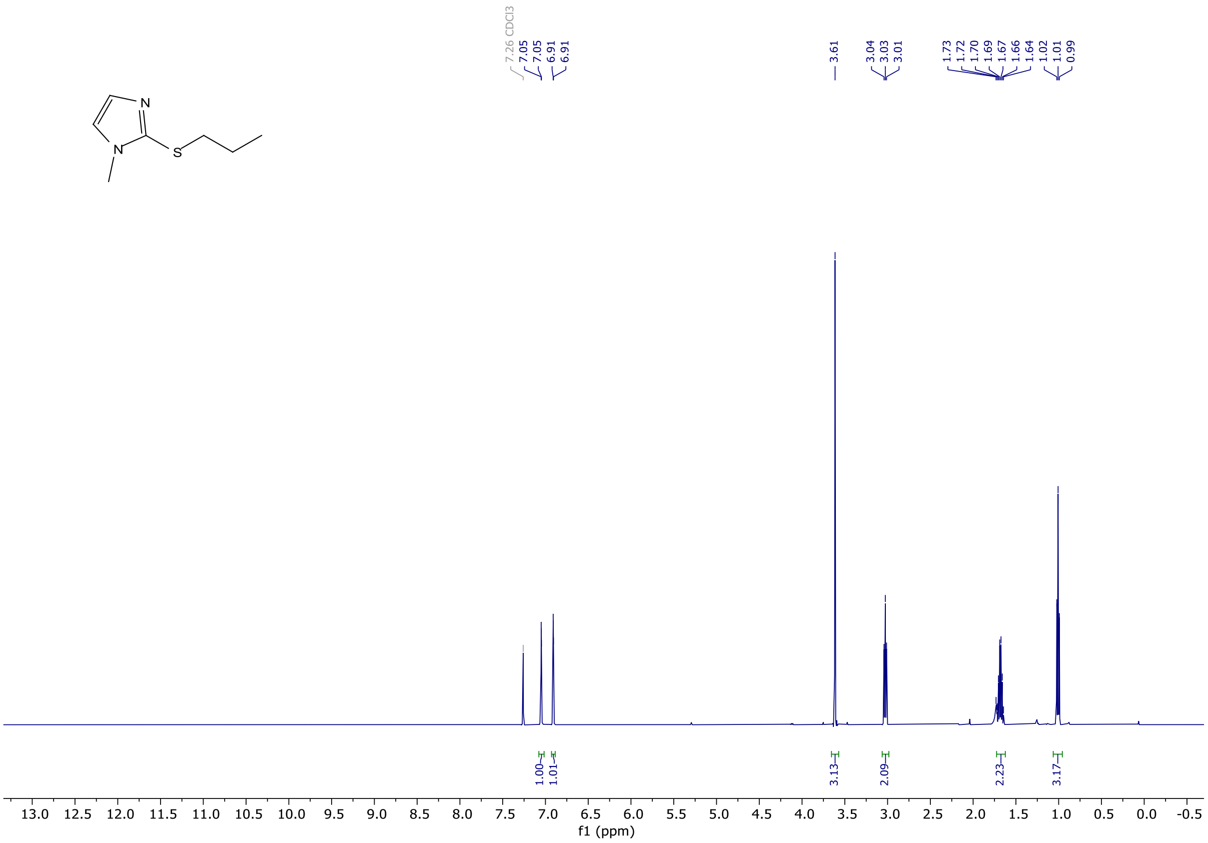

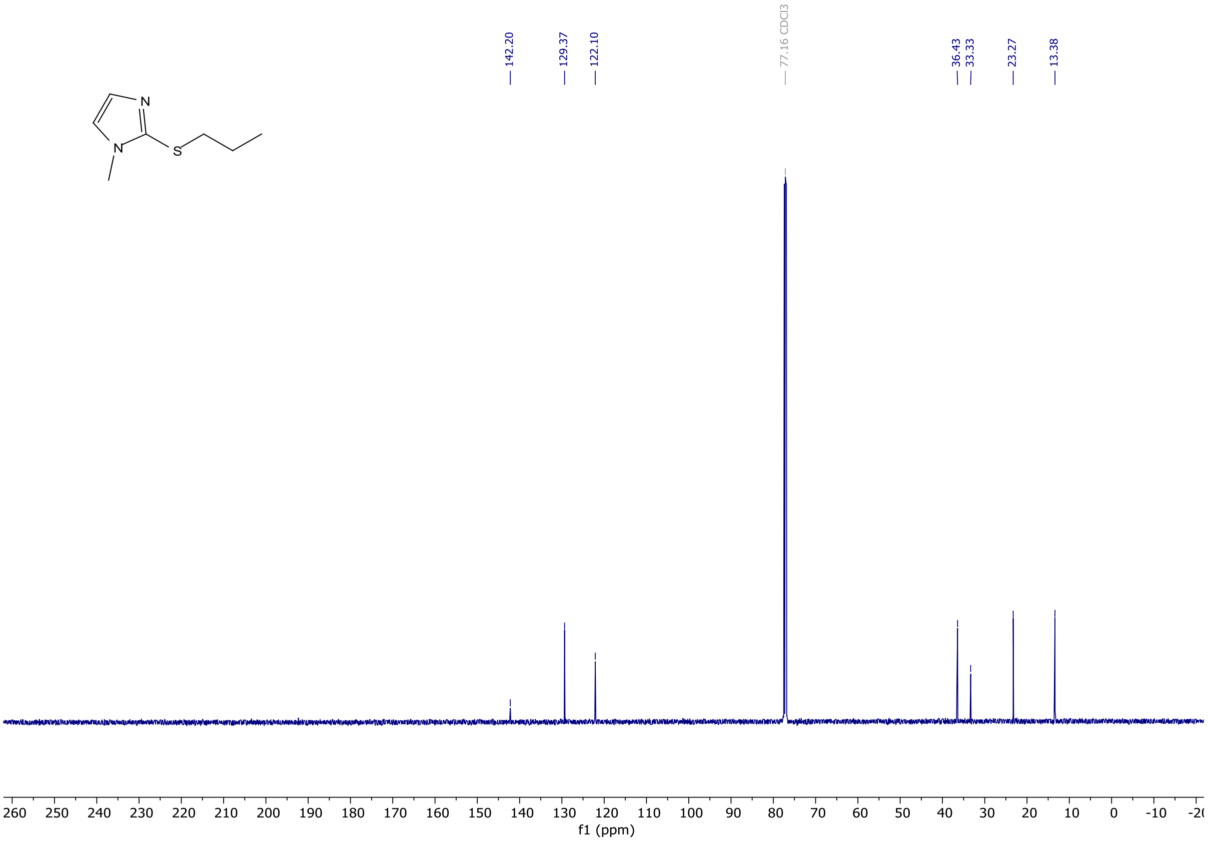

Benzyl(ethyl)sulfane (4h)

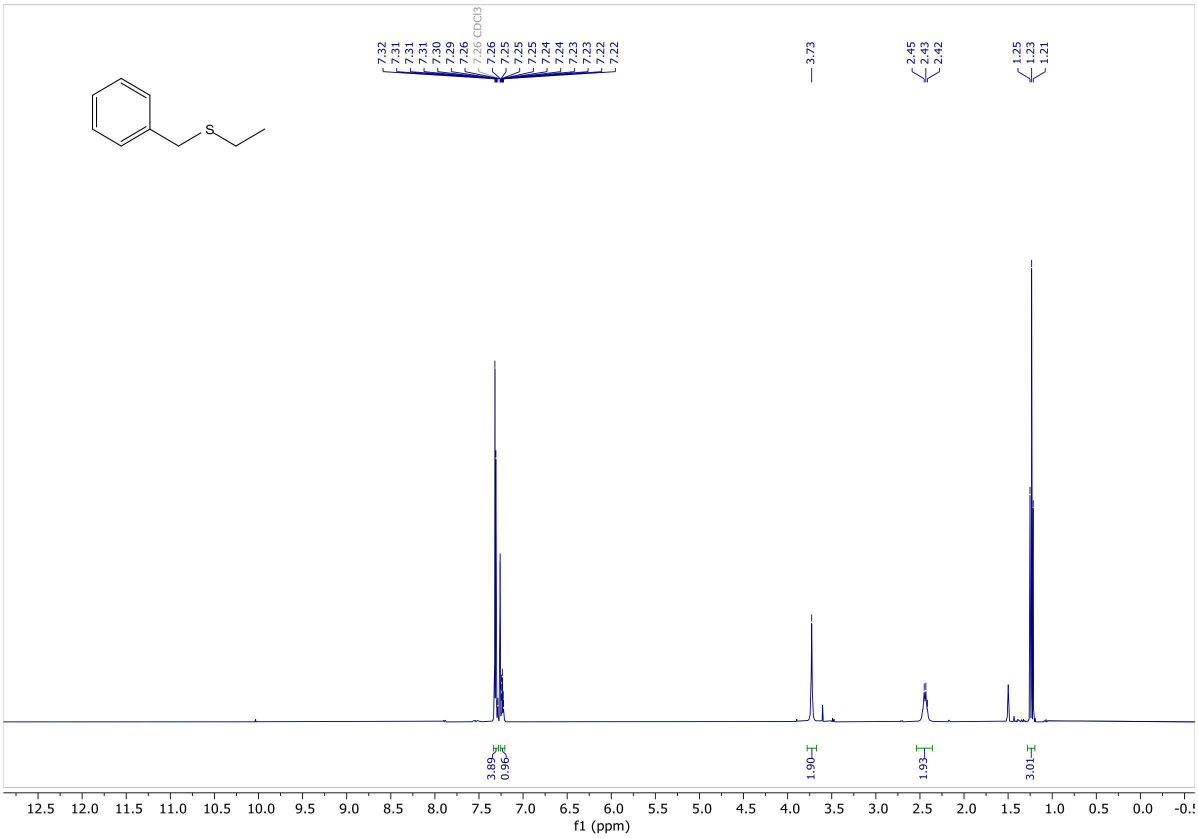

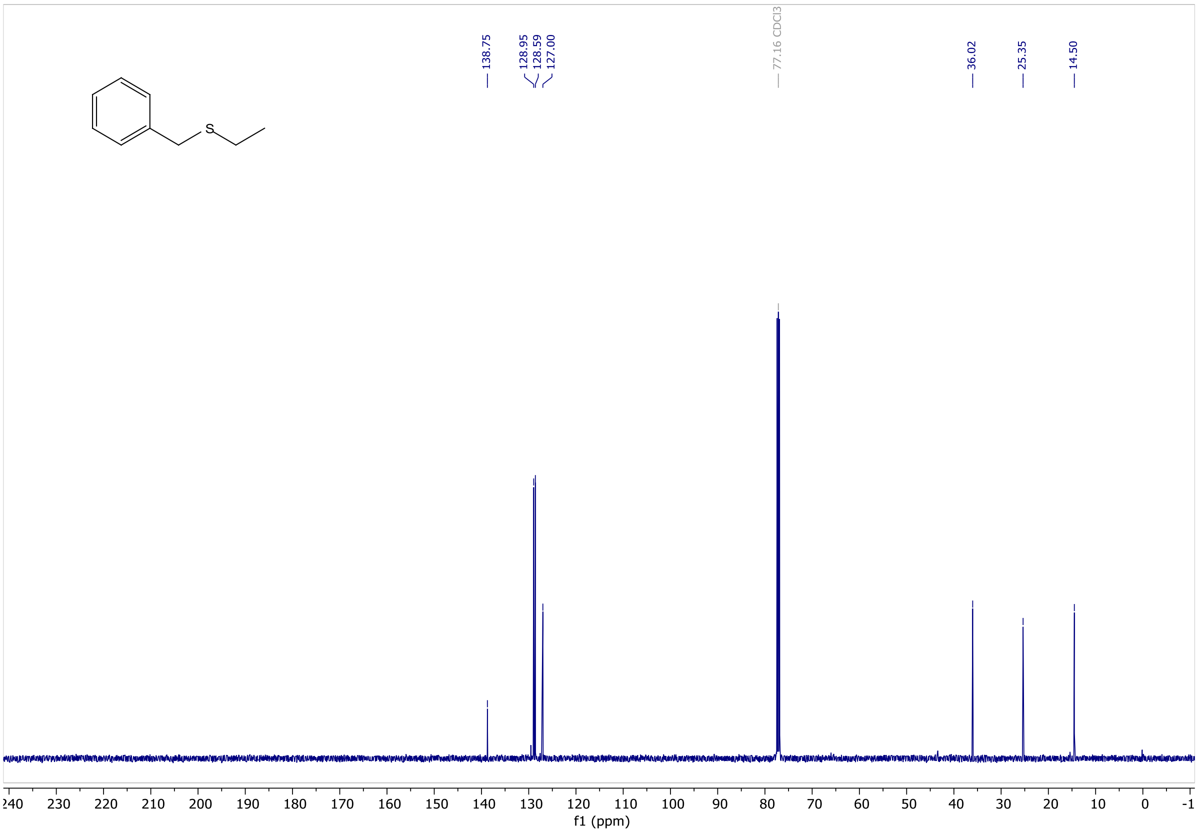

(4-Bromophenyl)(ethyl)sulfane (4i)

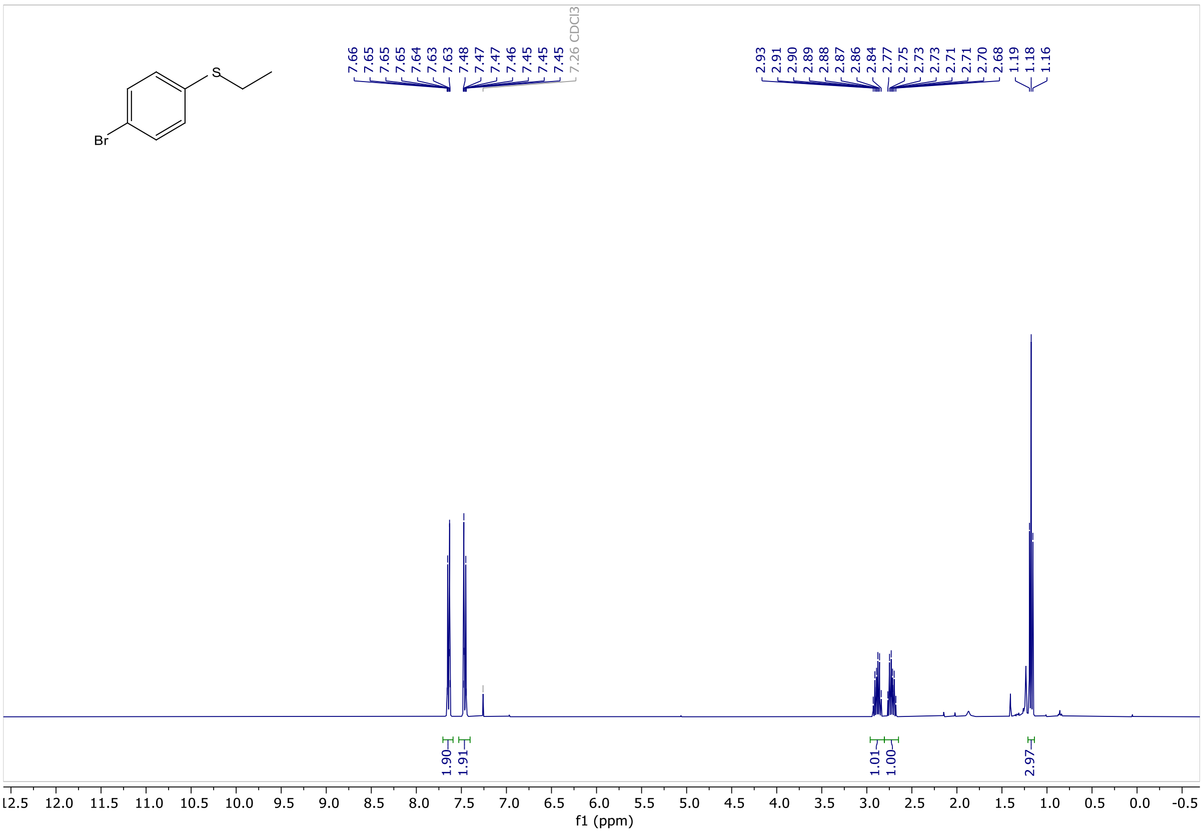

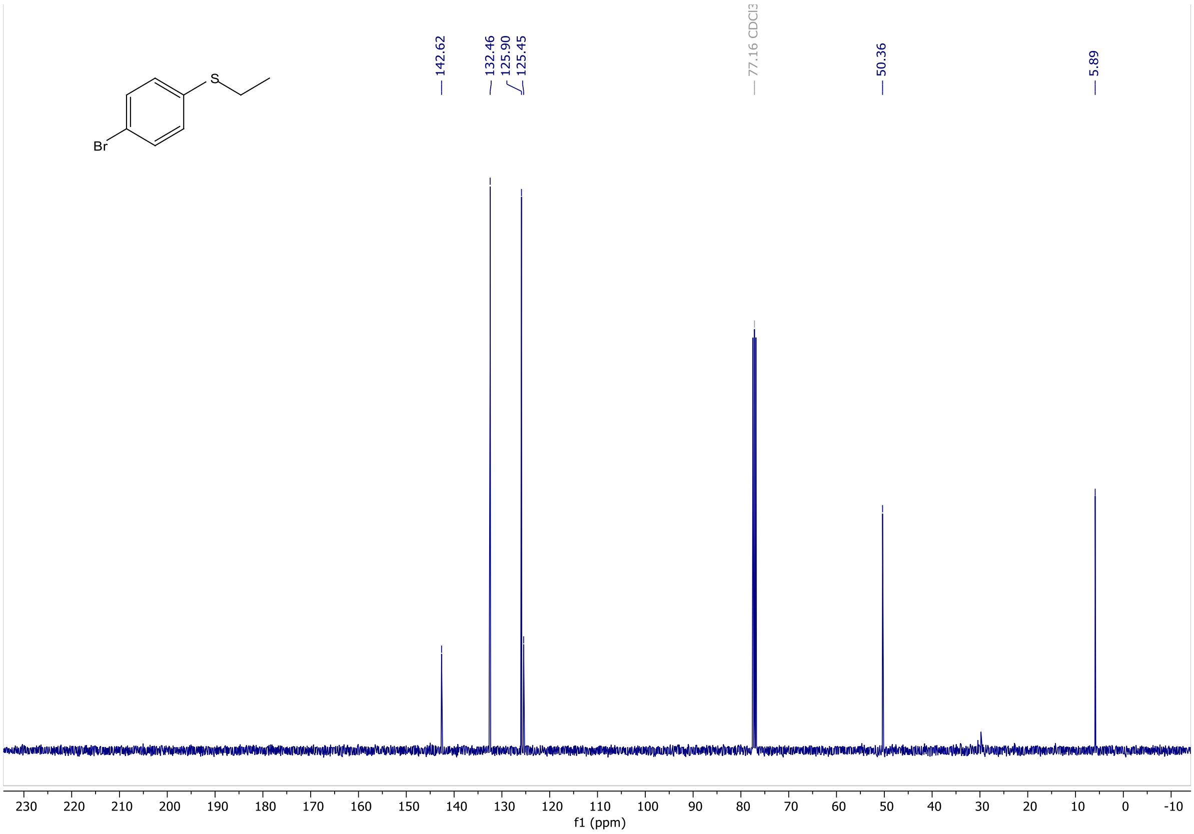

2-((Propylsulfinyl)methyl)pyridine (5a)

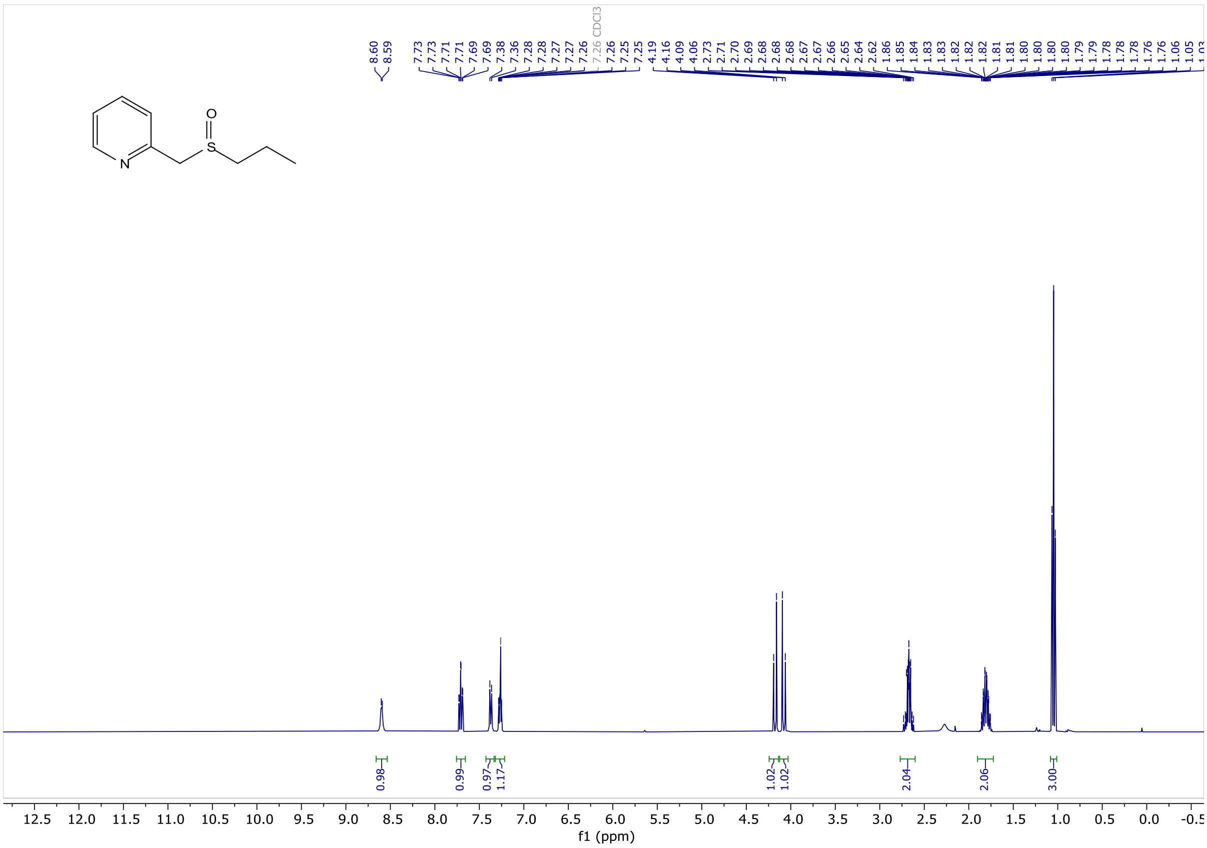

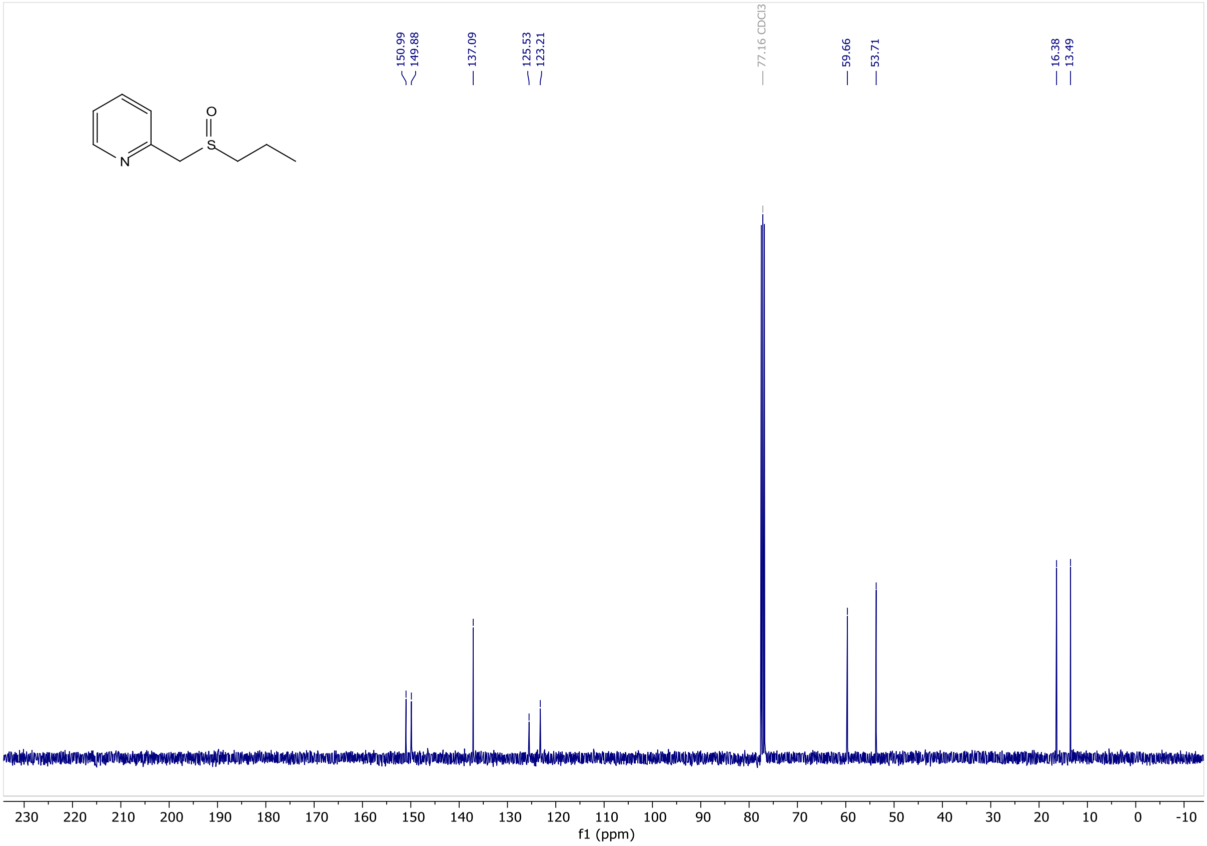

2-((Ethylsulfinyl)methyl)pyridine (5b)

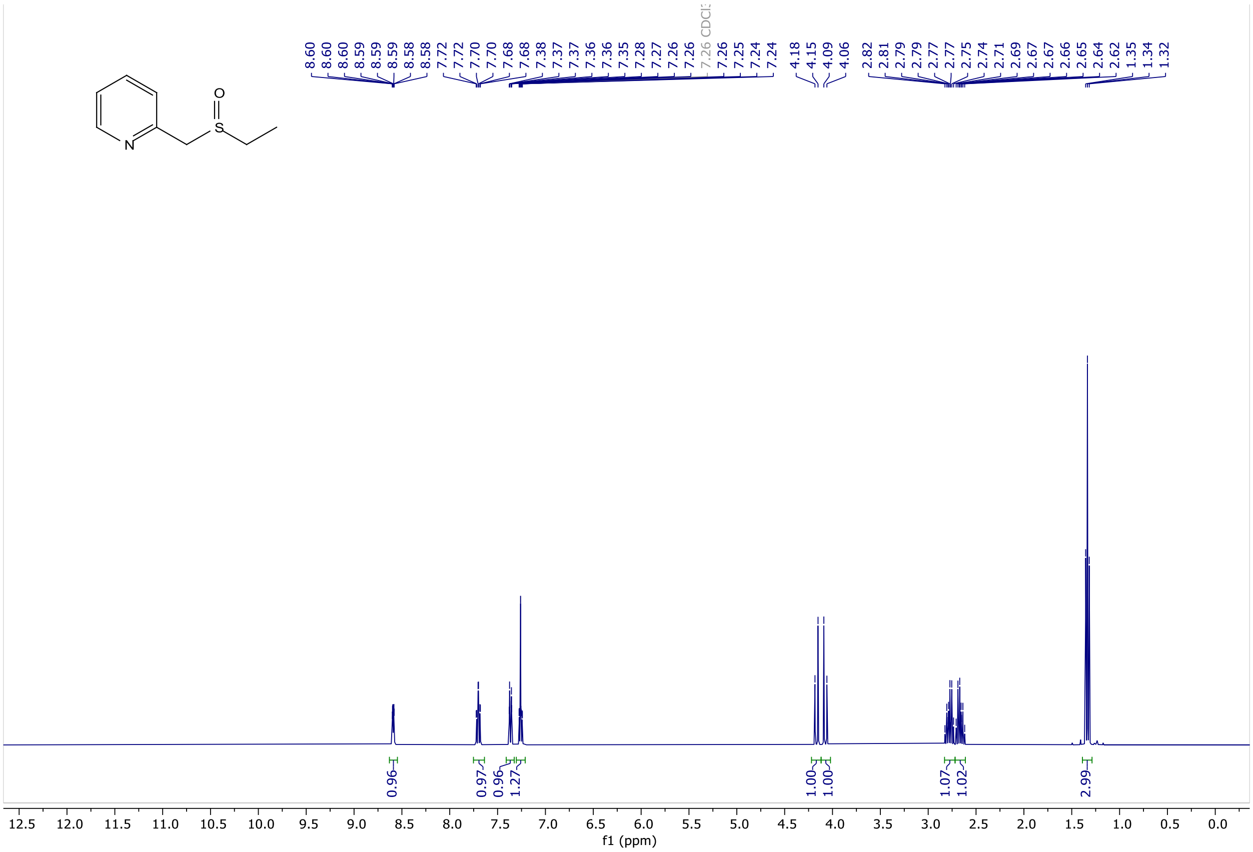

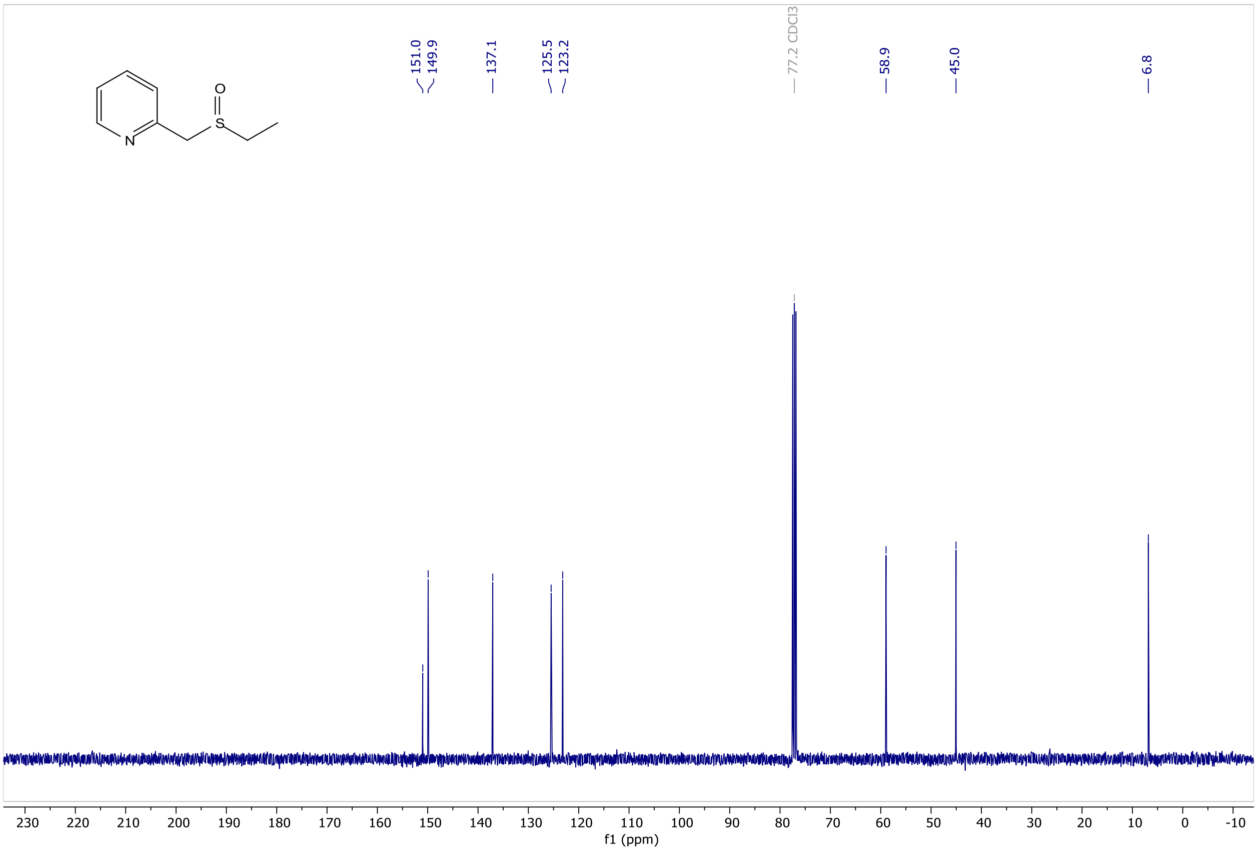

2-((Isobutylsulfinyl)methyl)pyridine (5c)

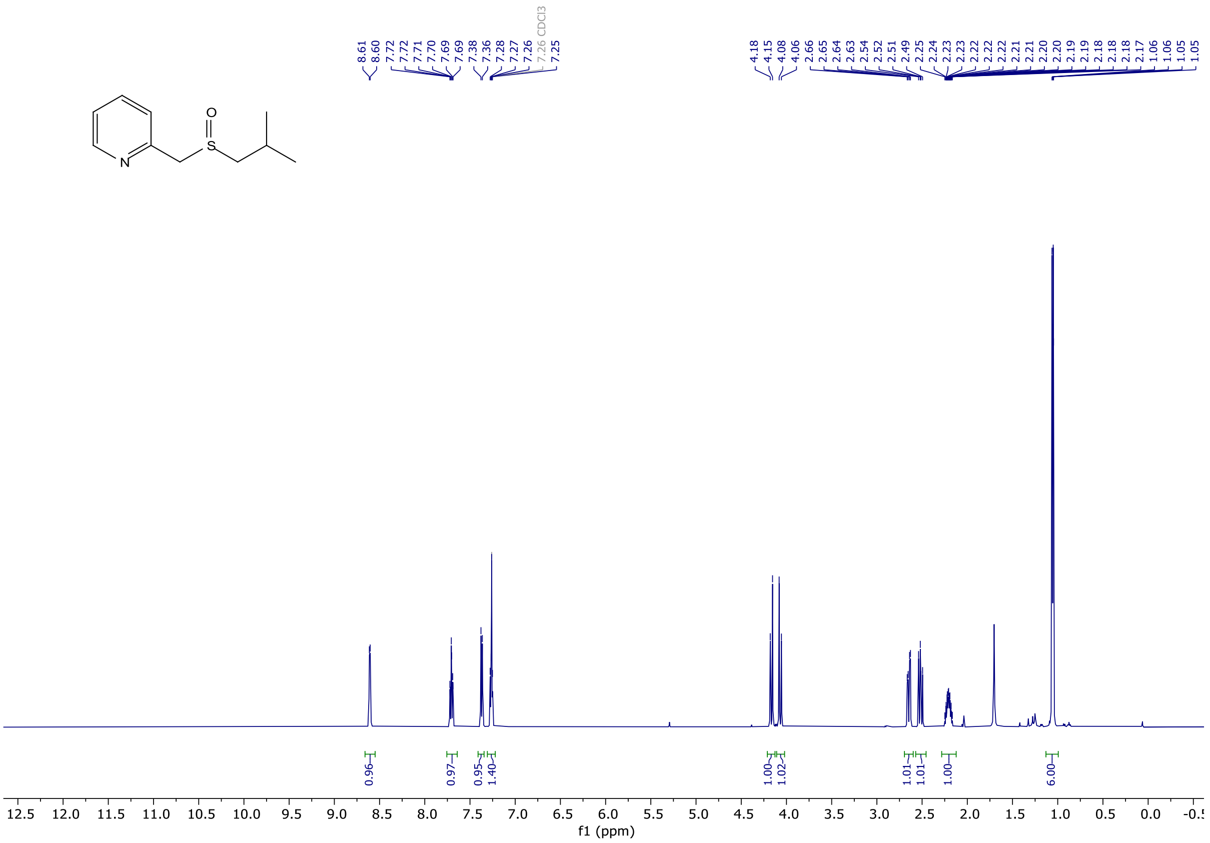

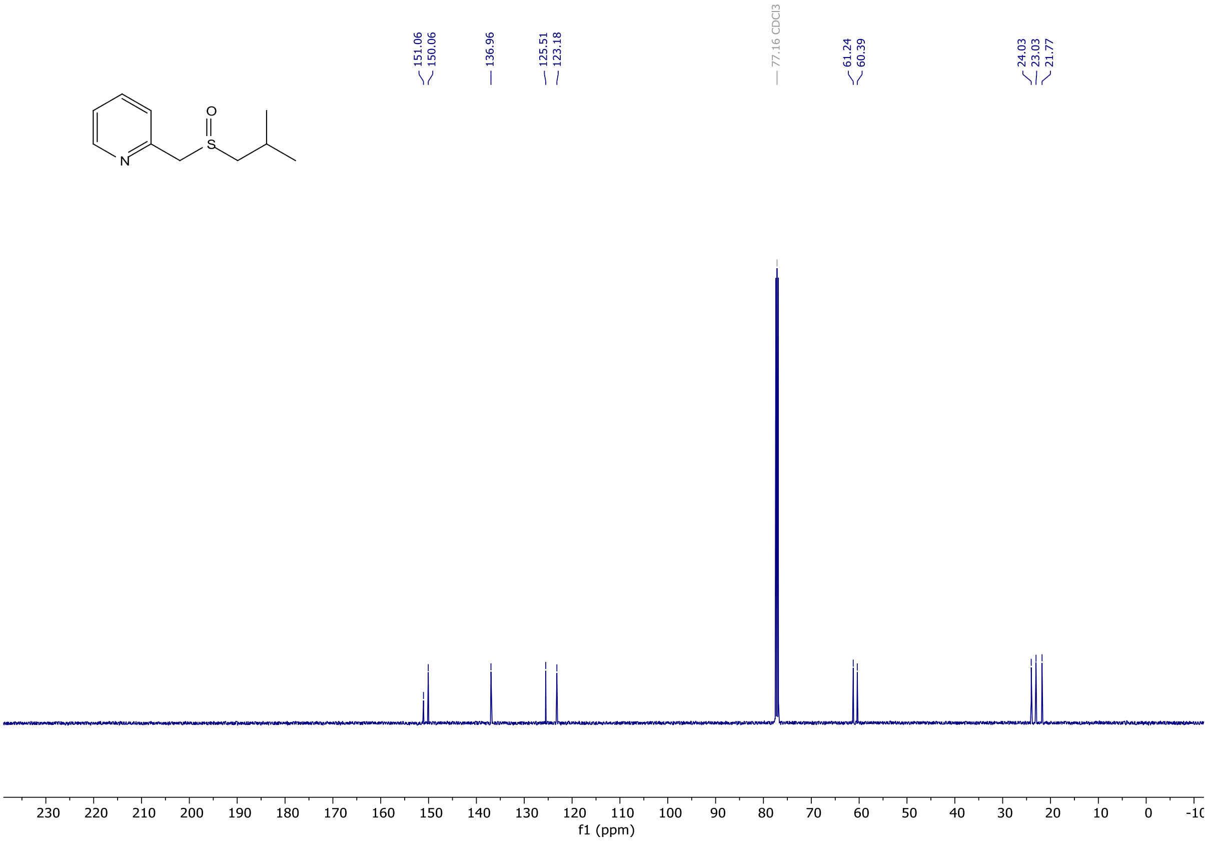

2-((Benzylsulfinyl)methyl)pyridine (5d)

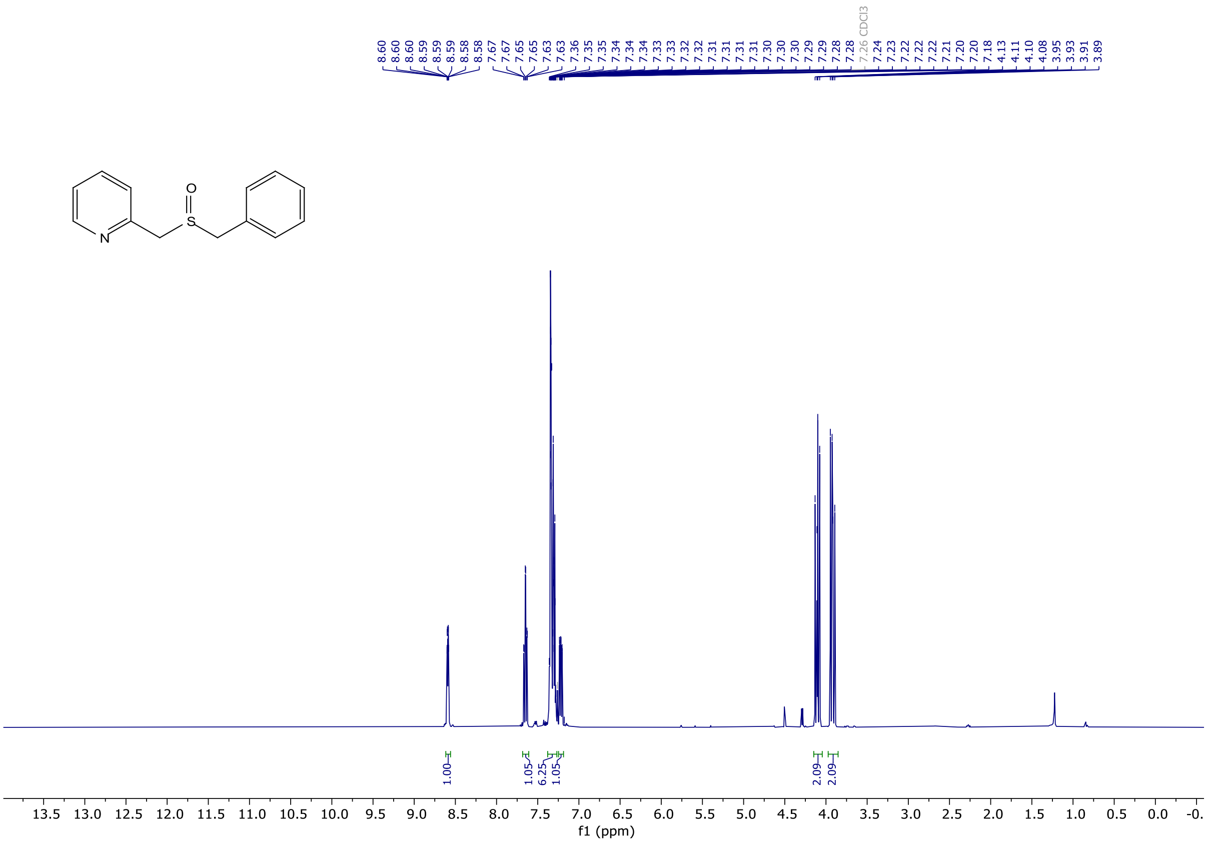

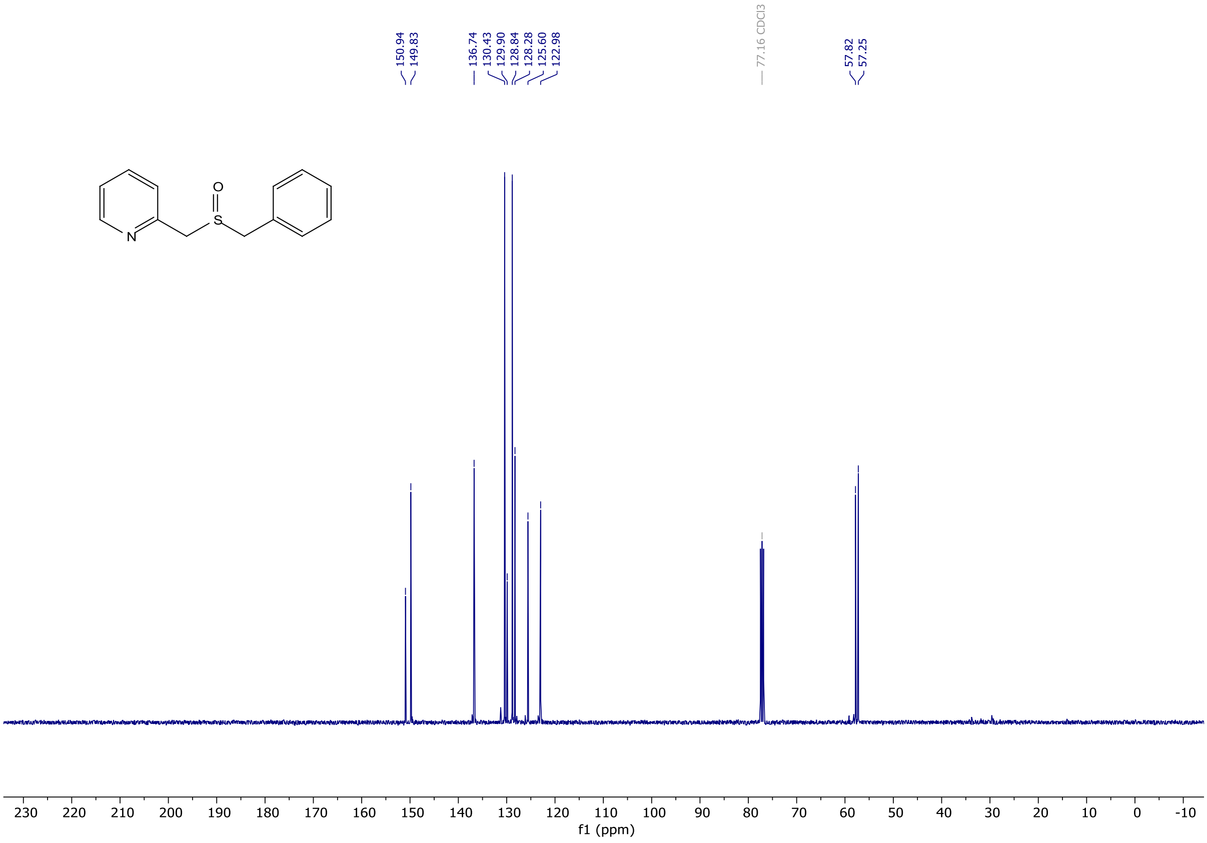

2-(Propylsulfinyl)pyridine (5e)

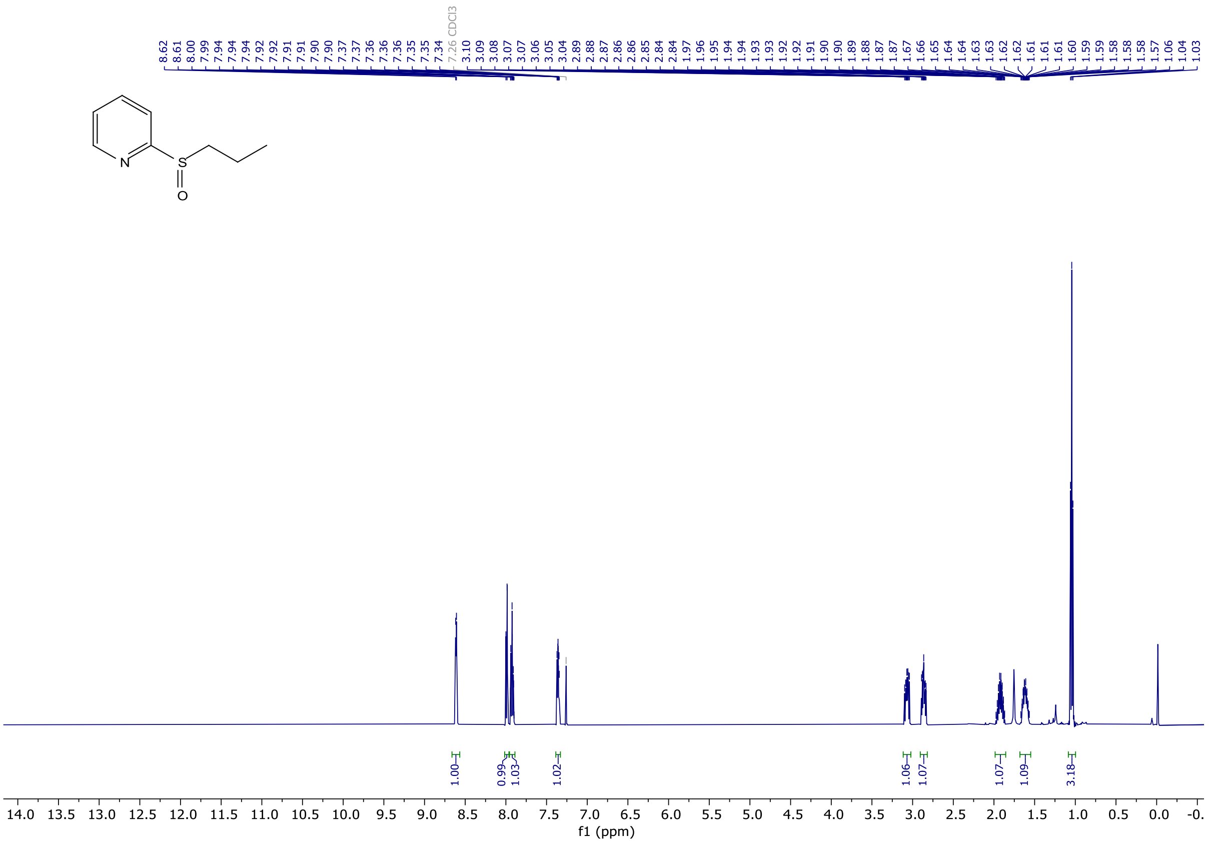

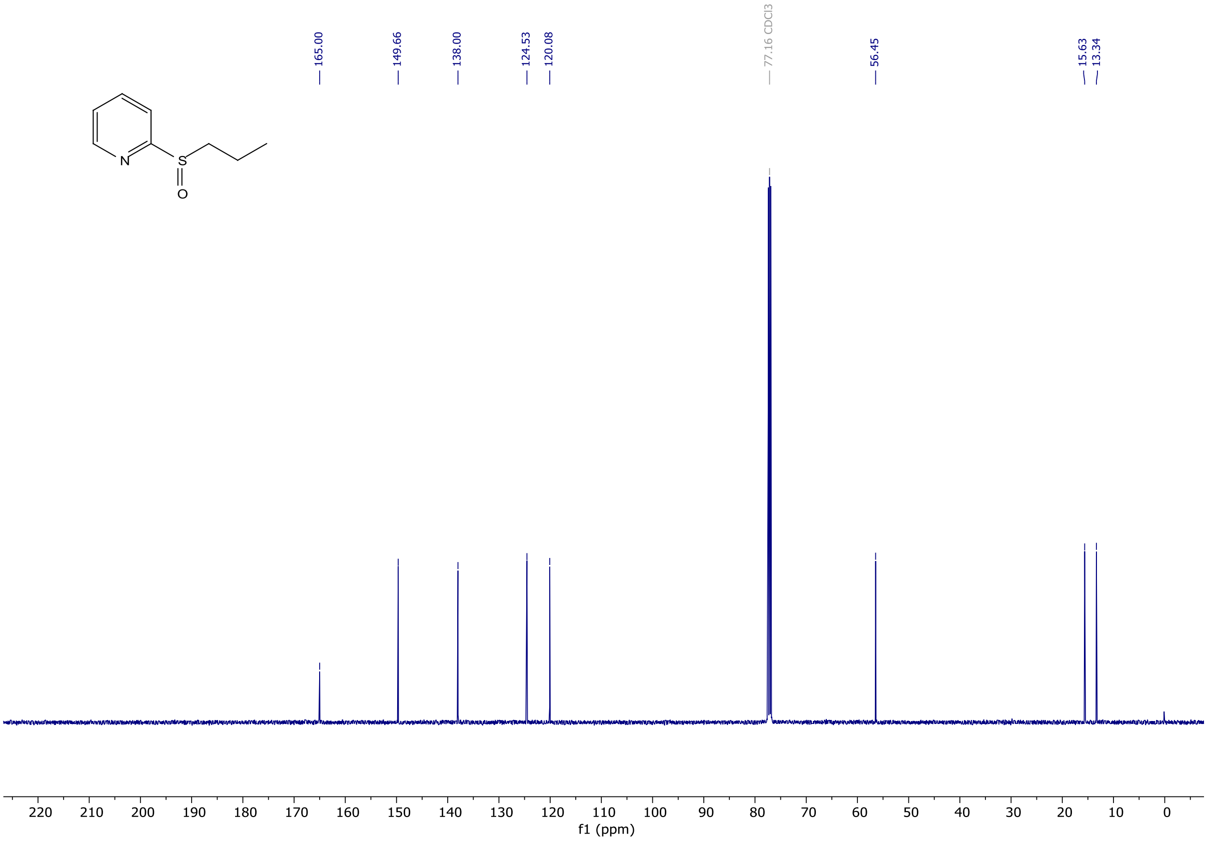

4-Methyl-2-(propylsulfinyl)pyridine (5f)

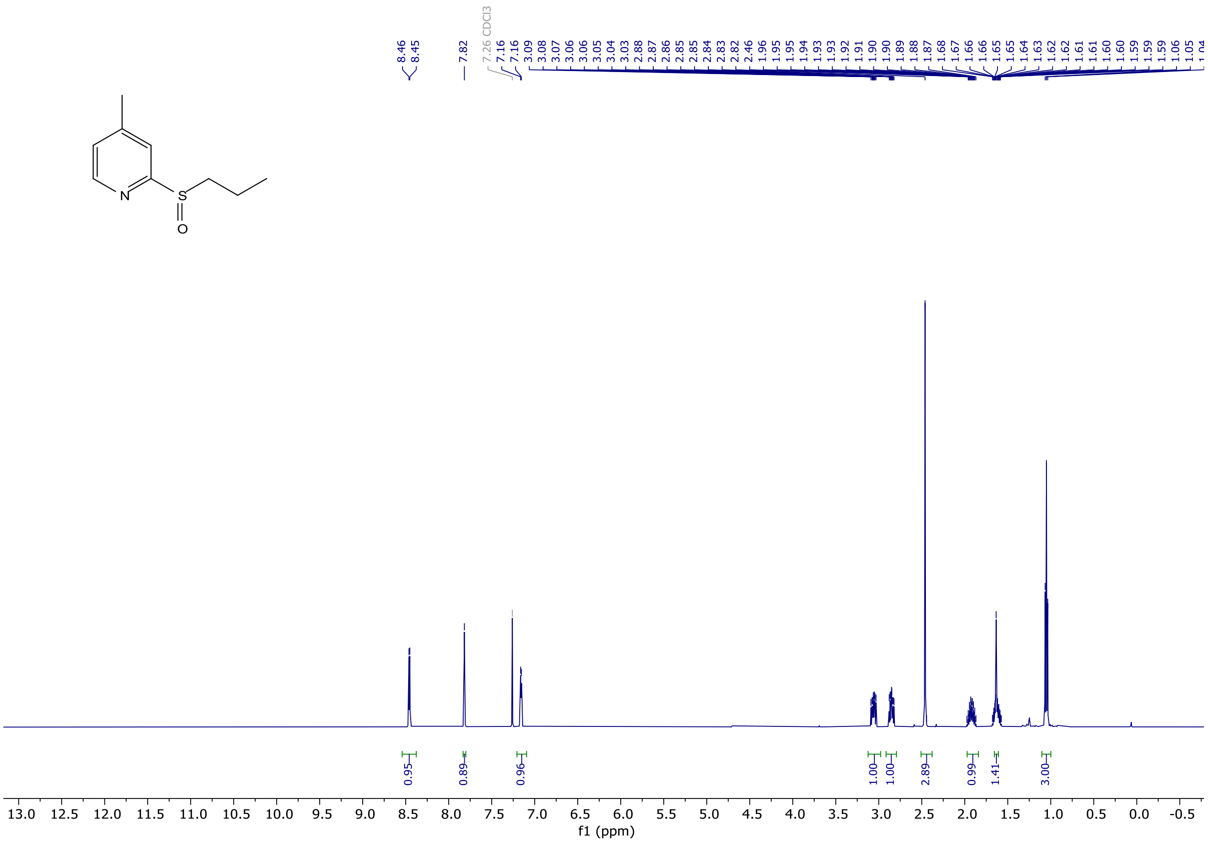

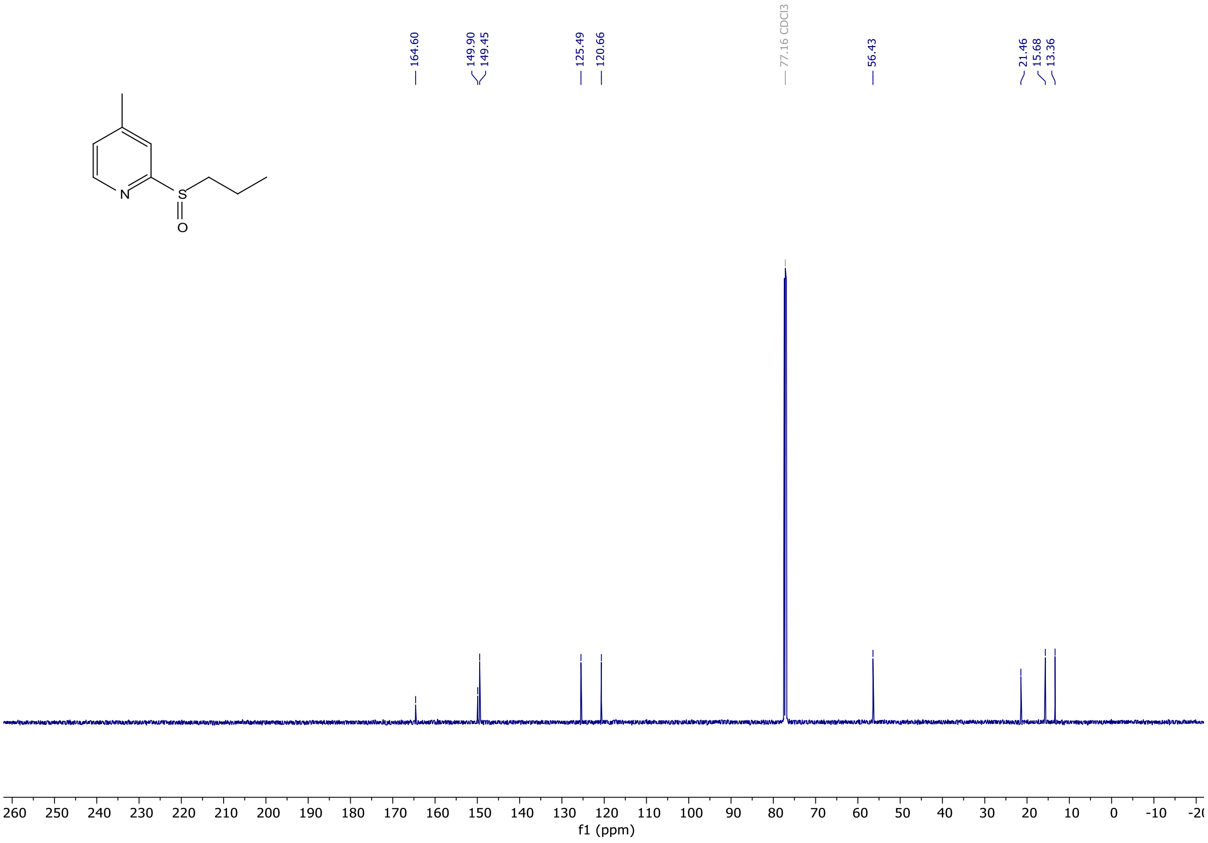

1-Methyl-2-(propylsulfinyl)-1H-imidazole (5g)

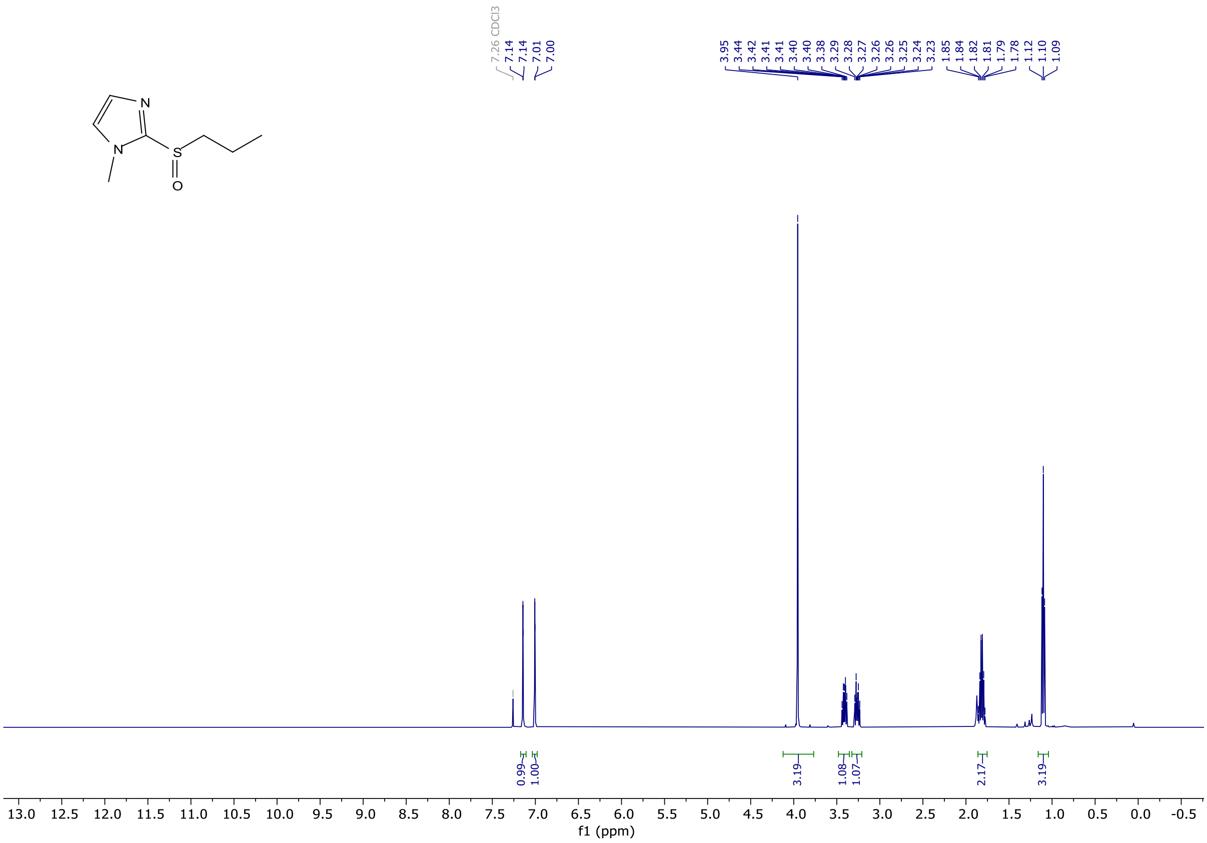

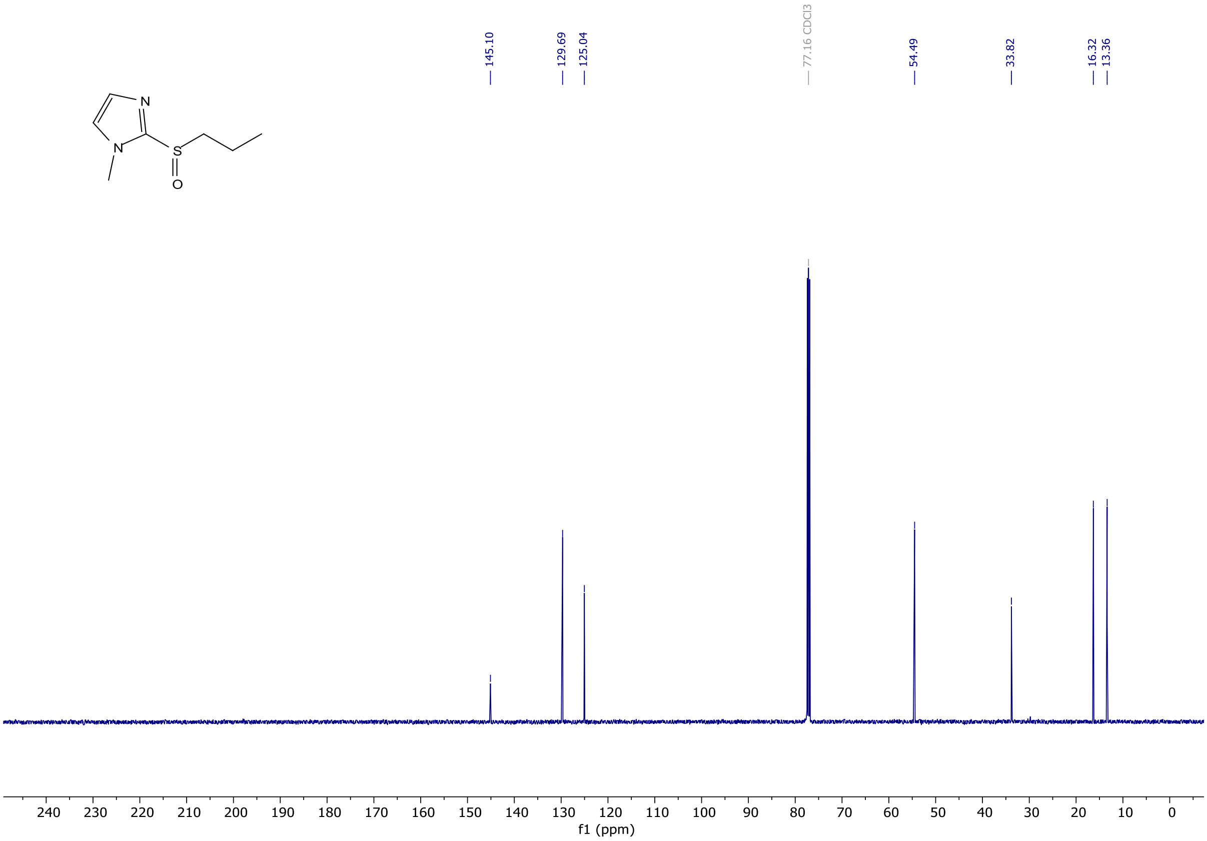

((Ethylsulfinyl)methyl)benzene (5h)

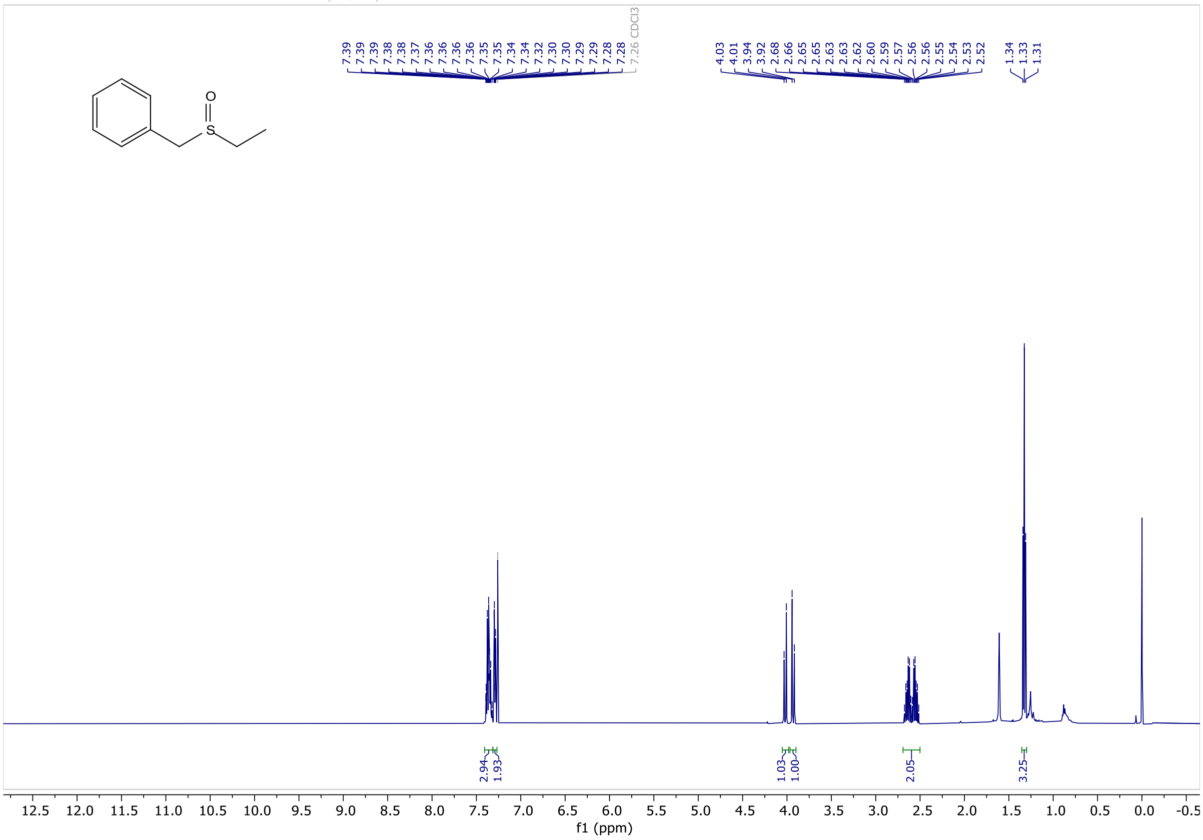

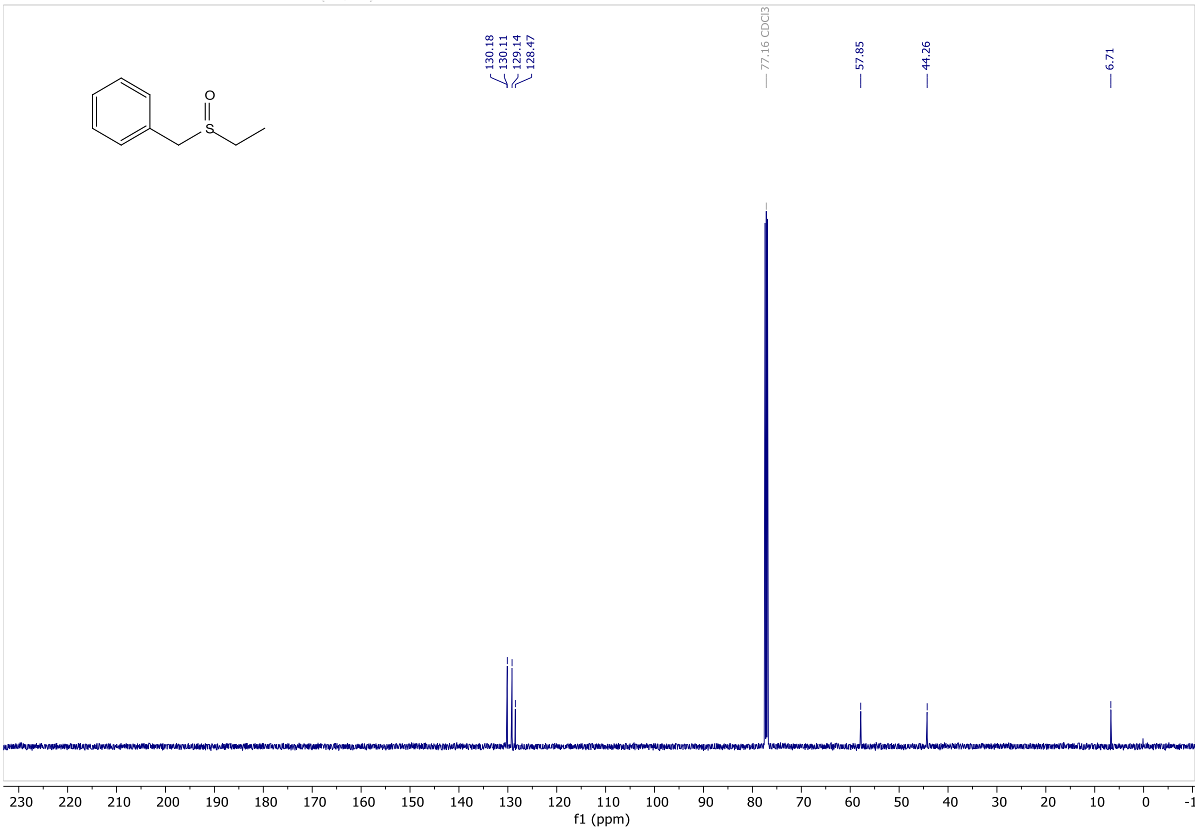

Ethyl (4-bromo)phenyl sulfoxide (5i)

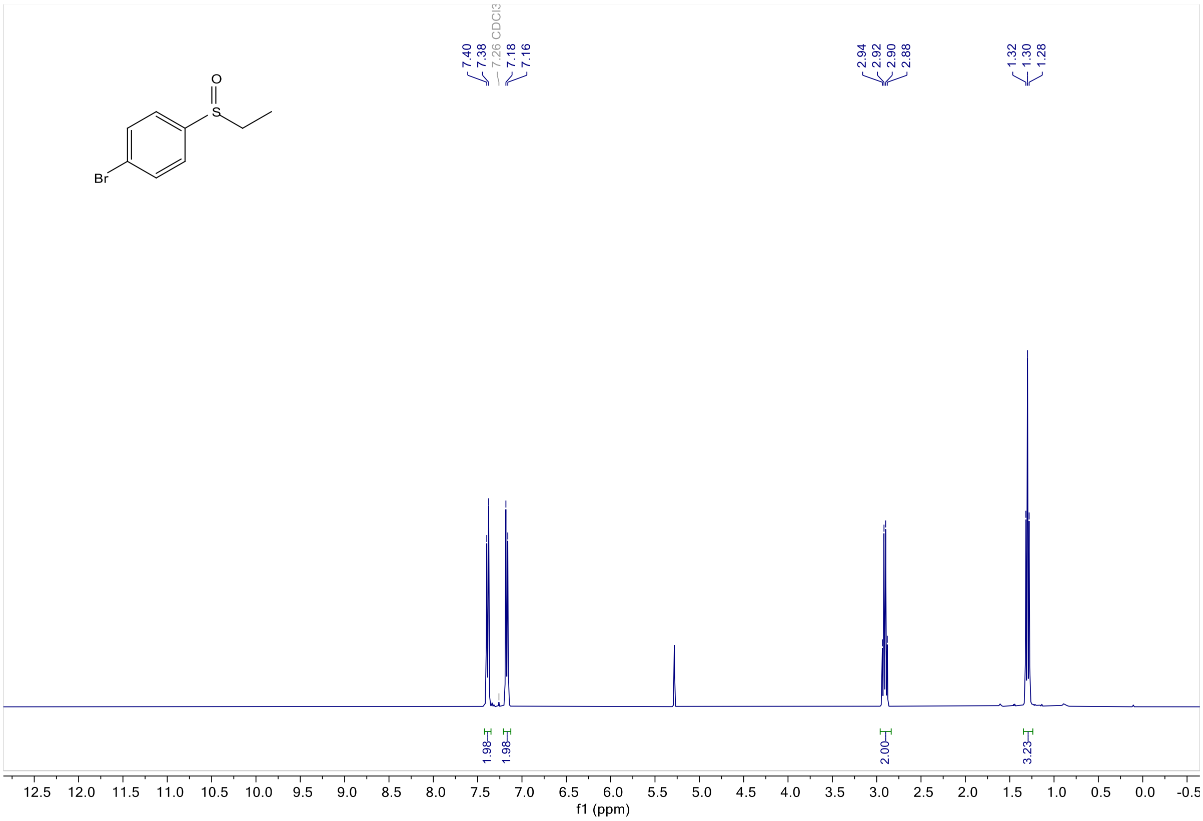

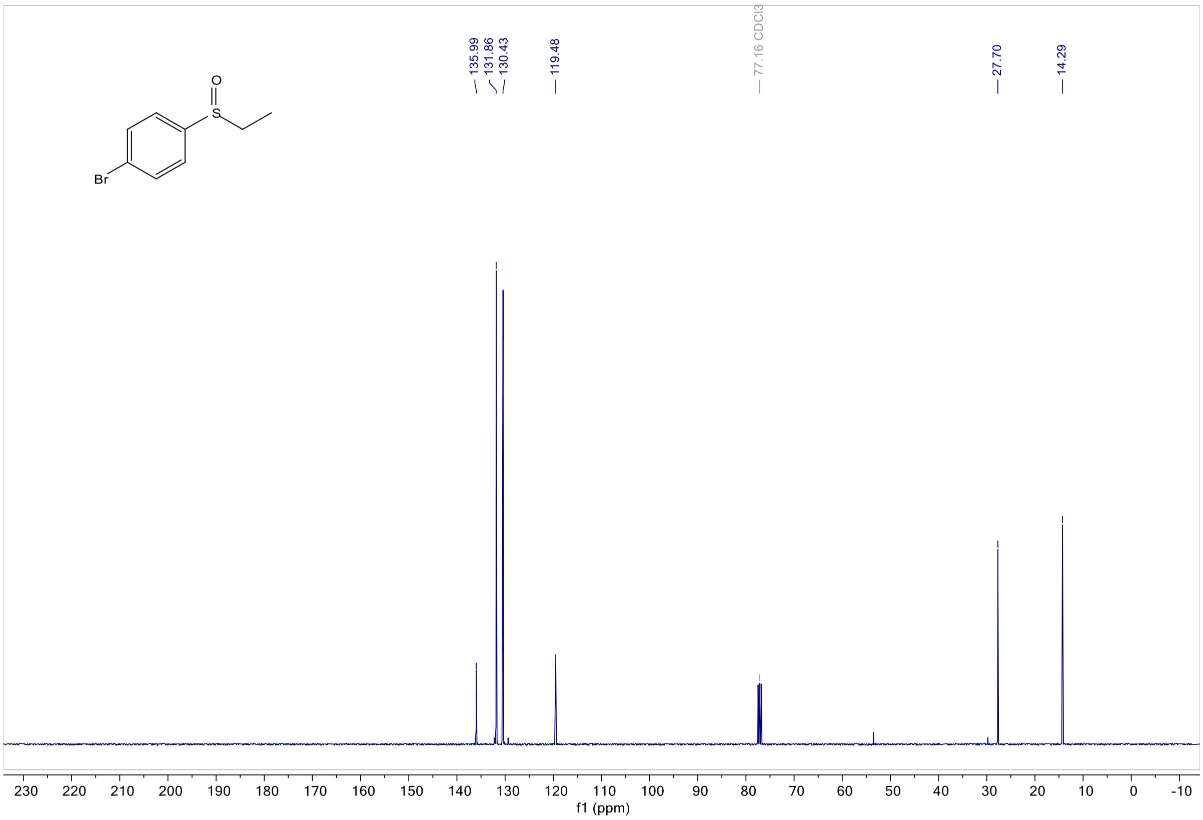

1-Chloro-3-(methylsulfinyl)benzene (5j)

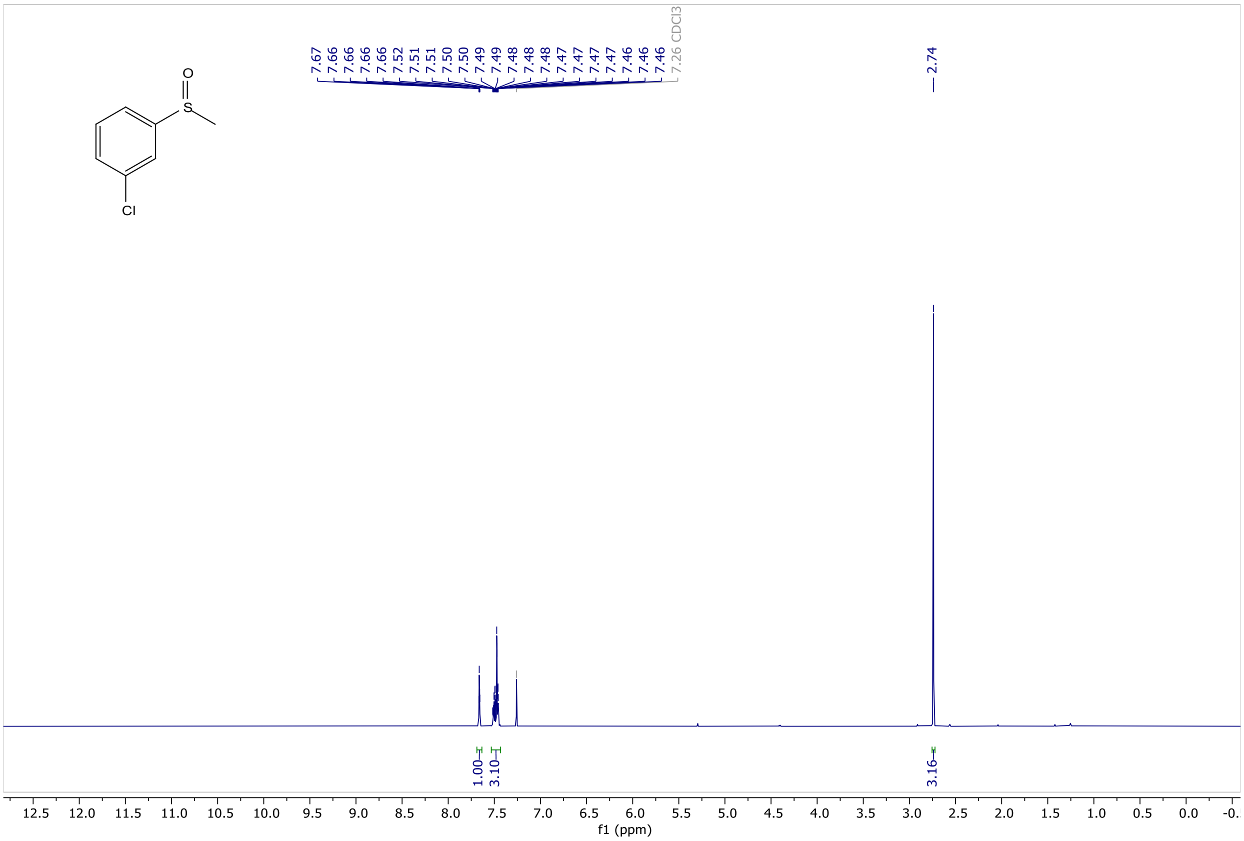

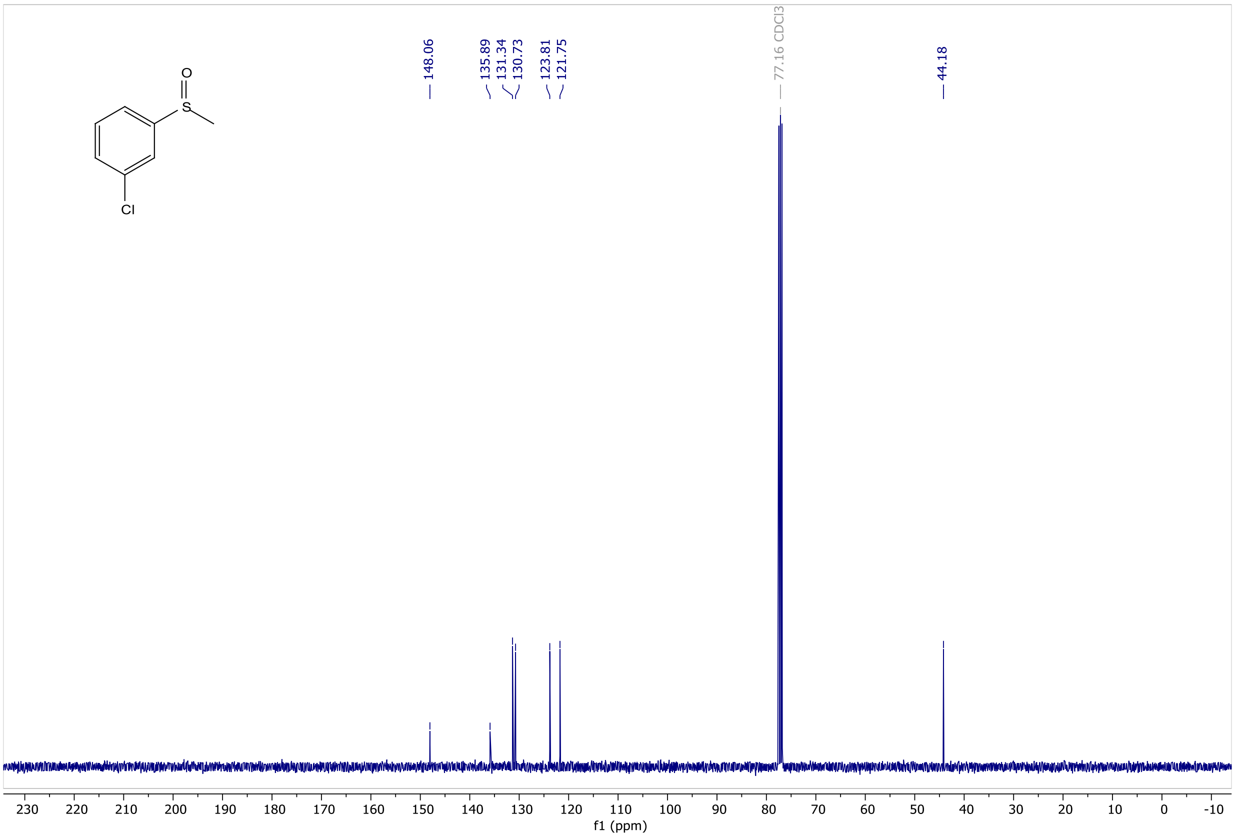

Methyl (4-fluoro)phenyl sulfoxide (5k)

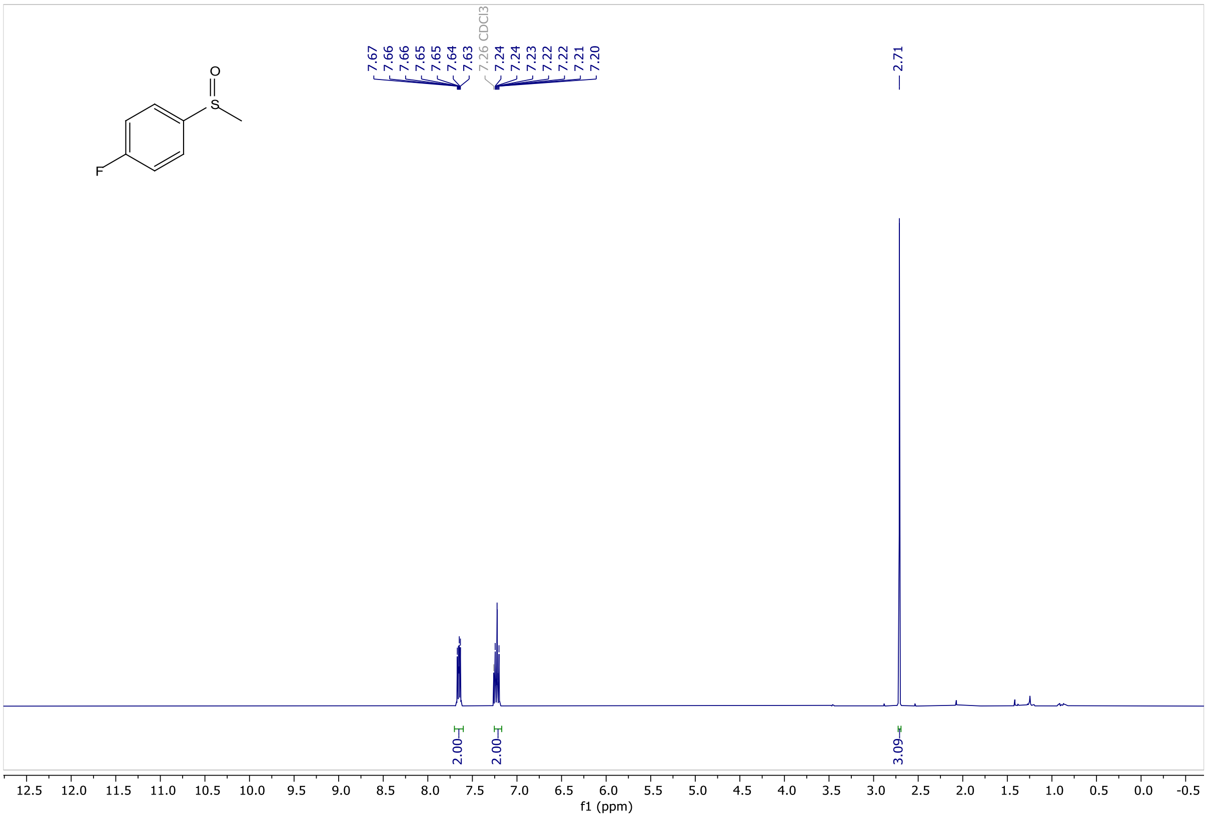

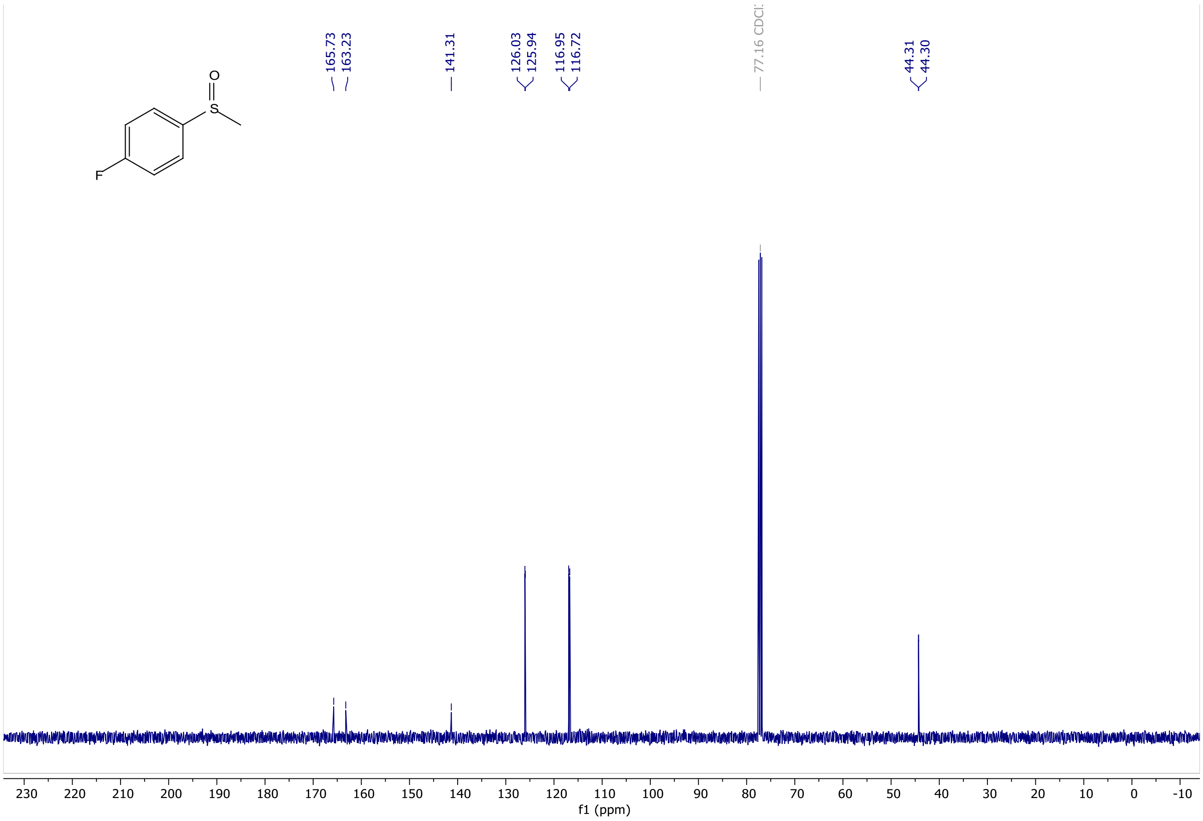

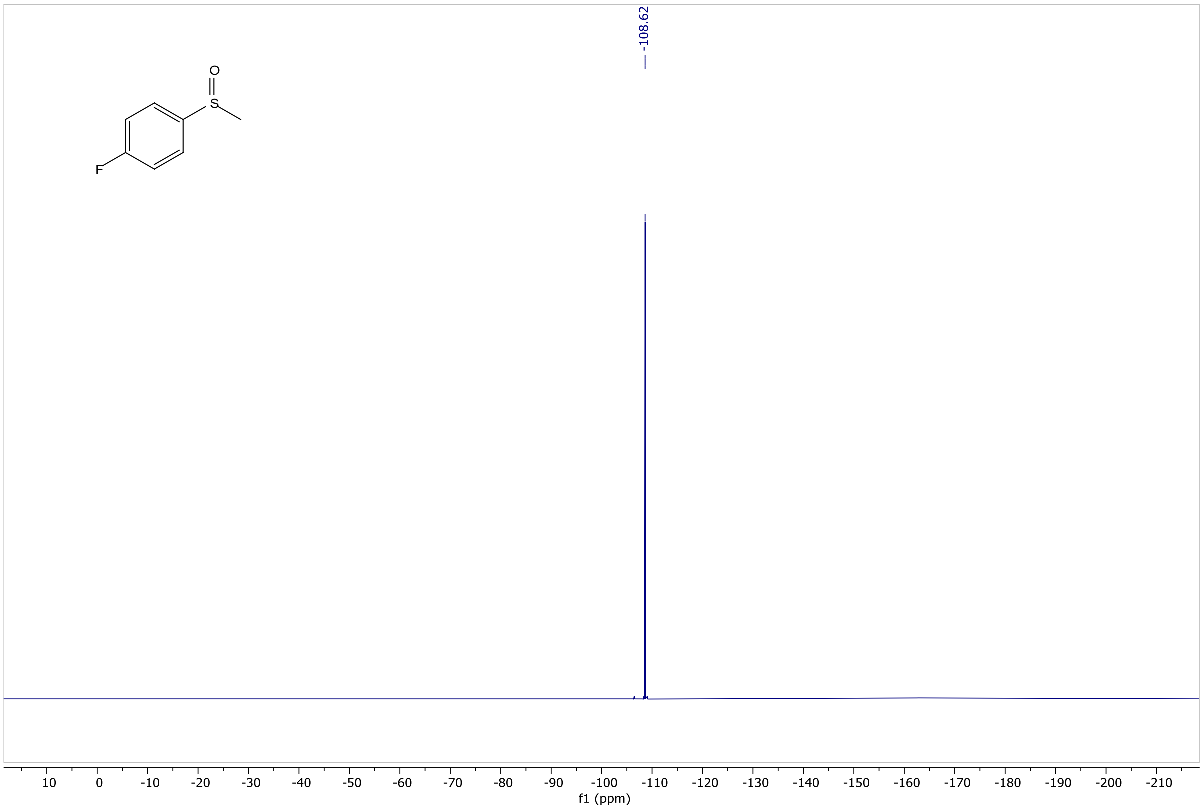

-108.62
O
S
F
10
0
-10
-20
-30
-40
-50
-60
-70
-80
-90
-100
-110
-120
-130
-140
-150
-160
-170
-180
-190
-200
-210
f1 (ppm)

1-Chloro-2-(methylsulfinyl)benzene (5n)

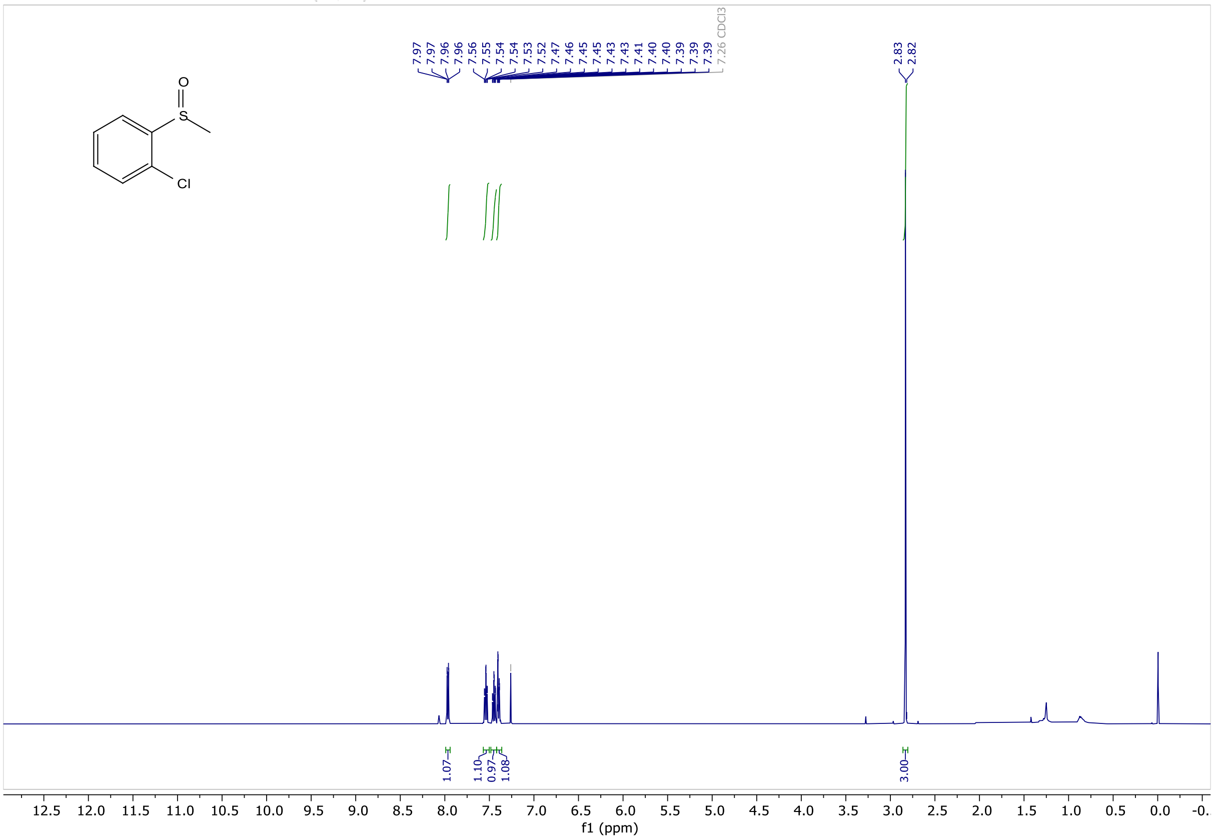

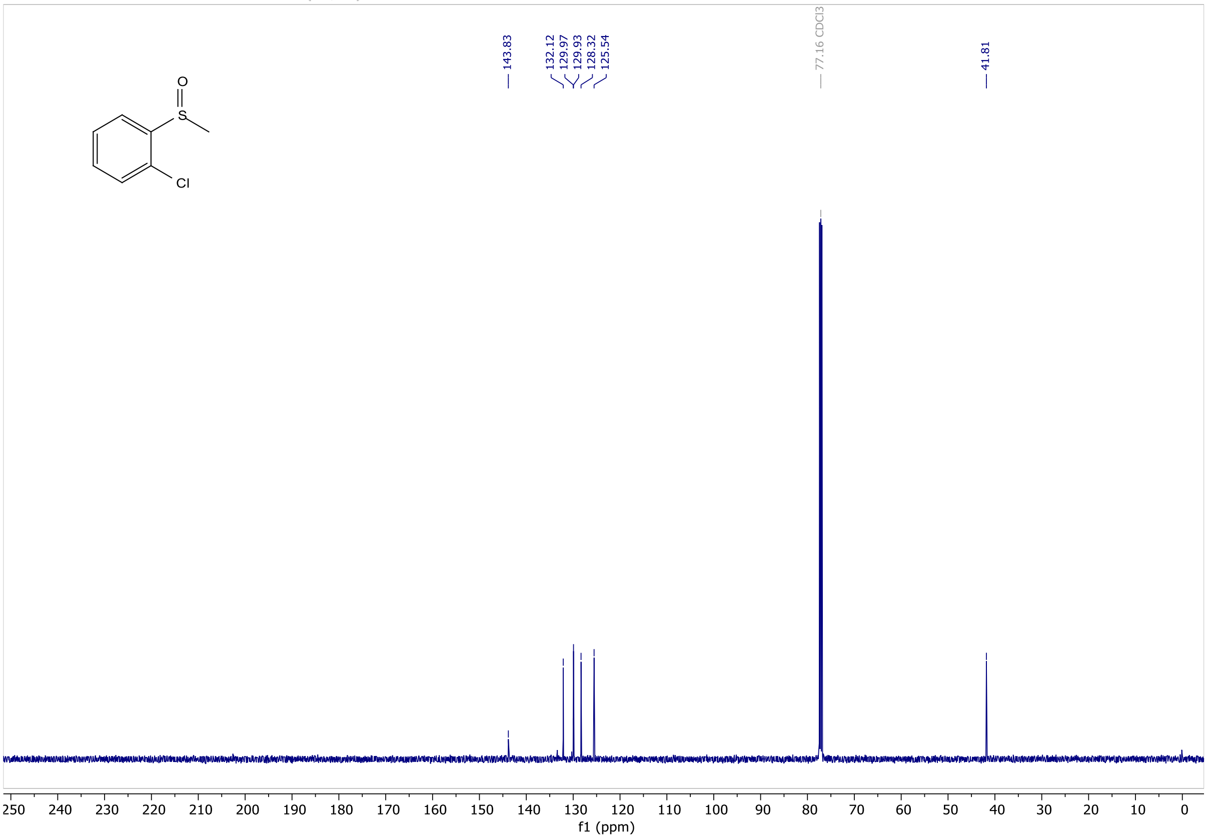

1-Chloro-4-(methylsulfinyl)benzene (5o)

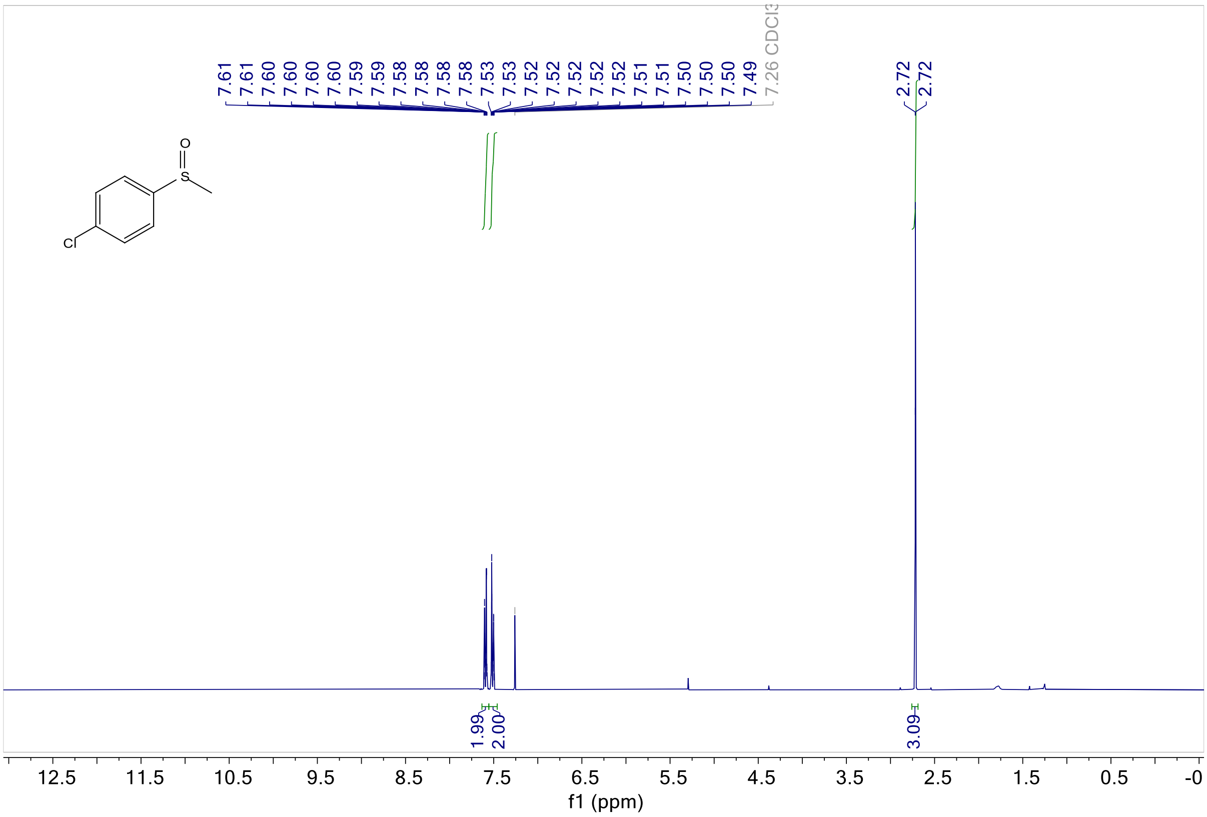

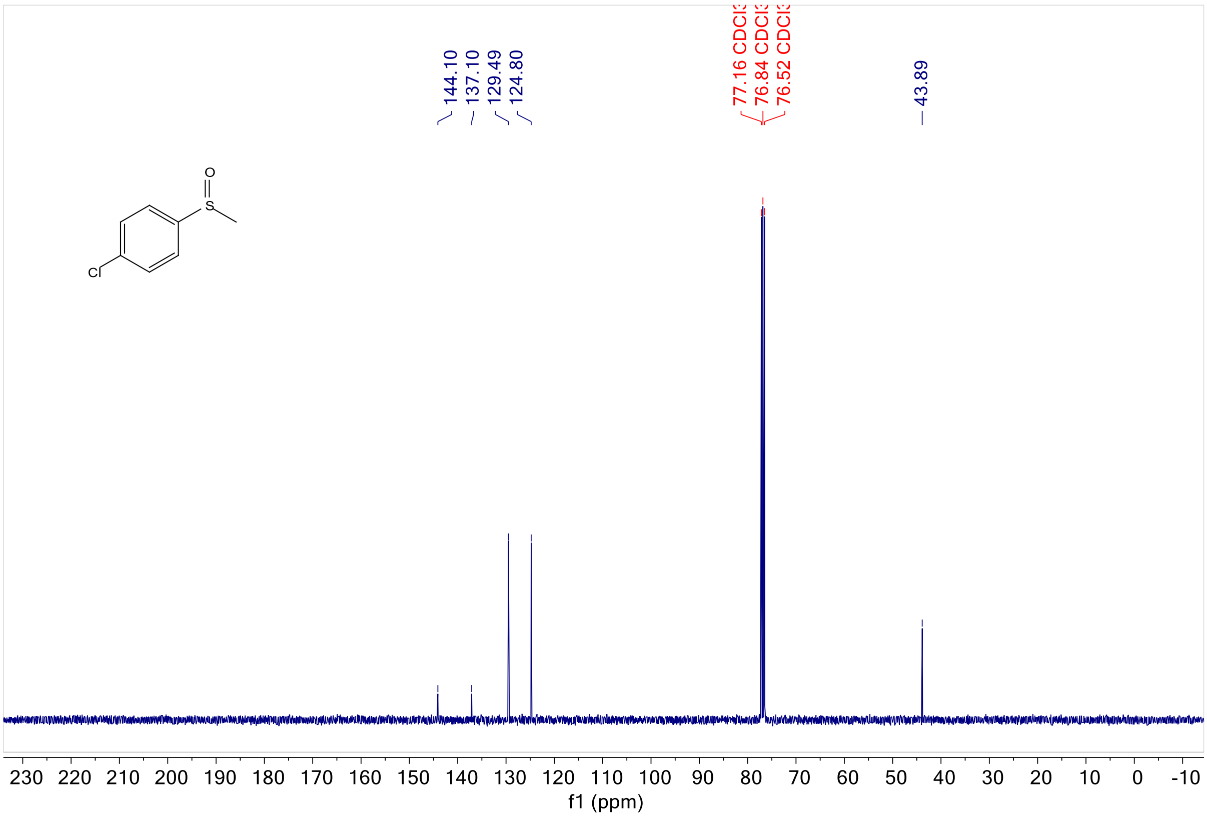

2-((Propylsulfonyl)methyl)pyridine (6a)

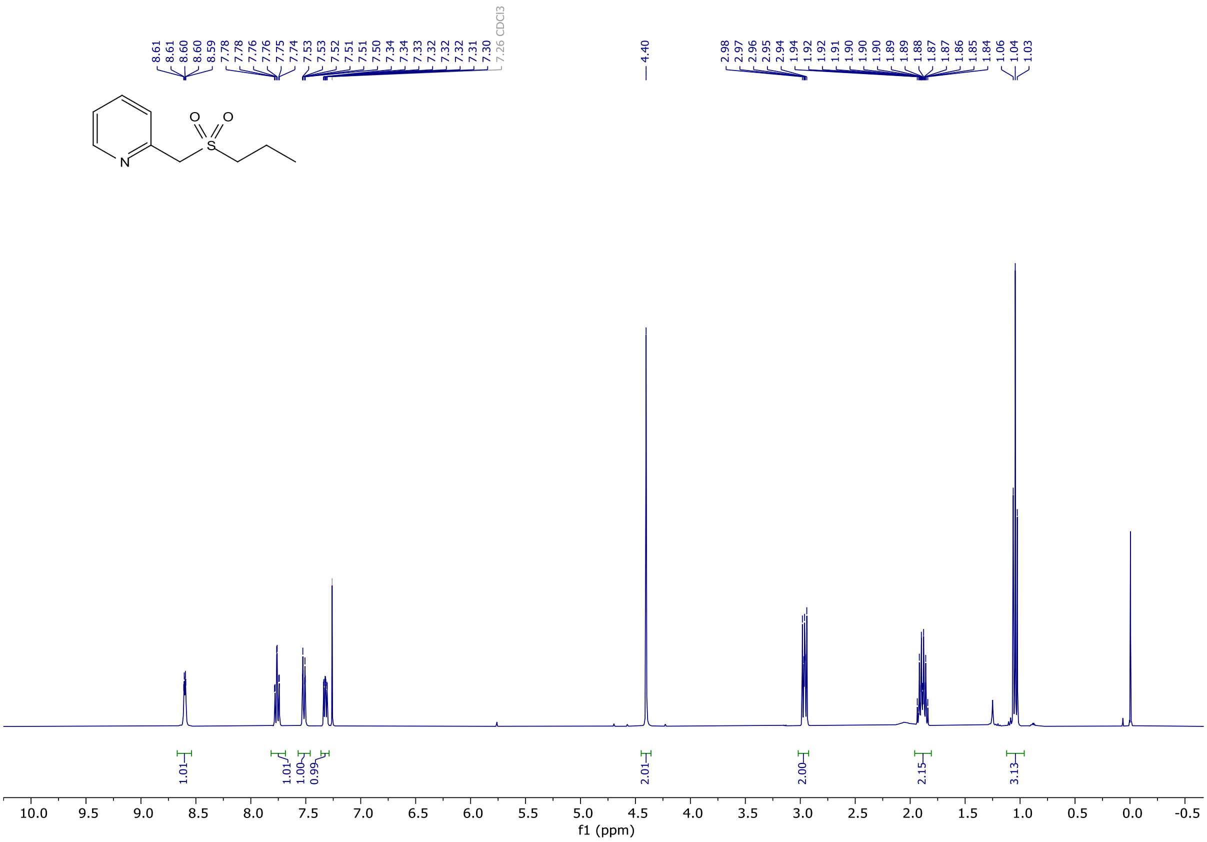

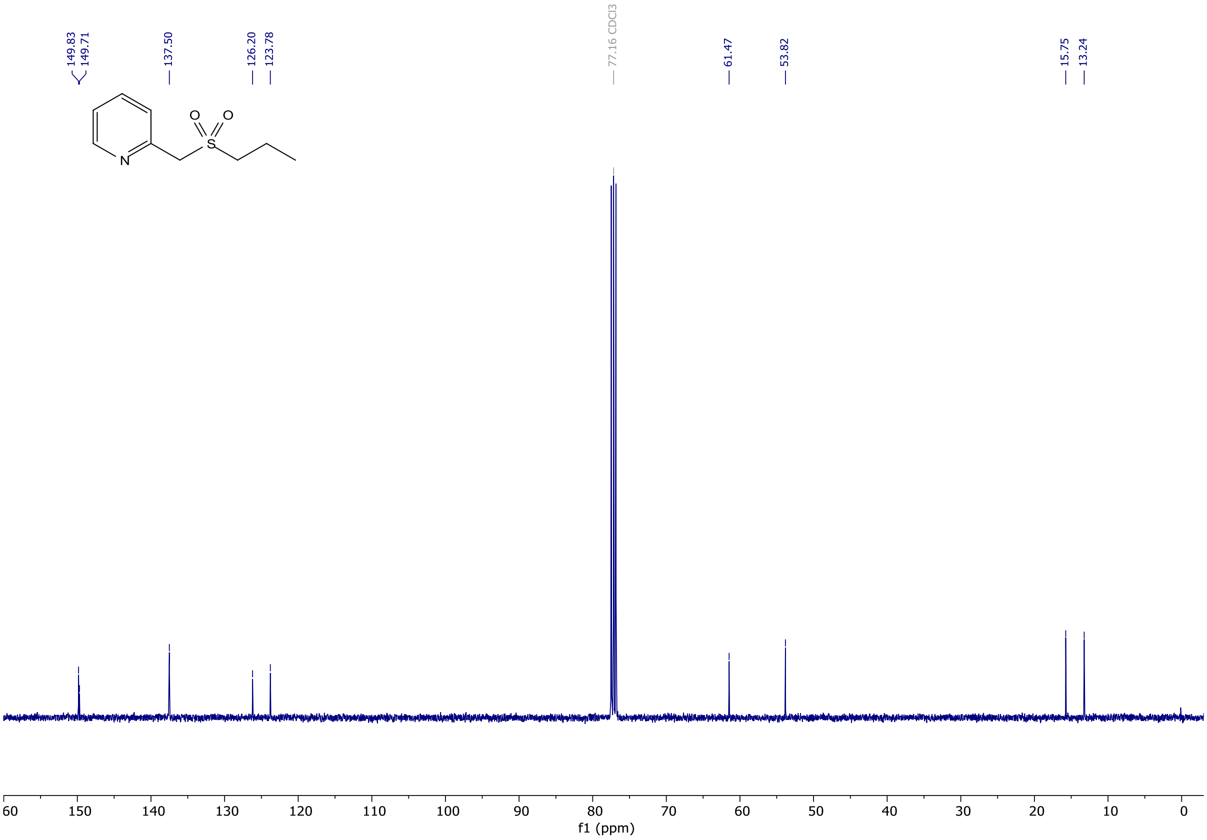

2-((Ethylsulfonyl)methyl)pyridine (6b)

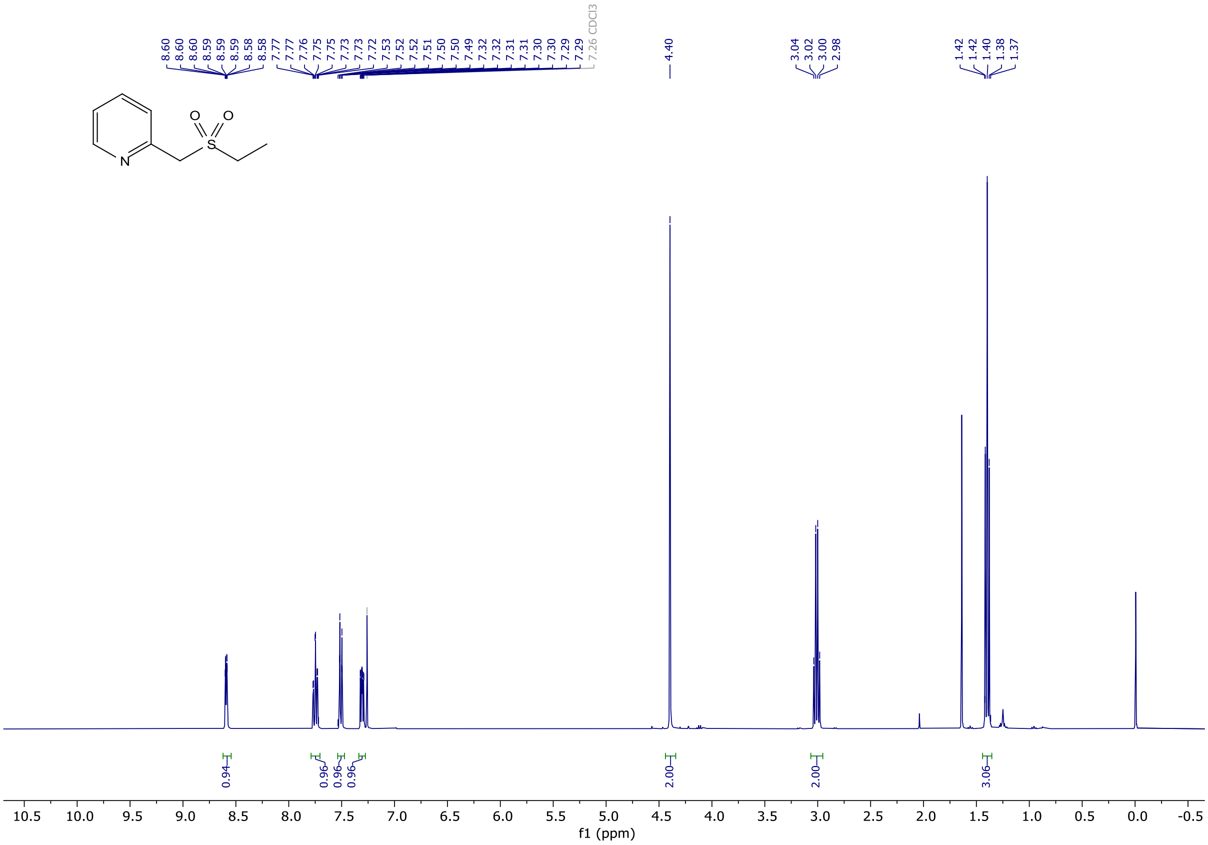

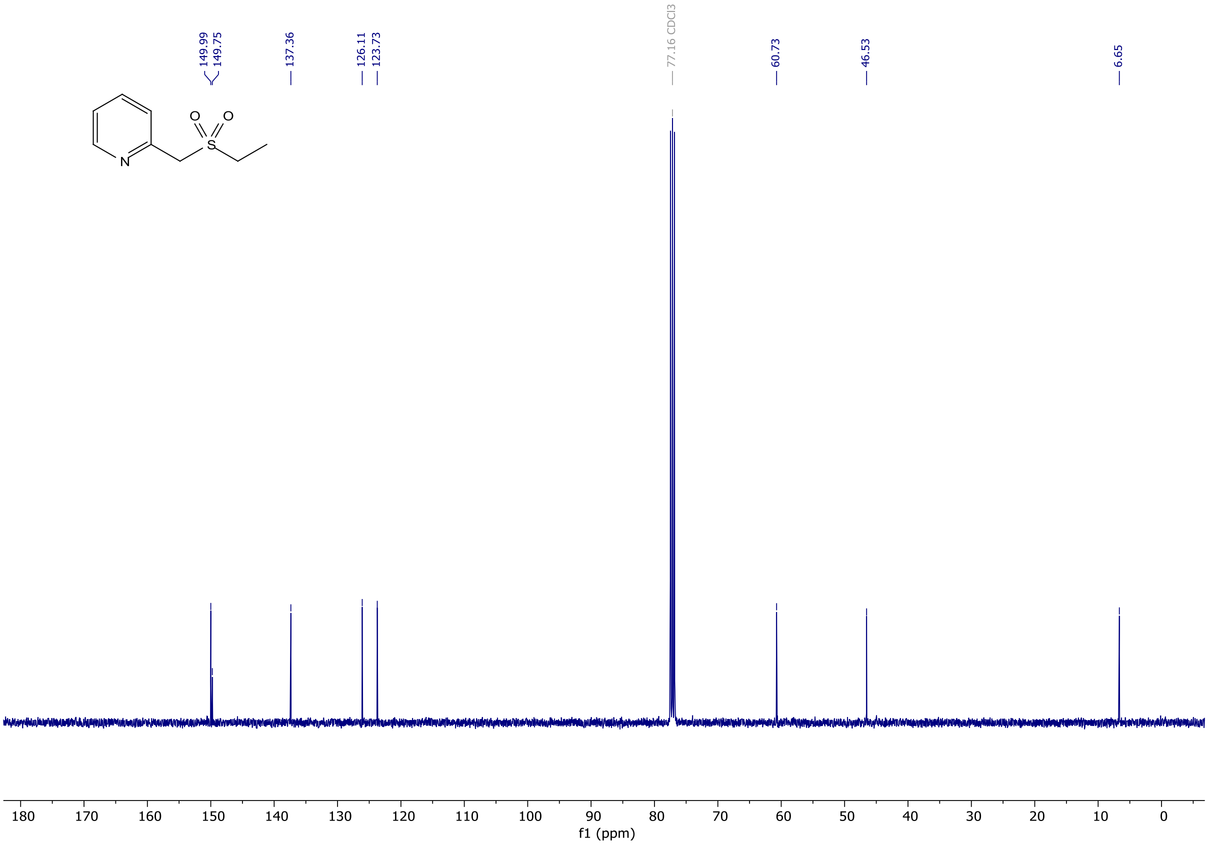

((Ethylsulfonyl)methyl)benzene (6h)

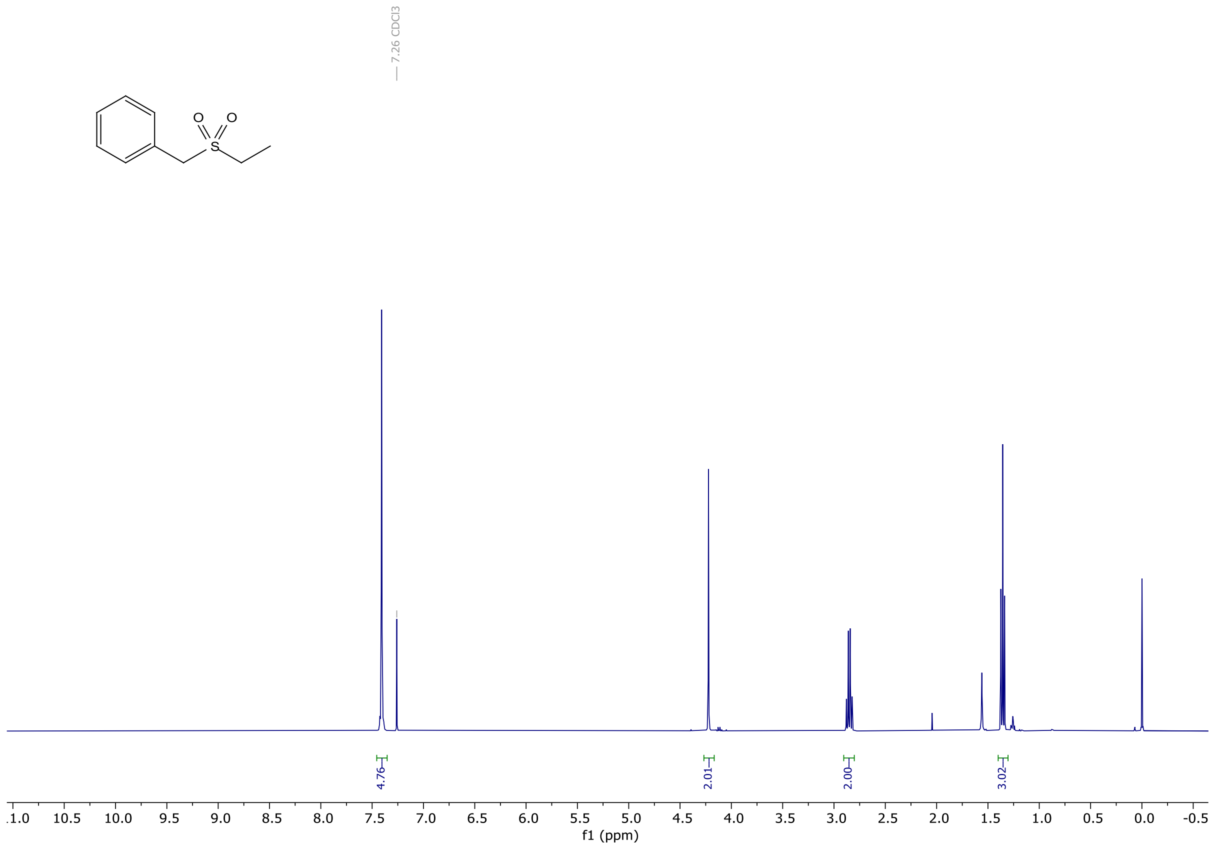

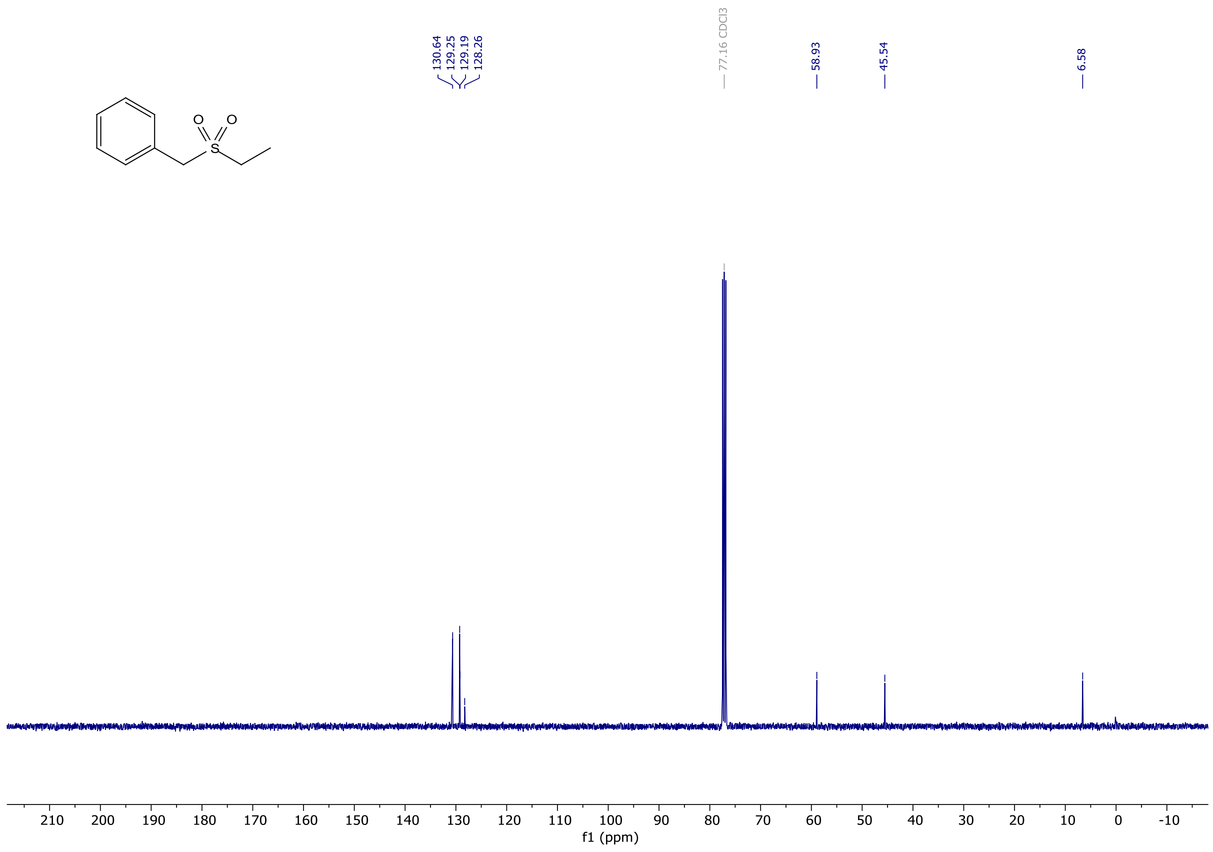

1-Methyl-3-(methylsulfonyl)benzene (6m)

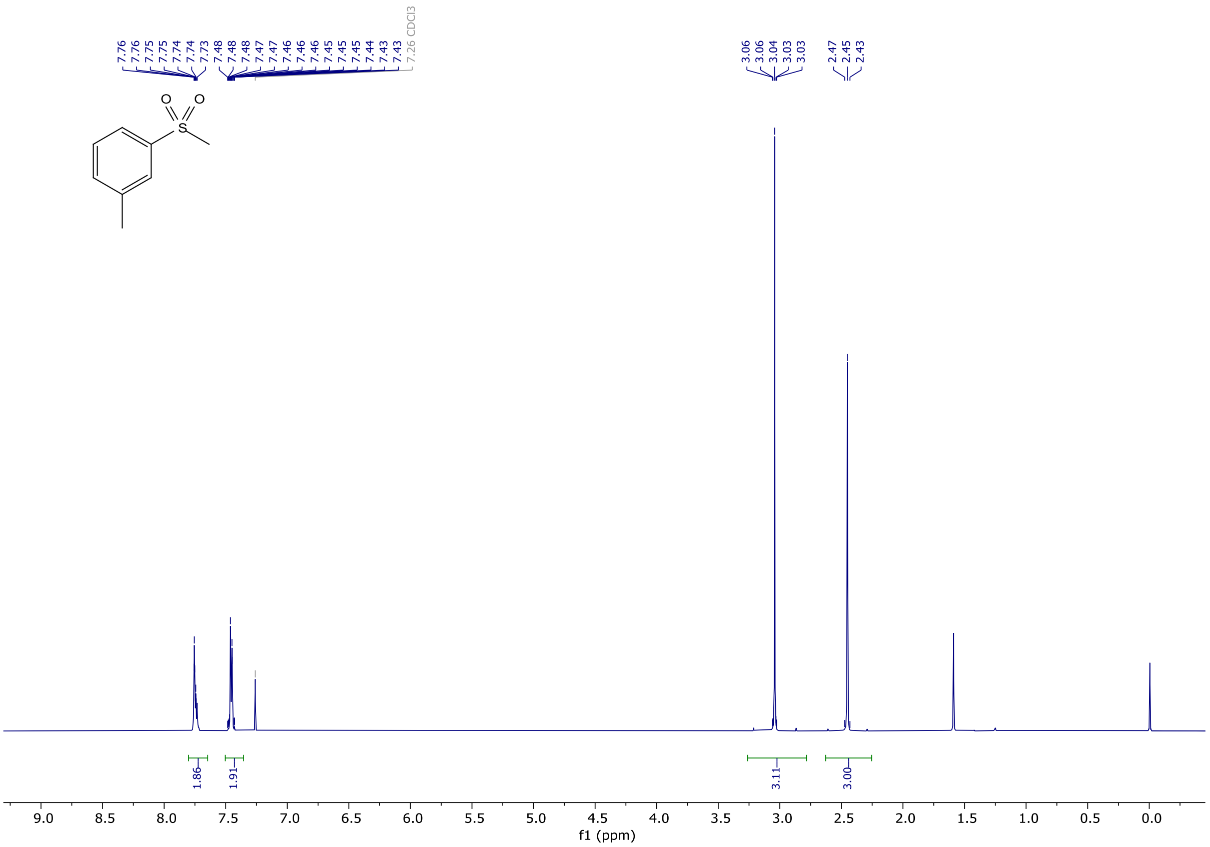

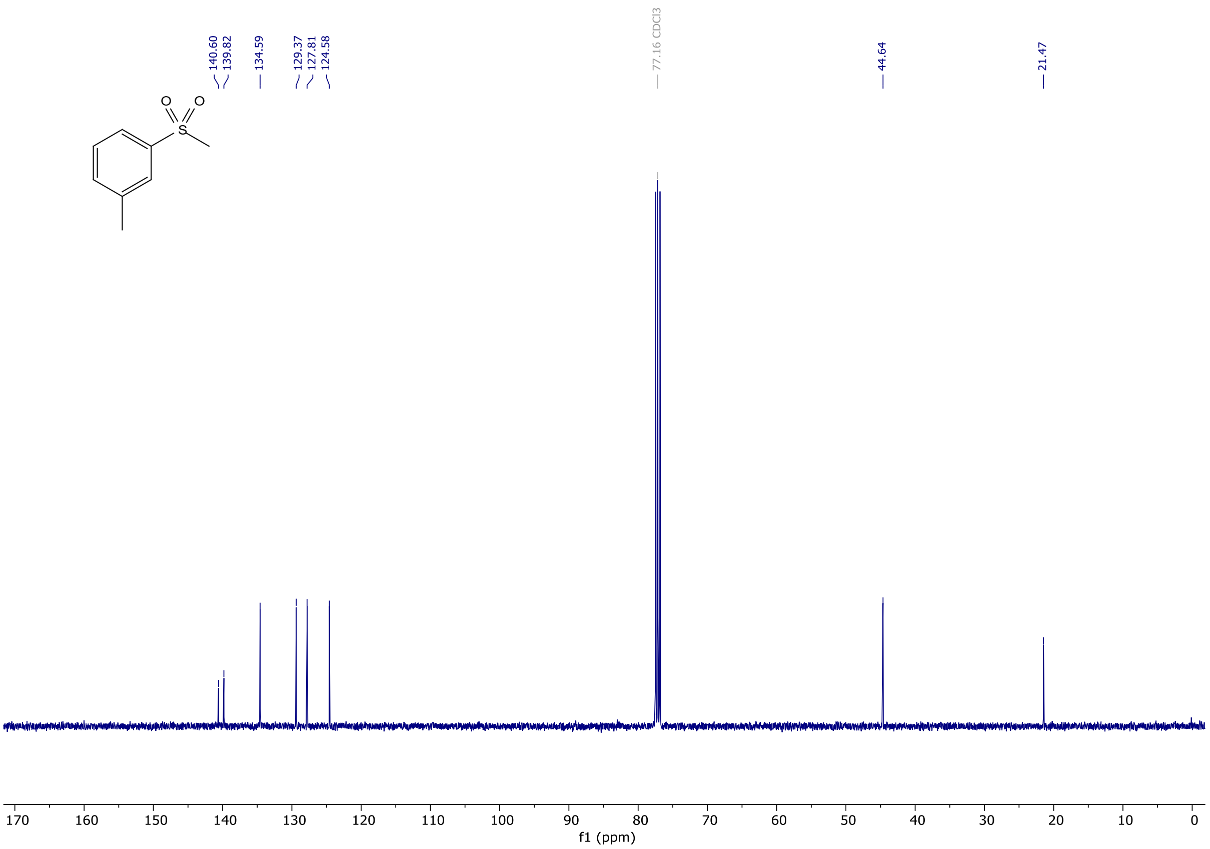

2-((propylsulfonyl)methyl)pyridine 1-oxide

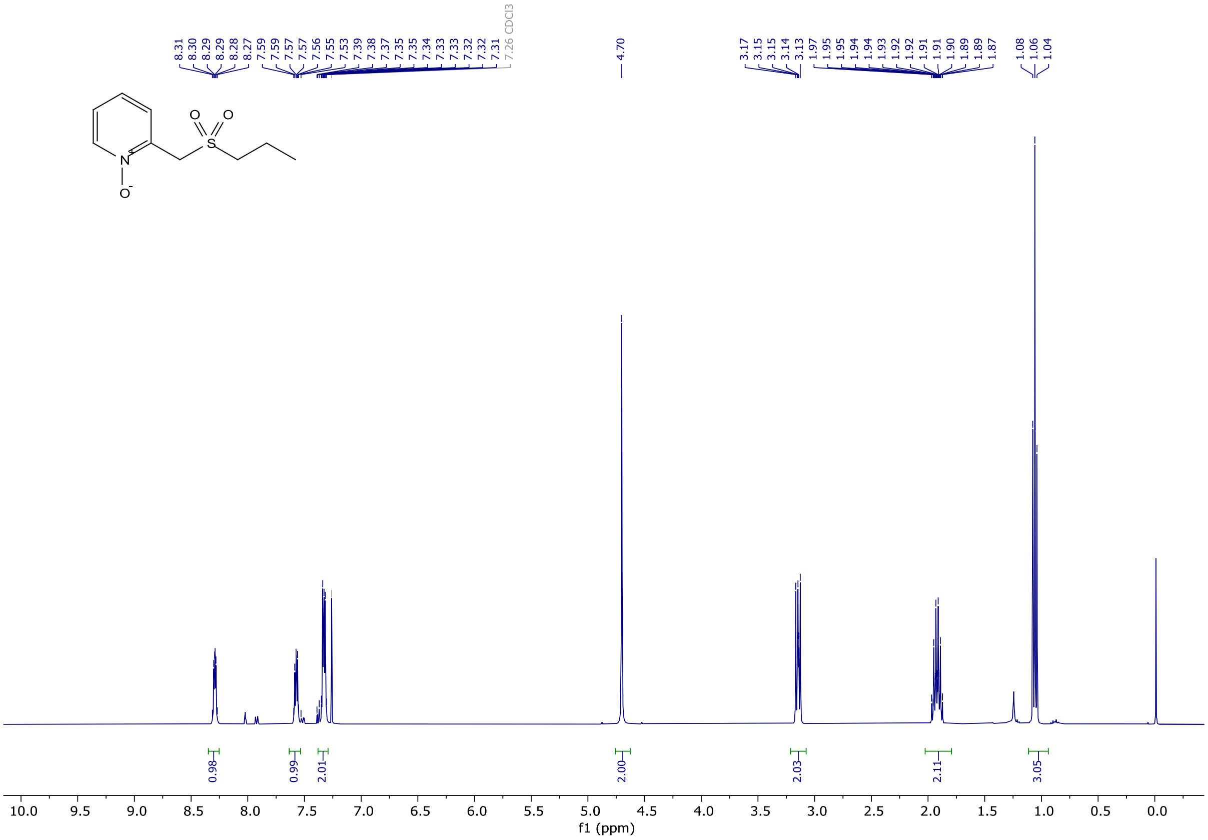

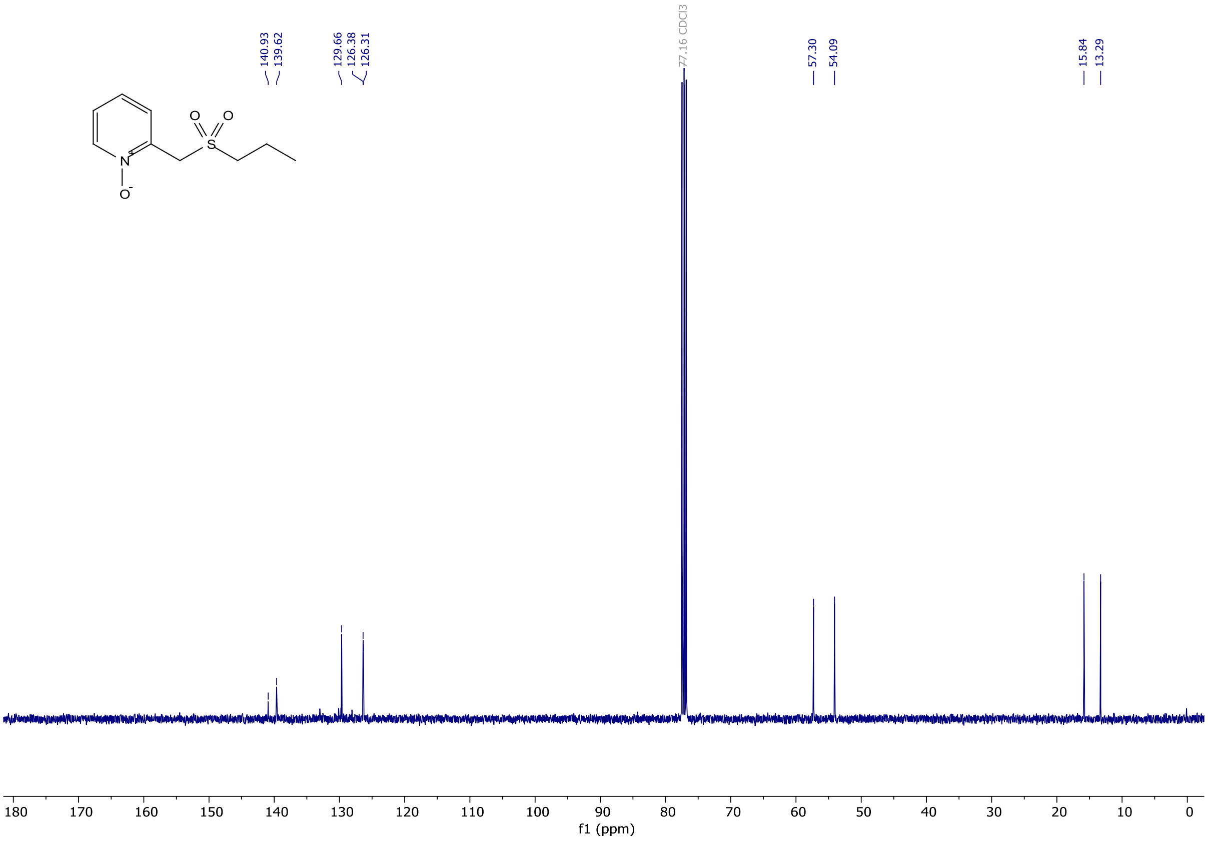

15. Copies of HRMS spectra for new compounds

2-((Propylthio)methyl)pyridine (4a)

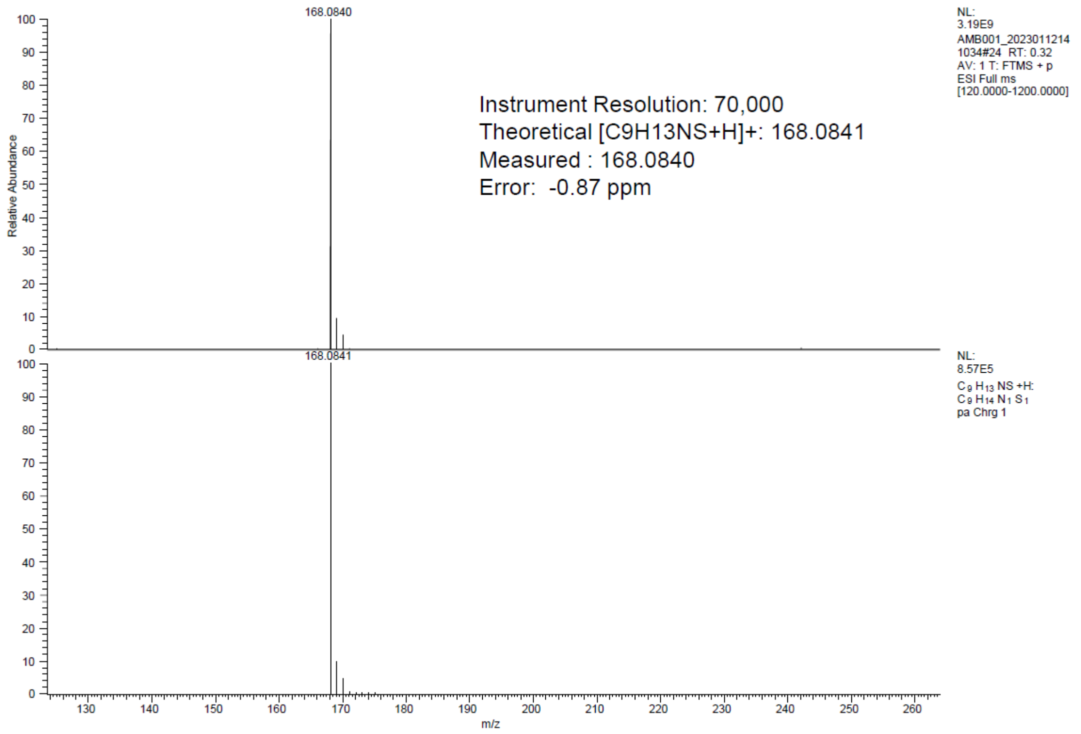

2-((Isobutylthio)methyl)pyridine (4c)

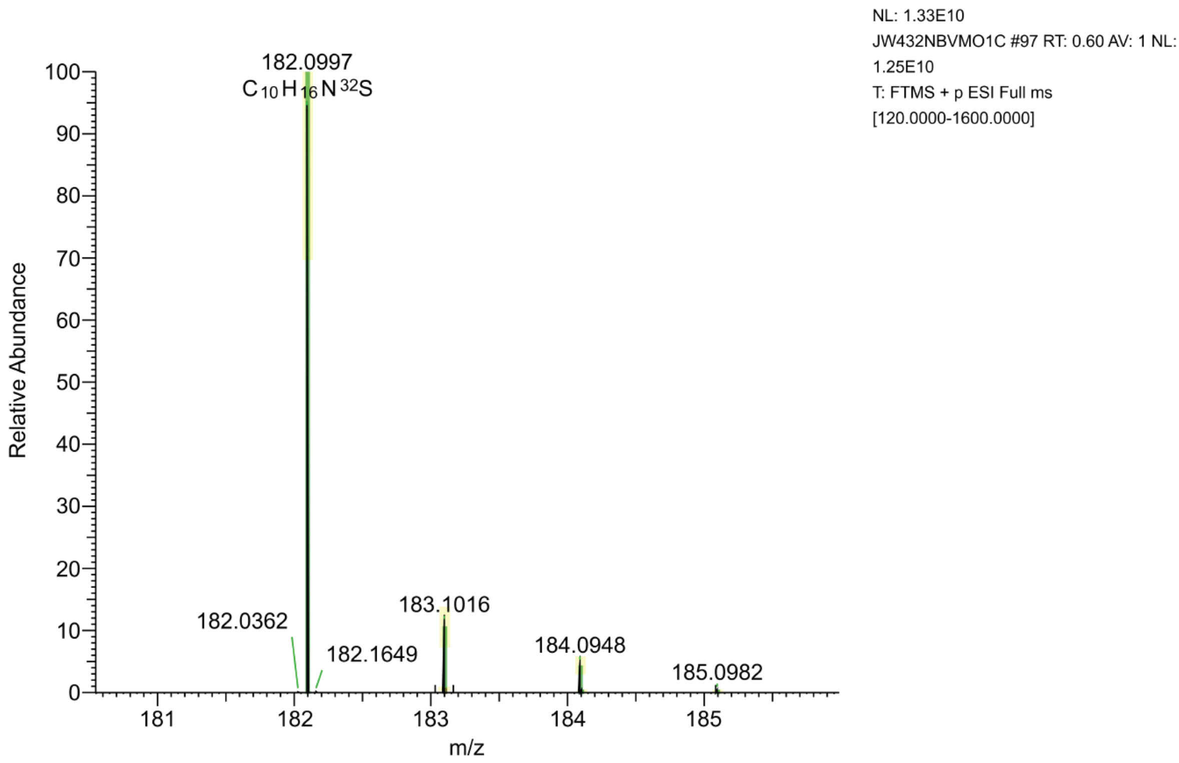

1-Methyl-2-(propylthio)-1H-imidazole (4g)

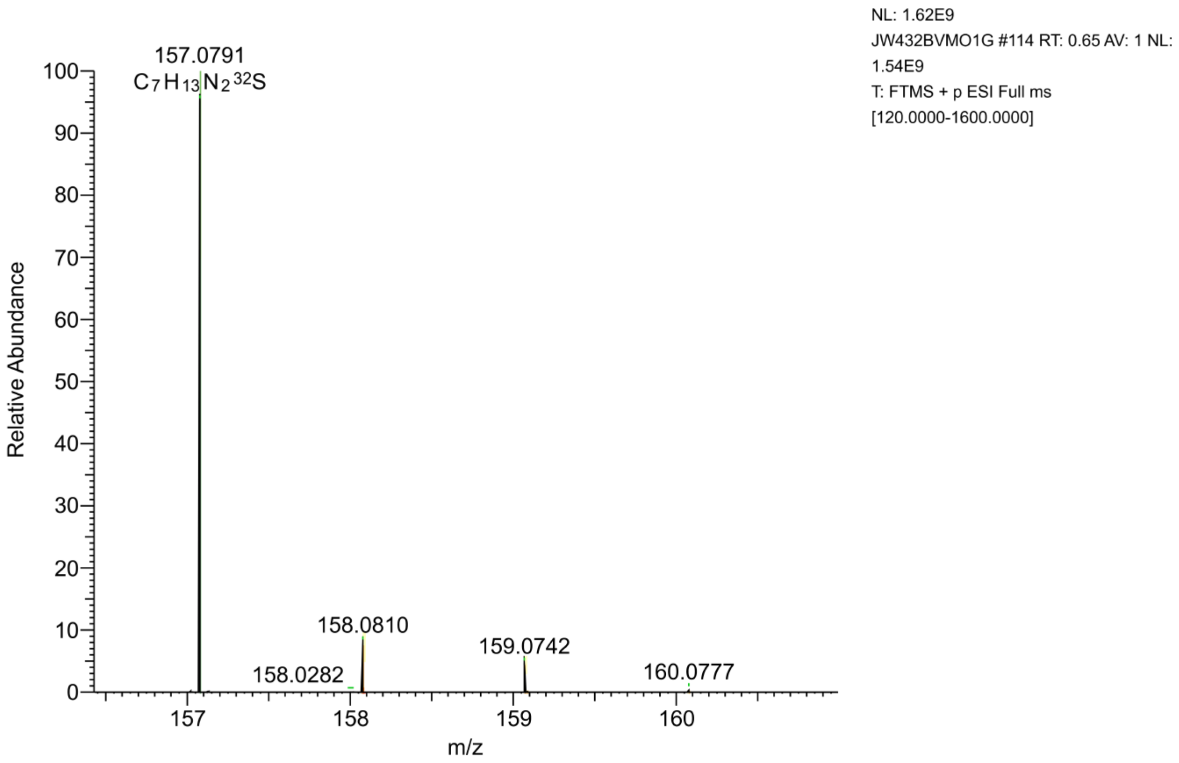

2-((Propylsulfinyl)methyl)pyridine (5a)

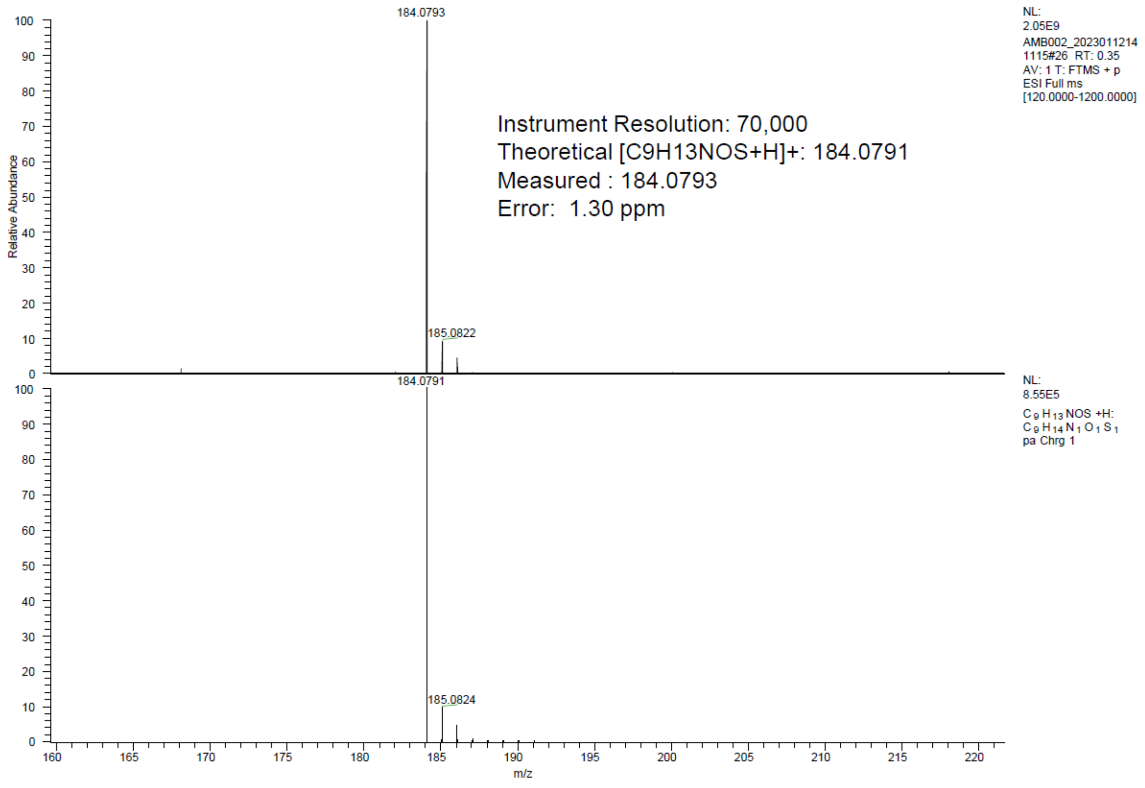

2-((Isobutylsulfinyl)methyl)pyridine (5c)

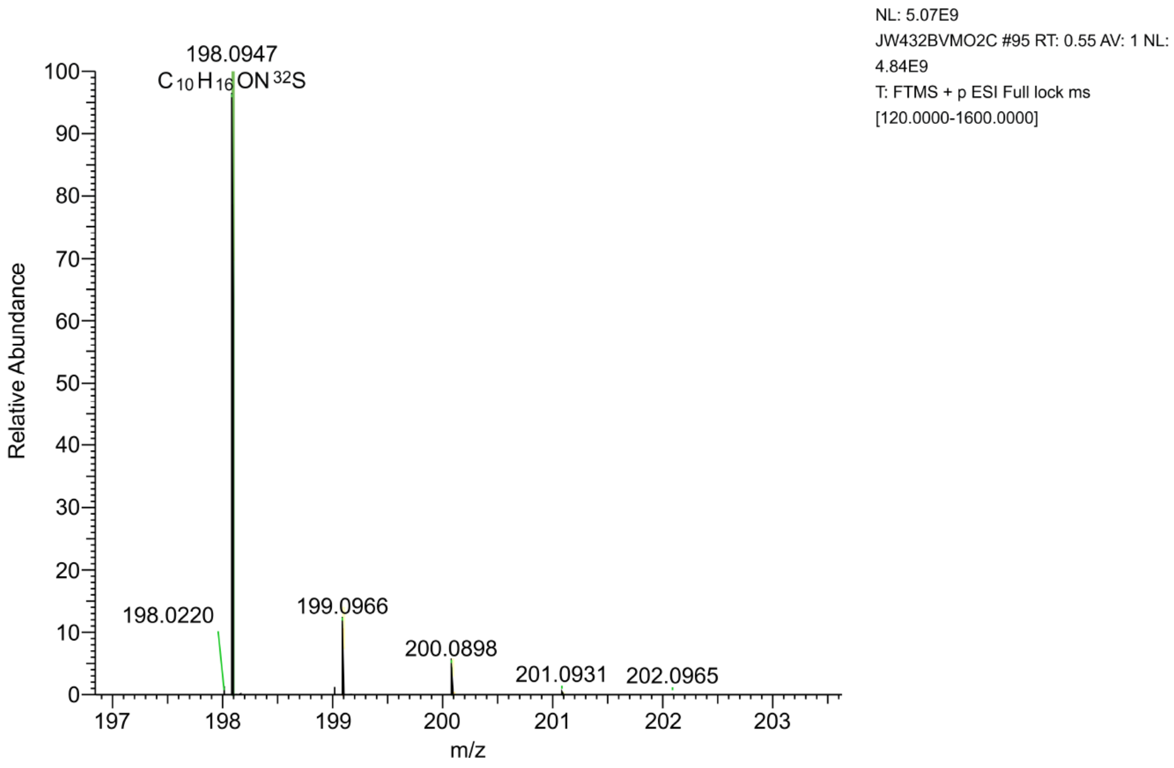

2-((Benzylsulfinyl)methyl)pyridine (5d)

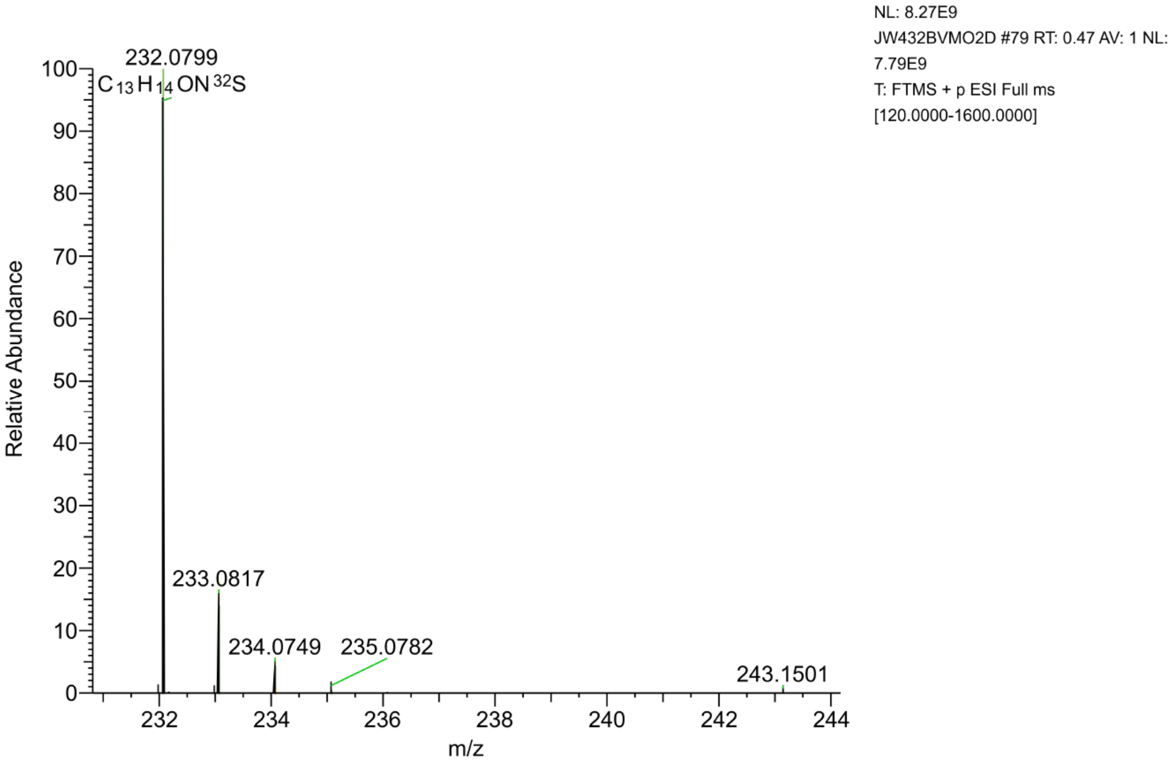

4-Methyl-2-(propylsulfinyl)pyridine (5f)

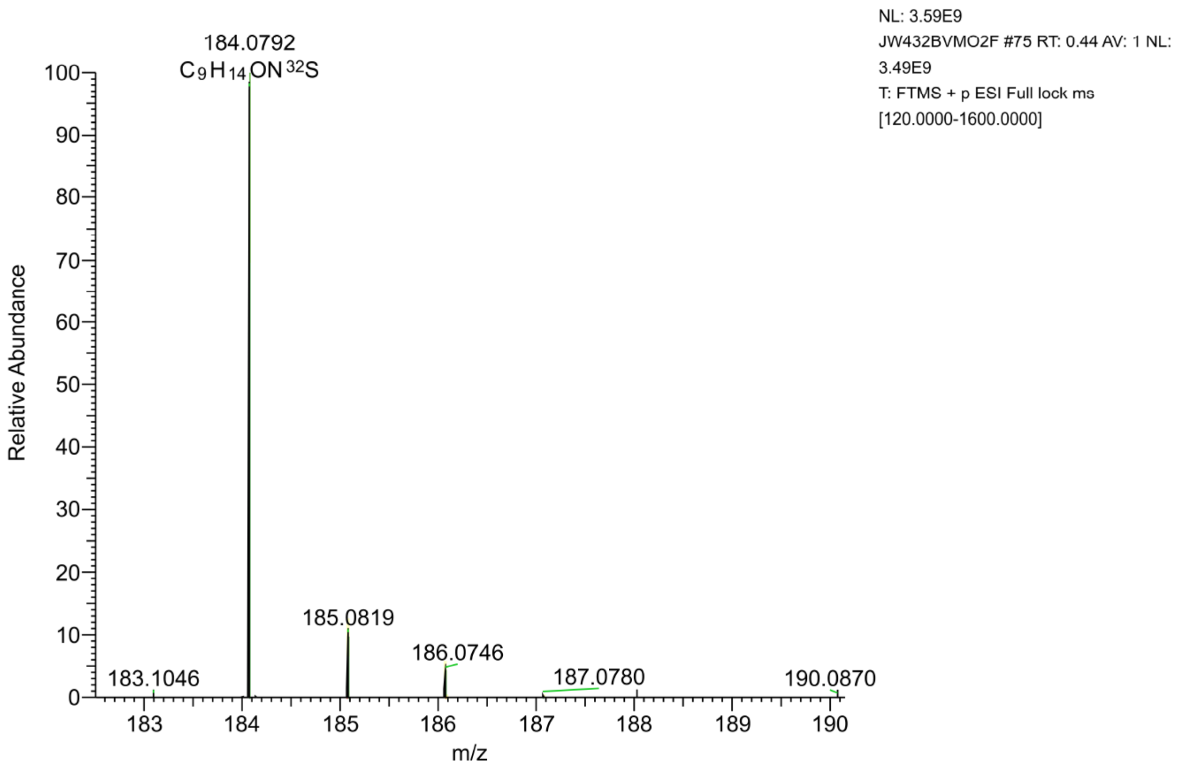

1-Methyl-2-(propylsulfinyl)-1H-imidazole (5g)

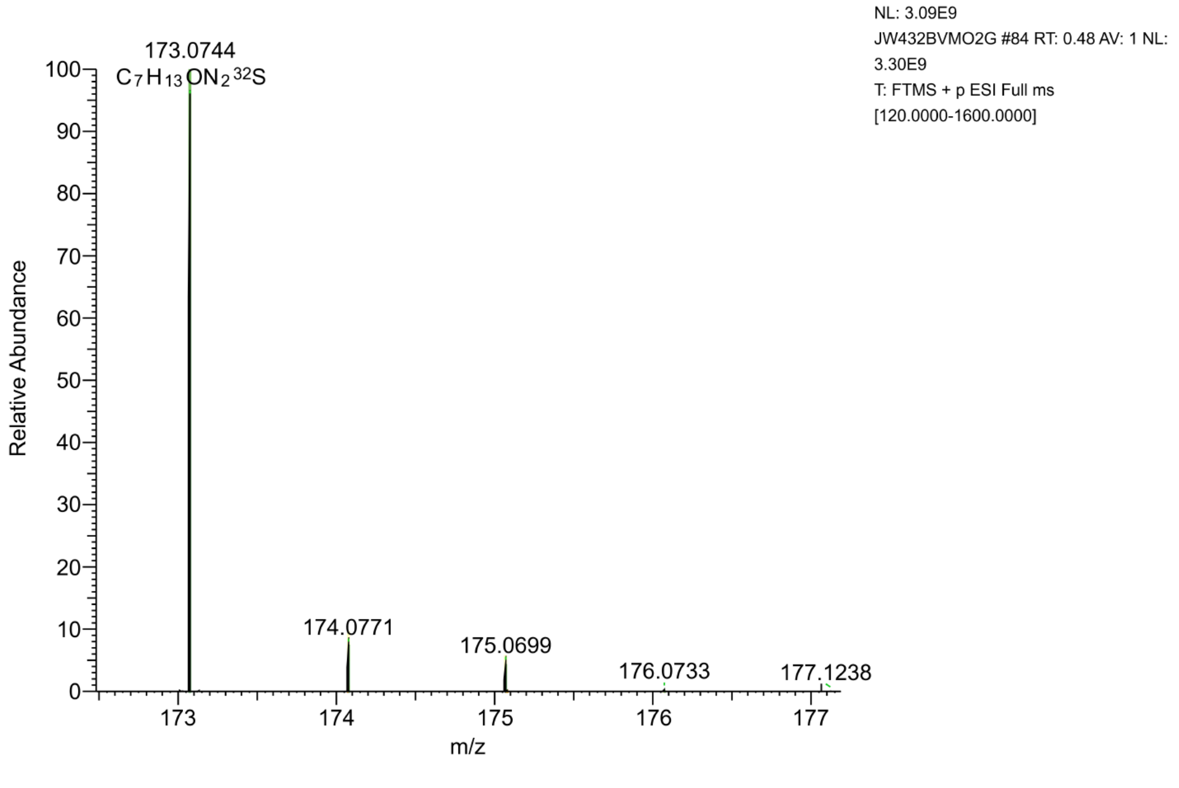

16. HPLC analysis and traces

The enantiomeric excess of the sulfoxides have been determined by HPLC using Daicel Chiralpak chiral columns IC (0.46 cm x 25 cm) and IG (0.46 cm x 25 cm) in normal phase. All the methods described ran at 1.0 mL/min, 25 ºC with an isocratic eluent. Detection wavelength were set at 254 nm for all compounds.

Compd	Column	Eluent system	Optical data α_D^{20} for new compounds
**5a**	IC	*n*-hexane:IPA 7:3	(***R***)-**5a** -4.0 (ee 64%, c 1, in CH_2Cl_2)<br>(***S***)-**5a** +12.1 (ee >99%, c 1, in CH_2Cl_2)
**5b**[11]	IC	*n*-hexane:IPA 6:4	
**5c**	IG	*n*-hexane:IPA 6:4	(***R***)-**5c** -5.6 (ee >99%, c 1, in CH_2Cl_2)<br>(***S***)-**5c** +27.1 (ee >99%, c 1, in CH_2Cl_2)
**5e**[12]	OD-H	*n*-hexane:IPA 9:1	
**5g**	IG	*n*-hexane:IPA 6:4	(***S***)-**5g** +10.1 (ee 76%, c 1, in CH_2Cl_2)
**5h**[11]	IC	*n*-hexane:IPA 8:2	
**5i**[11]	IC	*n*-hexane:IPA 8:2	
**5j**[11]	IC	*n*-hexane:IPA 8:2	
**5k**[11]	IC	*n*-hexane:IPA 8:2	
**5l**[11]	IC	*n*-hexane:IPA 8:2	
**5m**[11]	IG and IC	*n*-hexane:IPA 8:2	

[a]Absolute configurations were determined by comparison with literature.[11]

2-((Propylsulfinyl)methyl)pyridine (**5a**)

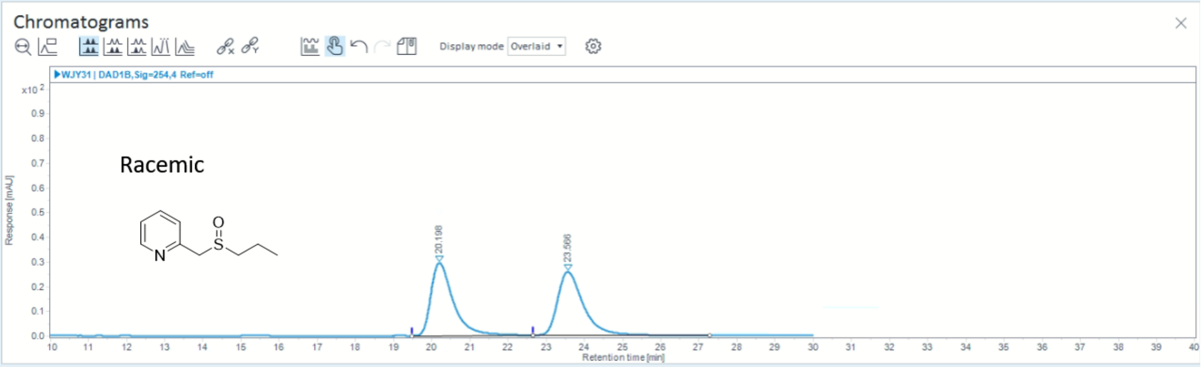

Injection Results

#	Name	Signal description	RT (min)	Area (mAU·s)	Area%	Height (mAU)	Height%	Amount	Concentration	Start time (min)	End time (min)
1		DAD1B,Sig=254,4 Ref=off	20.198	1196.839	49.827	29.491	53.45			19.485	22.642
2		DAD1B,Sig=254,4 Ref=off	23.566	1205.136	50.173	25.684	46.55			22.645	27.272

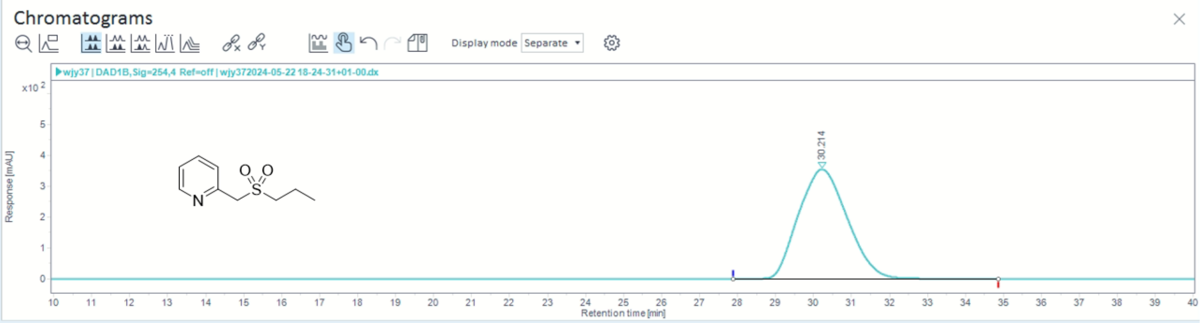

Injection Results

#	Name	Signal description	RT (min)	Area (mAU·s)	Area%	Height (mAU)	Height%	Amount	Concentration	Start time (min)	End time (min)
1		DAD1B,Sig=254,4 Ref=off	30.214	31490.527	100.000	354.507	100.00			27.872	34.857

Chromatograms

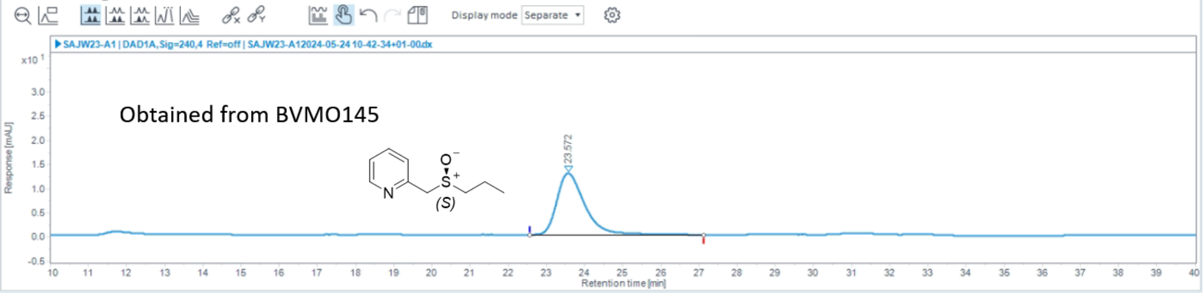

Injection Results

Peaks Summary

#	Name	Signal description	RT (min)	Area (mAU·s)	Area%	Height (mAU)	Height%	Amount	Concentration	Start time (min)	End time (min)
1		DAD1A,Sig=240,4 Ref=off	23.572	648.296	100.000	12.696	100.00			22.554	27.121

Chromatograms

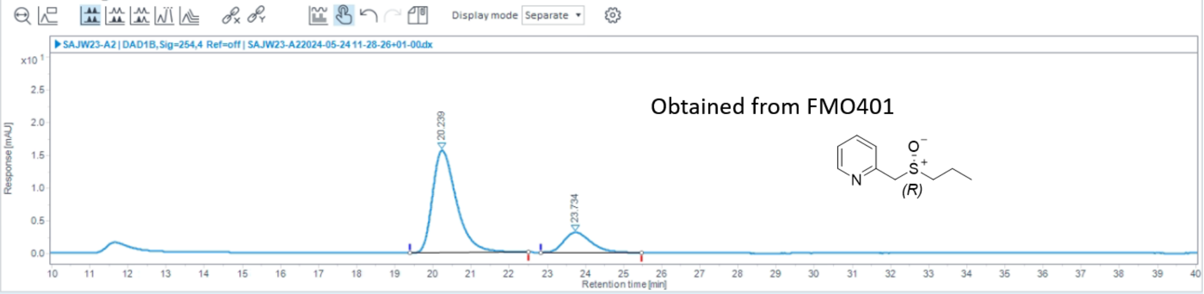

Injection Results

Peaks Summary

#	Name	Signal description	RT (min)	Area (mAU·s)	Area%	Height (mAU)	Height%	Amount	Concentration	Start time (min)	End time (min)
1		DAD1B,Sig=254,4 Ref=off	20.239	685.964	81.836	15.657	83.64			19.386	22.506
2		DAD1B,Sig=254,4 Ref=off	23.734	152.255	18.164	3.062	16.36			22.819	25.473

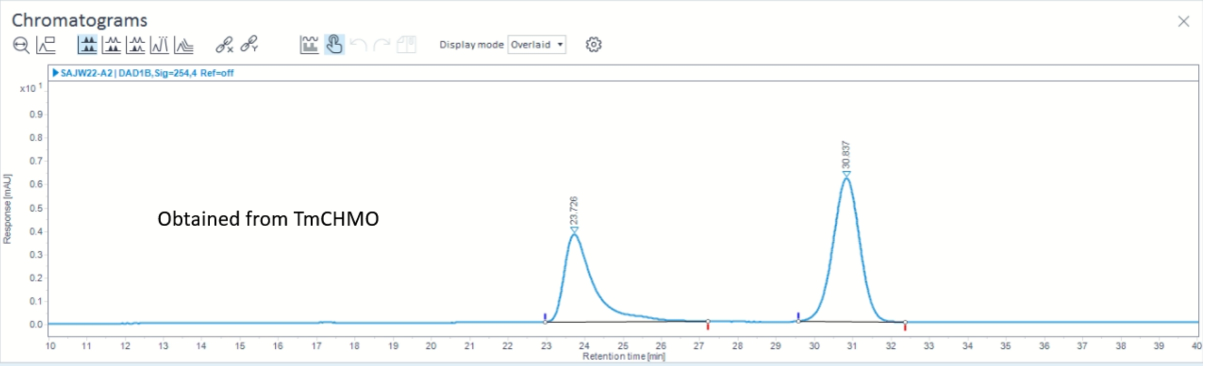

Injection Results

Peaks Summary

#	Name	Signal description	RT (min)	Area (mAU·s)	Area%	Height (mAU)	Height%	Amount	Concentration	Start time (min)	End time (min)
1		DAD1B,Sig=254,4 Ref=off	23.726	200.708	40.079	3.750	37.89			22.968	27.218
2		DAD1B,Sig=254,4 Ref=off	30.837	300.072	59.921	6.147	62.11			29.581	32.373

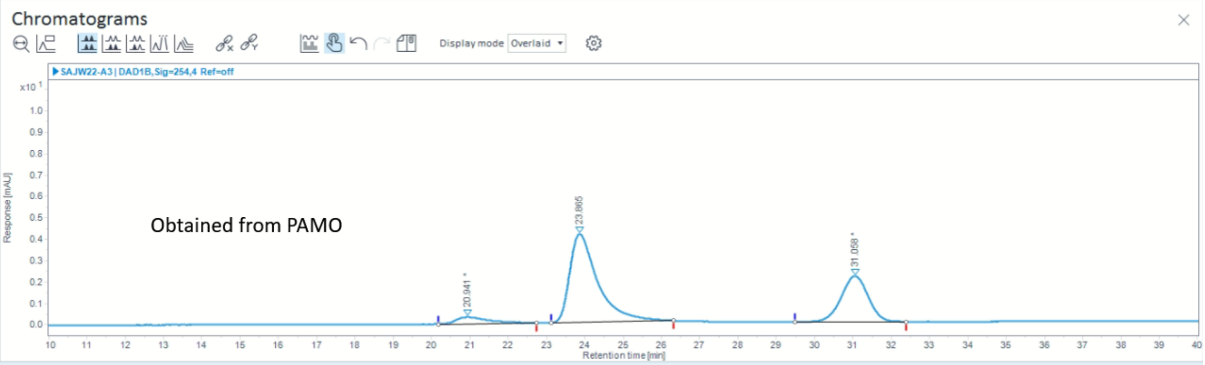

Injection Results

Peaks Summary

#	Name	Signal description	RT (min)	Area (mAU·s)	Area%	Height (mAU)	Height%	Amount	Concentration	Start time (min)	End time (min)
1		DAD1B,Sig=254,4 Ref=off	20.941	18.159	5.472	0.310	4.72			20.177	22.737
2		DAD1B,Sig=254,4 Ref=off	23.865	209.116	63.021	4.120	62.79			23.121	26.321
3		DAD1B,Sig=254,4 Ref=off	31.058	104.544	31.506	2.132	32.49			29.485	32.387

2-((Ethylsulfinyl)methyl)pyridine (**5b**)

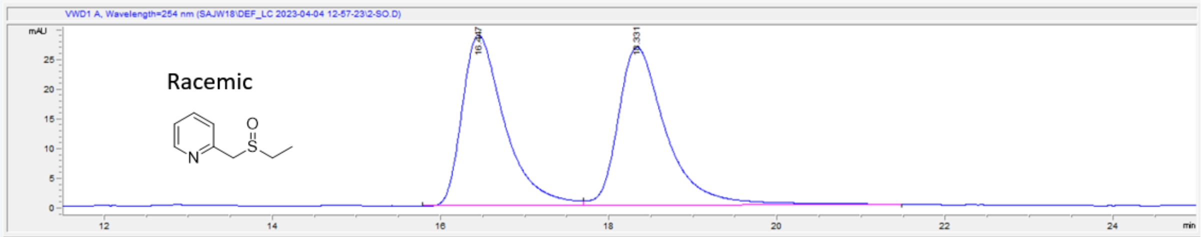

#	Time	Area	Height	Width	Symmetry
1	16.447	1016.1	28.8	0.5288	0.577
2	18.331	1076	26.6	0.5996	0.581

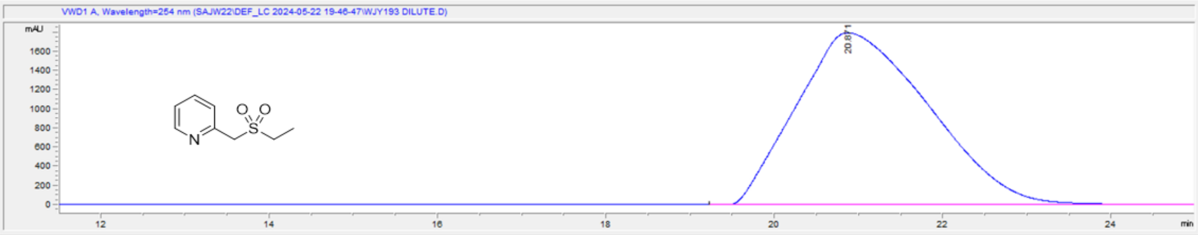

#	Time	Area	Height	Width	Symmetry
1	20.871	196617.7	1795.6	1.691	0.625

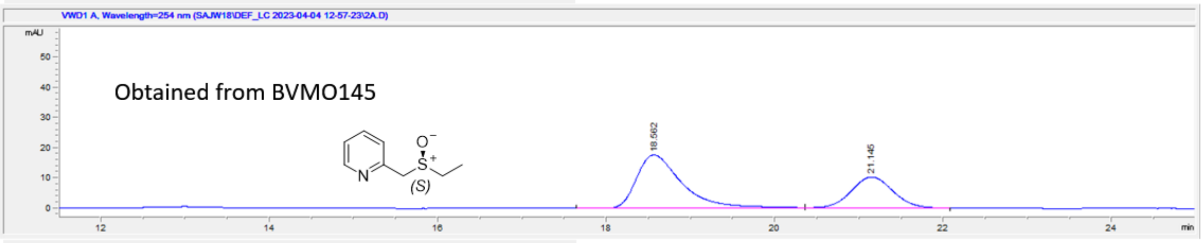

#	Time	Area	Height	Width	Symmetry
1	18.562	674.3	17.7	0.5721	0.547
2	21.145	369.8	10.4	0.5416	0.927

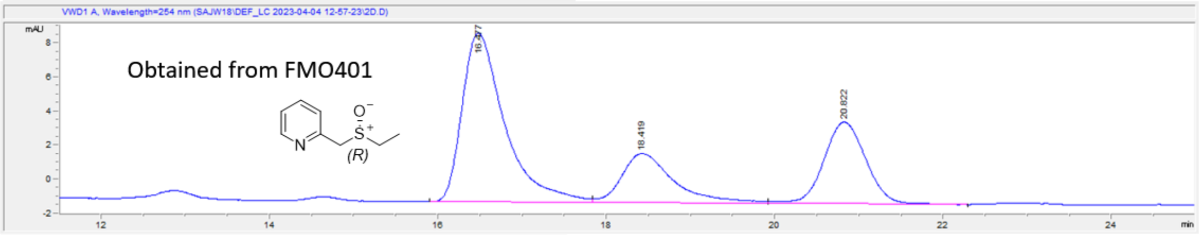

#	Time	Area	Height	Width	Symmetry
1	16.477	350.1	9.9	0.5273	0.558
2	18.419	126.4	2.9	0.6252	0.579
3	20.822	168.4	4.8	0.5474	0.913

2-((isobutylsulfinyl)methyl)pyridine (**5c**)

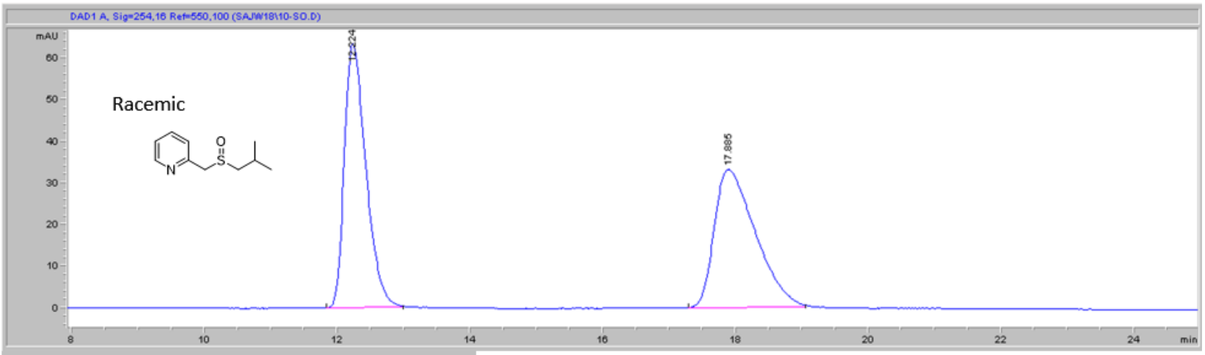

#	Time	Area	Height	Width	Area%	Symmetry
1	12.224	1428.4	63.4	0.3507	50.005	0.603
2	17.885	1428.1	33.2	0.6181	49.995	0.503

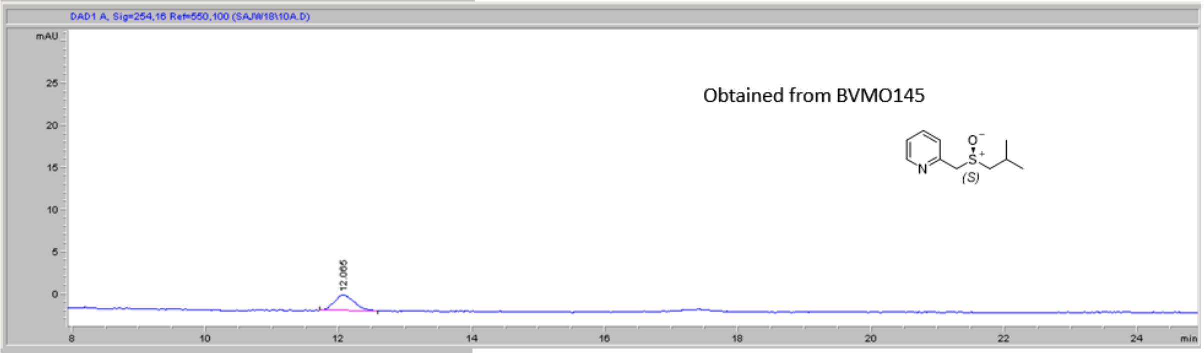

#	Time	Area	Height	Width	Area%	Symmetry
1	12.065	38.7	1.8	0.2791	100.000	0.806

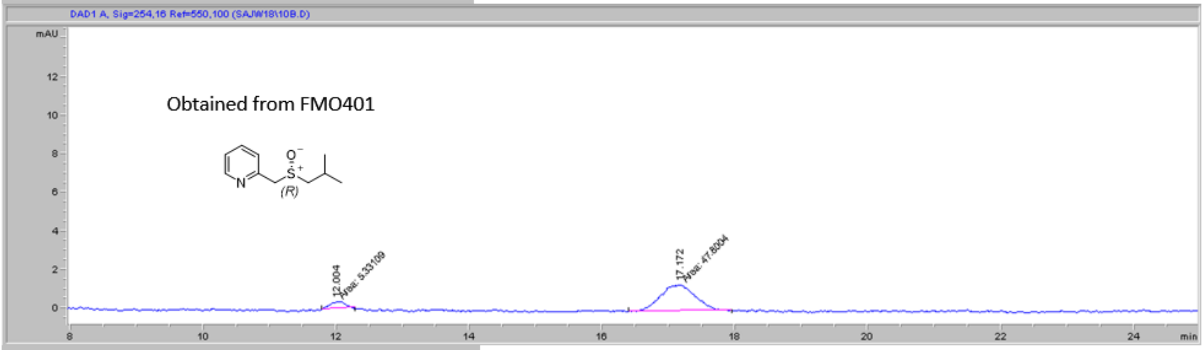

#	Time	Area	Height	Width	Area%	Symmetry
1	12.004	5.3	3.6E-1	0.245	10.034	0.844
2	17.172	47.8	1.3	0.6035	89.966	1.328

2-(propylsulfinyl)pyridine (**5e**)

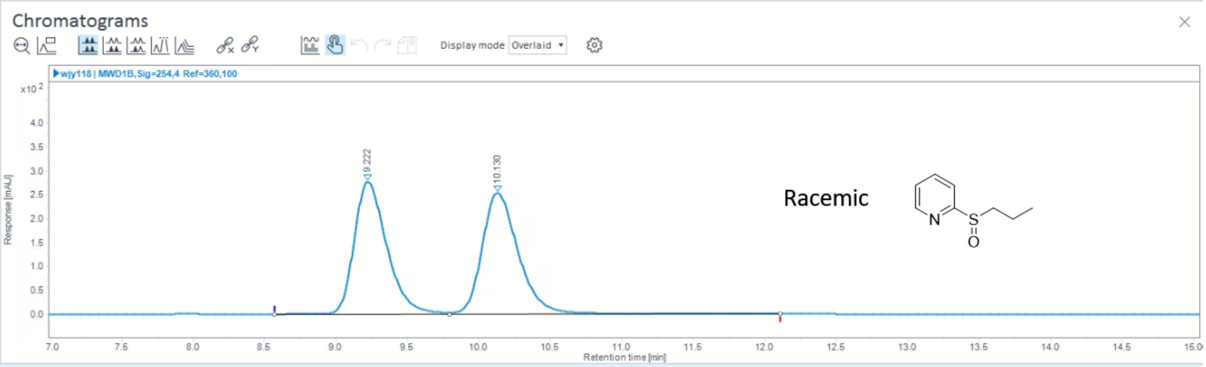

Injection Results

Peaks Summary

#	Name	Signal description	RT (min)	Area (mAU·s)	Area%	Height (mAU)	Height%	Amount	Concentration	Start time (min)	End time (min)
1		MWD1B,Sig=254,4 Ref=360,100	9.222	4403.906	49.521	277.144	52.19			8.570	9.793
2		MWD1B,Sig=254,4 Ref=360,100	10.130	4489.037	50.479	253.862	47.81			9.793	12.106

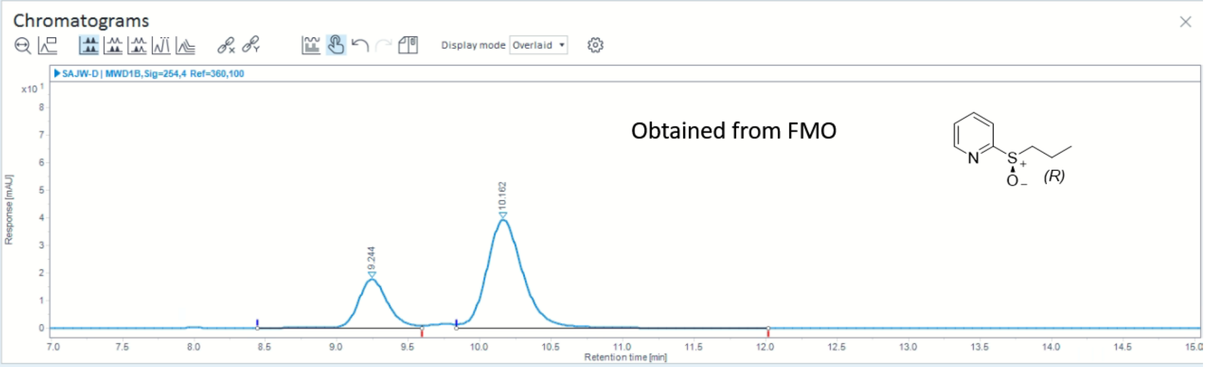

Injection Results

Peaks Summary

#	Name	Signal description	RT (min)	Area (mAU·s)	Area%	Height (mAU)	Height%	Amount	Concentration	Start time (min)	End time (min)
1		MWD1B,Sig=254,4 Ref=360,100	9.244	259.372	26.995	17.812	31.16			8.443	9.592
2		MWD1B,Sig=254,4 Ref=360,100	10.162	701.445	73.005	39.345	68.84			9.834	12.015

1-methyl-2-(propylsulfinyl)-1H-imidazole (**5g**)

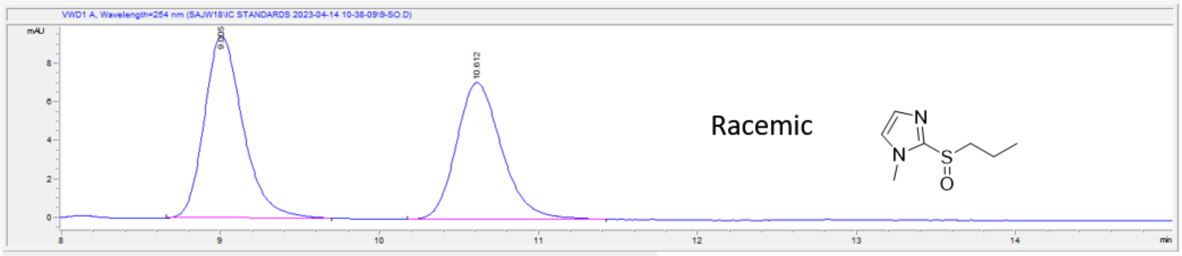

#	Time	Area	Height	Width	Area%	Symmetry
1	9.045	2837.5	179.3	0.2443	49.992	0.726
2	10.666	2838.4	147.9	0.2967	50.008	0.735

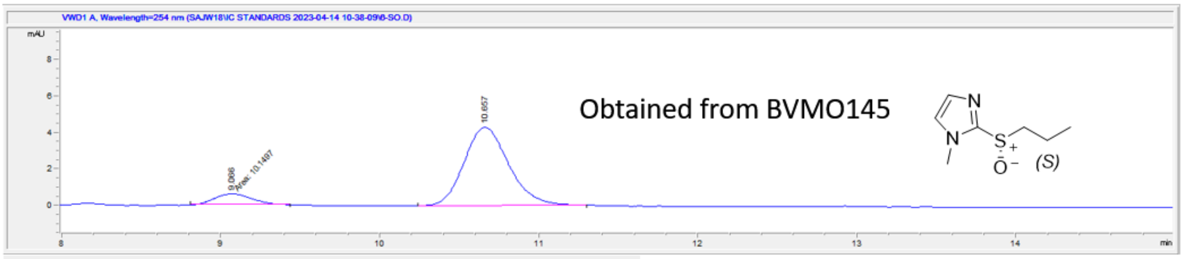

#	Time	Area	Height	Width	Area%	Symmetry
1	9.066	12.1	6.4E-1	0.271	12.222	0.703
2	10.658	86.6	4.3	0.3068	87.778	0.773

((Ethyl sulfinyl)methyl)benzene (**5h**)

Chromatograms

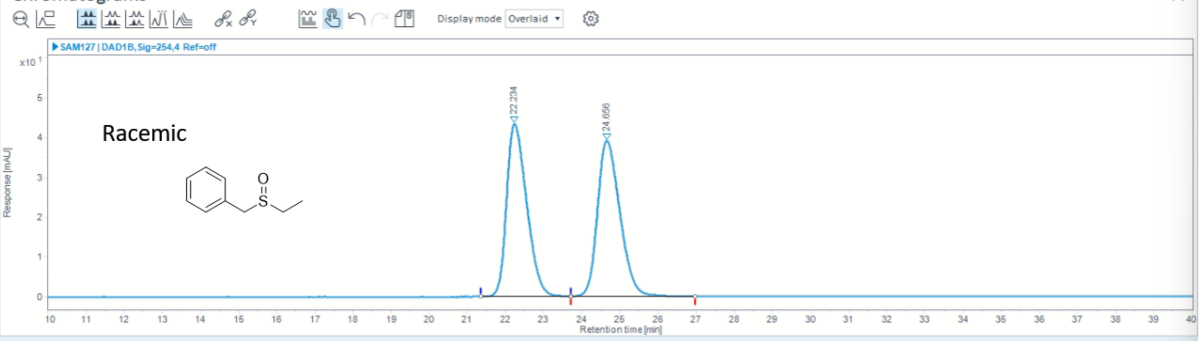

Injection Results

Peaks Summary

#	Name	Signal description	RT (min)	Area (mAU·s)	Area%	Height (mAU)	Height%	Amount	Concentration	Start time (min)	End time (min)
1		DAD1B,Sig=254,4 Ref=off	22.234	1579.513	49.778	43.277	52.56			21.356	23.703
2		DAD1B,Sig=254,4 Ref=off	24.656	1593.618	50.222	39.054	47.44			23.710	26.970

Chromatograms

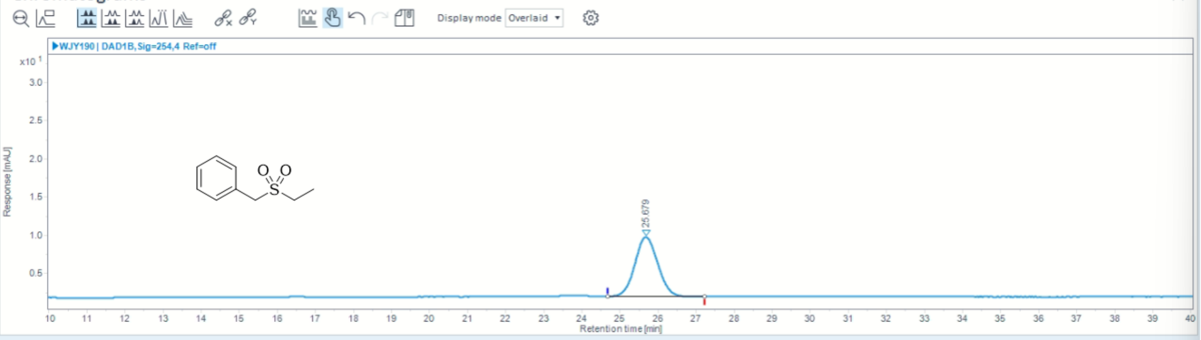

Injection Results

Peaks Summary

#	Name	Signal description	RT (min)	Area (mAU·s)	Area%	Height (mAU)	Height%	Amount	Concentration	Start time (min)	End time (min)
1		DAD1B,Sig=254,4 Ref=off	25.679	327.202	100.000	7.807	100.00			24.669	27.222

Chromatograms

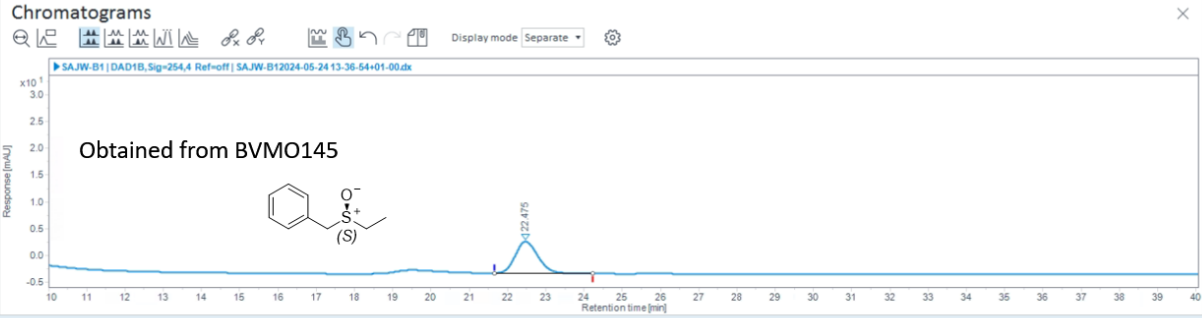

Injection Results

Peaks | Summary

#	Name	Signal description	RT (min)	Area (mAU·s)	Area%	Height (mAU)	Height%	Amount	Concentration	Start time (min)	End time (min)
1		DAD1B,Sig=254,4 Ref=off	22.475	245.786	100.000	5.913	100.00			21.663	24.223

Chromatograms

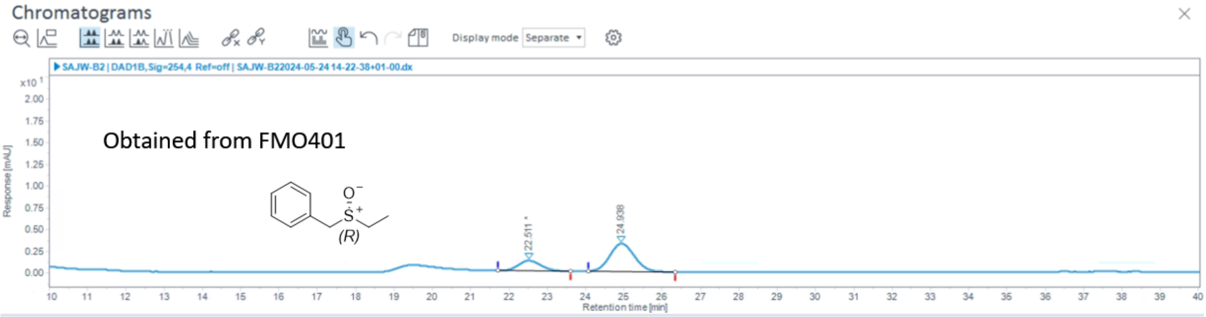

Injection Results

Peaks | Summary

#	Name	Signal description	RT (min)	Area (mAU·s)	Area%	Height (mAU)	Height%	Amount	Concentration	Start time (min)	End time (min)
1		DAD1B,Sig=254,4 Ref=off	22.511	46.462	24.631	1.151	26.60			21.699	23.596
2		DAD1B,Sig=254,4 Ref=off	24.938	142.174	75.369	3.177	73.40			24.081	26.348

Chromatograms

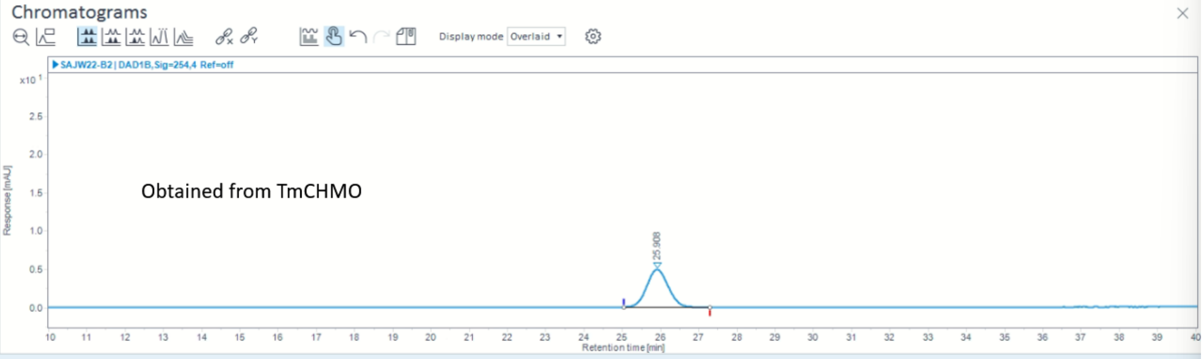

Injection Results

Peaks | Summary

#	Name	Signal description	RT (min)	Area (mAU·s)	Area%	Height (mAU)	Height%	Amount	Concentration	Start time (min)	End time (min)
1		DAD1B,Sig=254,4 Ref=off	25.908	191.368	100.000	4.909	100.00			25.040	27.293

Chromatograms

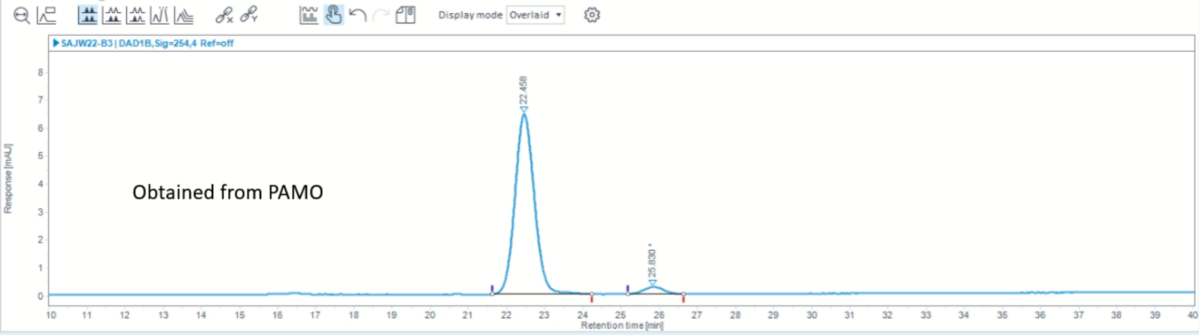

Injection Results

Peaks | Summary

#	Name	Signal description	RT (min)	Area (mAU·s)	Area%	Height (mAU)	Height%	Amount	Concentration	Start time (min)	End time (min)
1		DAD1B,Sig=254,4 Ref=off	22.458	227.565	96.036	6.437	96.17			21.613	24.240
2		DAD1B,Sig=254,4 Ref=off	25.830	9.394	3.964	0.256	3.83			25.184	26.647

1-Bromo-4-(ethylsulfinyl)benzene (**5i**)

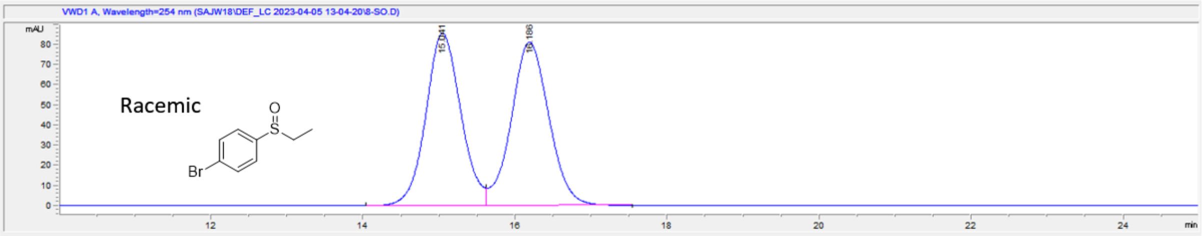

#	Time	Area	Height	Width	Area%	Symmetry
1	15.041	2753.3	85.9	0.4935	49.517	0.888
2	16.186	2807	81.3	0.5281	50.483	0.9

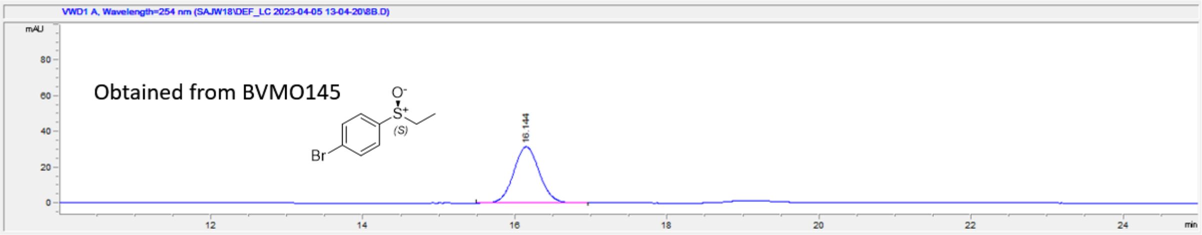

#	Time	Area	Height	Width	Area%	Symmetry
1	16.347	747.8	32.3	0.3604	100.000	0.947

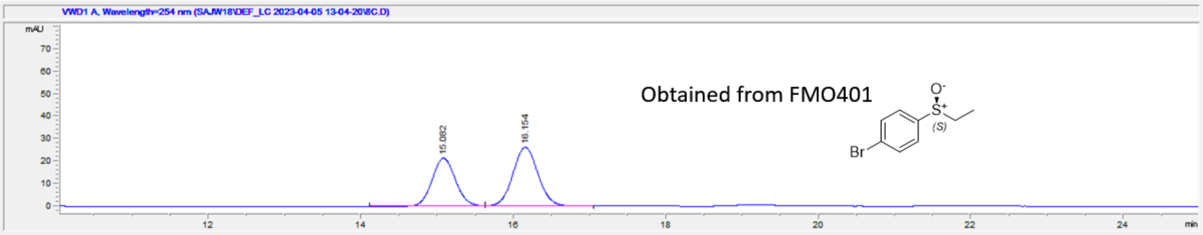

#	Time	Area	Height	Width	Area%	Symmetry
1	15.082	456.7	21.4	0.3338	43.231	0.916
2	16.153	599.7	26.1	0.3567	56.769	0.921

1-Chloro-3-(methylsulfinyl)benzene (**5j**)

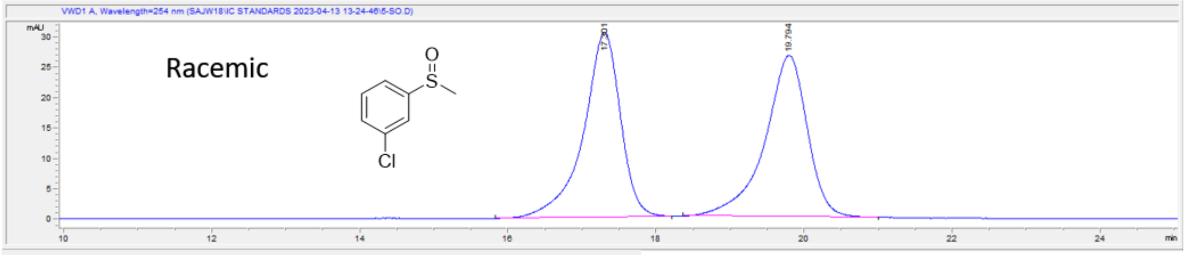

#	Time	Area	Height	Width	Area%	Symmetry
1	17.301	1059.5	30.4	0.5158	49.617	1.337
2	19.794	1075.9	26.6	0.6009	50.383	1.314

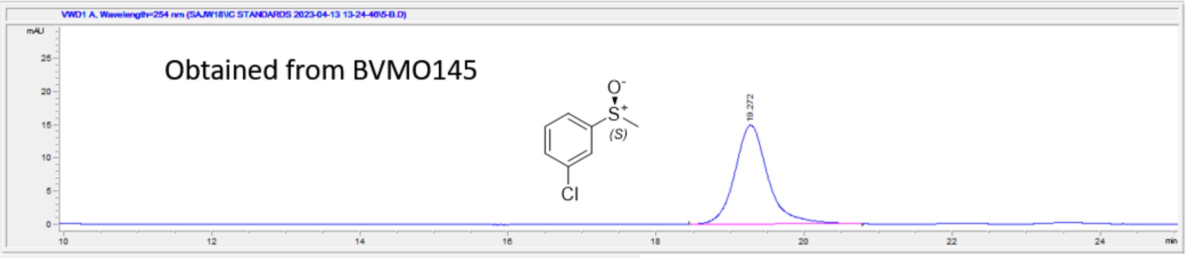

#	Time	Area	Height	Width	Area%	Symmetry
1	19.272	470.1	14.9	0.4721	100.000	0.833

1-Fluoro-4-(methylsulfinyl)benzene (**5k**)

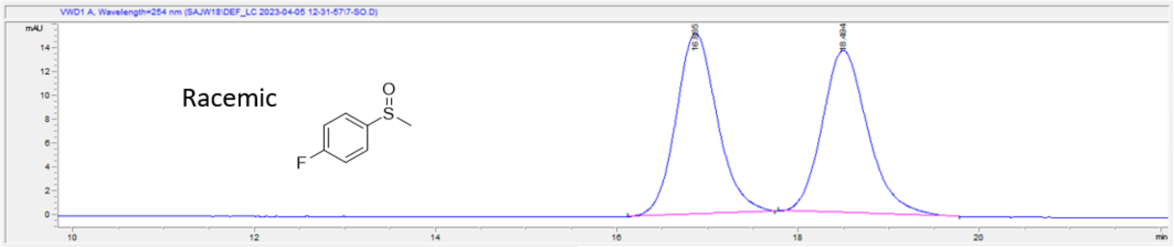

#	Time	Area	Height	Width	Area%	Symmetry
1	16.865	480.6	15.2	0.478	50.048	0.864
2	18.494	479.6	13.7	0.5318	49.952	0.81

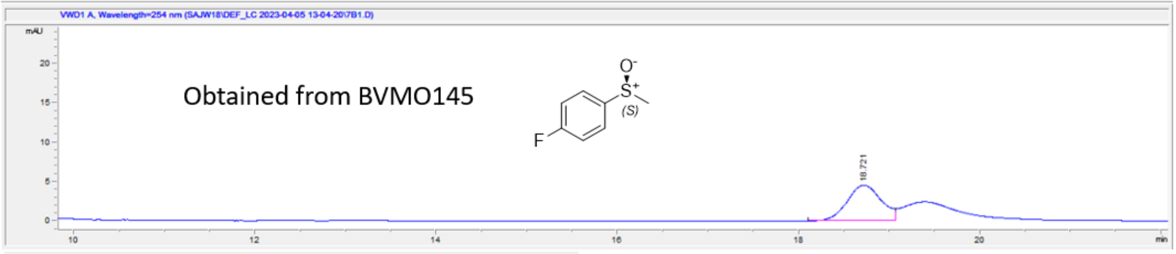

#	Time	Area	Height	Width	Area%	Symmetry
1	18.721	121.4	4.6	0.3953	100.000	0.958

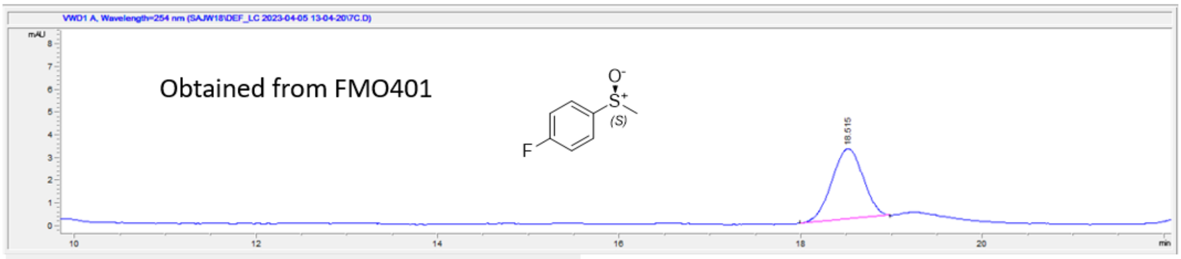

#	Time	Area	Height	Width	Area%	Symmetry
1	18.526	120.5	4.5	0.4083	100.000	0.907

(Methylsulfinyl)benzene (**5l**)

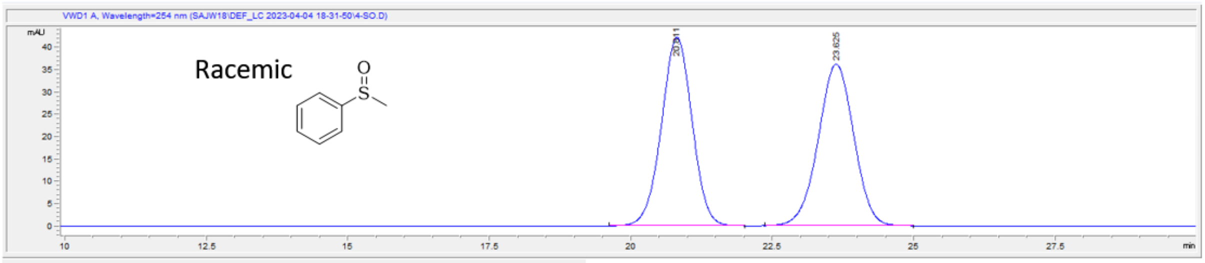

#	Time	Area	Height	Width	Area%	Symmetry
1	20.811	1598.4	42.3	0.5939	50.105	1.003
2	23.625	1591.7	36.3	0.6782	49.895	0.997

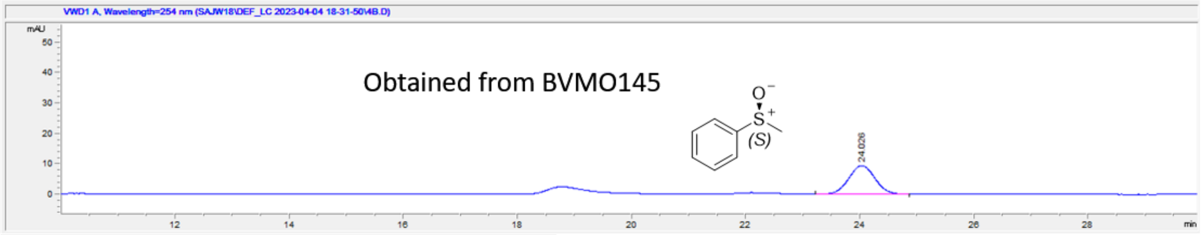

#	Time	Area	Height	Width	Area%	Symmetry
1	24.026	297.3	9.4	0.4836	100.000	0.954

1-Methyl-3-(methylsulfinyl)benzene (**5m**)

Analysed using Chiralpak IG column.

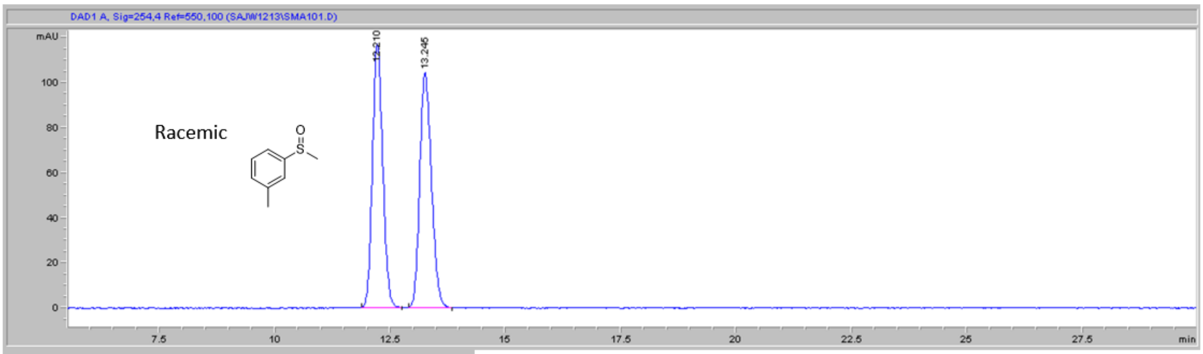

#	Time	Area	Height	Width	Area%	Symmetry
1	12.21	1822.5	117.3	0.2412	49.970	0.837
2	13.245	1824.7	104.2	0.2723	50.030	0.766

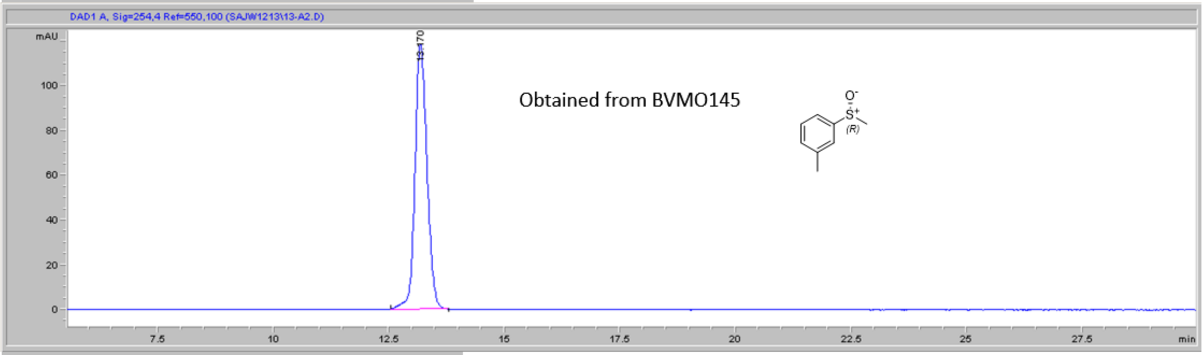

#	Time	Area	Height	Width	Area%	Symmetry
1	13.17	2151.9	118.8	0.2793	100.000	0.81

Analysed using Chiralpak IC column.

Chromatograms

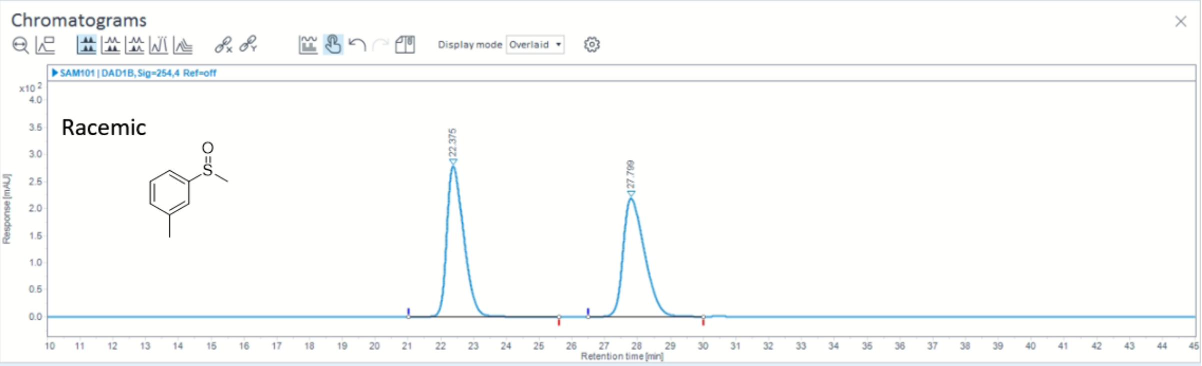

Injection Results

Peaks | Summary

#	Name	Signal description	RT (min)	Area (mAU·s)	Area%	Height (mAU)	Height%	Amount	Concentration	Start time (min)	End time (min)
1		DAD1B,Sig=254,4 Ref=off	22.375	9926.067	50.061	278.212	56.01			21.014	25.611
2		DAD1B,Sig=254,4 Ref=off	27.799	9901.812	49.939	218.491	43.99			26.501	29.994

Chromatograms

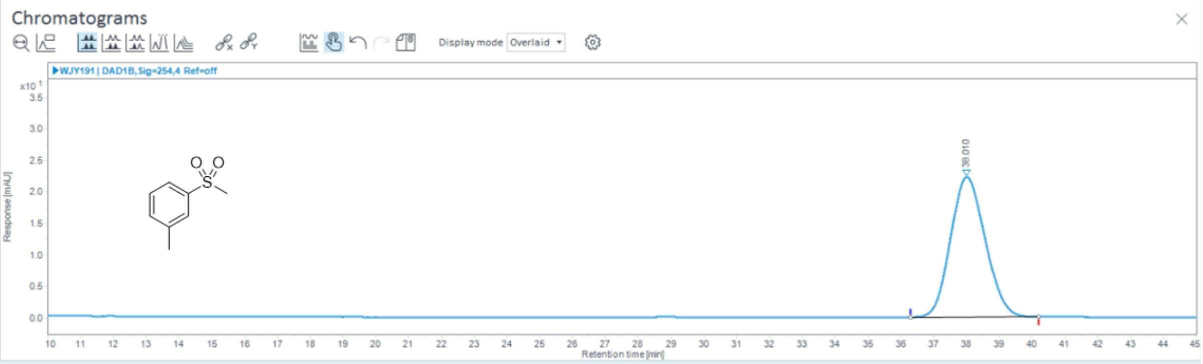

Injection Results

Peaks | Summary

#	Name	Signal description	RT (min)	Area (mAU·s)	Area%	Height (mAU)	Height%	Amount	Concentration	Start time (min)	End time (min)
1		DAD1B,Sig=254,4 Ref=off	38.010	1628.346	100.000	22.236	100.00			36.300	40.207

Chromatograms

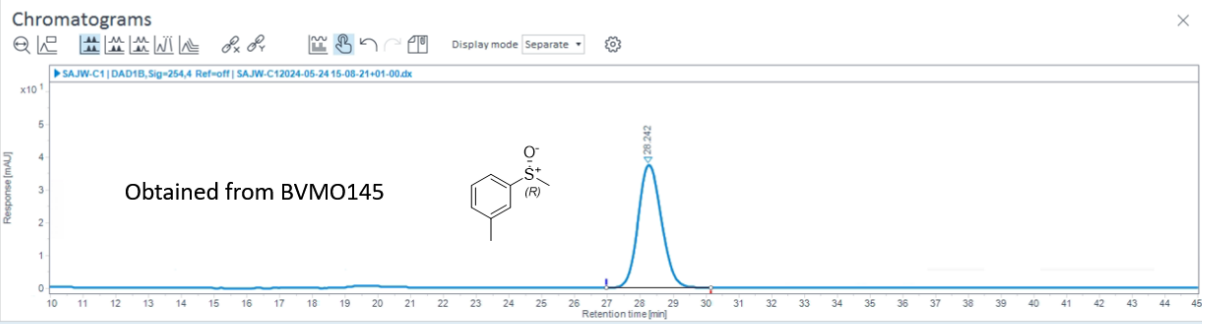

Injection Results

Peaks | Summary

#	Name	Signal description	RT (min)	Area (mAU·s)	Area%	Height (mAU)	Height%	Amount	Concentration	Start time (min)	End time (min)
1		DAD1B,Sig=254,4 Ref=off	28.242	1859.423	100.000	37.472	100.00			26.953	30.136

Chromatograms

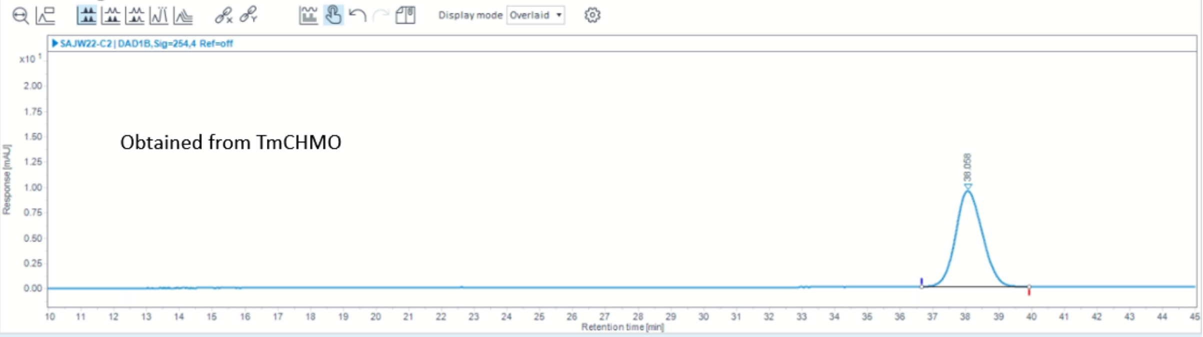

Injection Results

Peaks | Summary

#	Name	Signal description	RT (min)	Area (mAU·s)	Area%	Height (mAU)	Height%	Amount	Concentration	Start time (min)	End time (min)
1		DAD1B,Sig=254,4 Ref=off	38.058	540.457	100.000	9.483	100.00			36.656	39.940

Chromatograms

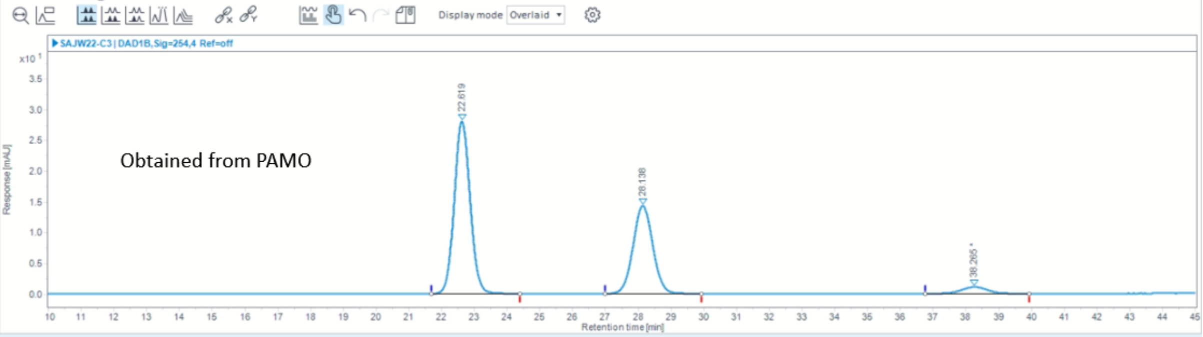

Injection Results

Peaks | Summary

#	Name	Signal description	RT (min)	Area (mAU·s)	Area%	Height (mAU)	Height%	Amount	Concentration	Start time (min)	End time (min)
1		DAD1B,Sig=254,4 Ref=off	22.619	942.259	58.856	28.030	64.57			21.681	24.398
2		DAD1B,Sig=254,4 Ref=off	28.138	599.524	37.448	14.321	32.99			26.988	29.941
3		DAD1B,Sig=254,4 Ref=off	38.265	59.174	3.696	1.061	2.44			36.742	39.919

17. References

1. M. Mirdita, K. Schütze, Y. Moriwaki, L. Heo, S. Ovchinnikov and M. Steinegger, *Nat. Methods,* 2022, **19**, 679–682.
2. G. M. Morris, D. S. Goodsell, R. S. Halliday, R. Huey, W. E. Hart, R. K. Belew and A. J. Olson, *J. Comput. Chem.,* 1998, **19**, 1639–1662.
3. V. Hornak, R. Abel, A. Okur, B. Strockbine, A. Roitberg and C. Simmerling, *Proteins,* 2006, **65**, 712–725.
4. T. Darden, D. York, L. Pedersen and *J. Chem. Phys.,* 1993, **98**, 10089–10092.
5. J. H. Wallenstein, J. Sundberg, C. J. McKenzie and M. Abrahamsson, *Eur. J. Inorg. Chem.*, 2016, **2016**, 897–906.
6. D. Wang, Z. Liu, Z. Wang, X. Ma and P. Yu, *Green Chem.*, 2019, **21**, 157–163.
7. A. T. Londregan, S. Jennings and L. Wei, *Org. Lett.*, 2011, **13**, 1840–1843.
8. D. M. Andrews, T. C. M. Page, J. M. Peach and A. J. Pratt, *J. Chem. Soc., Perkin Trans. 1*, 1995, 1045–1048.
9. S. Kotha, N. K. Gupta and S. Ansari, *RSC Adv.*, 2022, **12**, 25154–25162.
10. N. Sakai, H. Maeda and Y. Ogiwara, *Synthesis*, 2019, **51**, 2323–2330.
11. S. Anselmi, A. T. P. Carvalho, A. Serrano-Sanchez, J. L. Ortega-Roldan, J. Caswell, I. Omar, G. Perez-Ortiz, S. M. Barry, T. S. Moody and D. Castagnolo, *ACS Catal.*, 2023, **13**, 4742–4751.
12. W. Dai, S. Shang, Y. Lv, G. Li, C. Li and S. Gao, *ACS Catal.*, 2017, **7**, 4890–4895.
13. V. Nosek and J. Míšek, *Angew. Chem., Int. Ed.*, 2018, **130**, 9849–9852.
14. S. S. Kim, K. Nehru, S. S. Kim, D. W. Kim and H. C. Jung, *Synthesis*, 2002, 2484–2486.
15. M. Yadav, A. Bhunia, S. K. Jana and P. W. Roesky, *Inorg. Chem.*, 2016, **55**, 28.
16. X.-N. Zou, D. Zhang, T.-X. Luan, Q. Li, L. Li, P.-Z. Li and Y. Zhao, *ACS Appl. Mater. Interfaces*, 2021, **13**, 20137–20144.
17. N. Amri and T. Wirth, *J. Org. Chem.*, 2021, **86**, 15961–15972.
18. S. C. Davidson, G. dos P. Gomes, L. R. Kuhn, I. v. Alabugin, A. R. Kennedy and N. C. O. Tomkinson, *Tetrahedron*, 2021, **78**, 1–10.
19. D. Nagornîi, F. Raymenants, N. Kaplaneris and T. Noel, ChemRxiv. 2024; doi:10.26434/chemrxiv-2024-djx7b. This content is a preprint and has not been peer-reviewed.
20. A. Shavnya, S. B. Coffey, A. C. Smith and V. Mascitti, *Org. Lett.* 2013, **15**, 6226–6229.
